# Predicting the effects of spatiotemporal modifications of muscle activation on the tentacle extension in squid

**DOI:** 10.3389/fbioe.2023.1193409

**Published:** 2023-10-19

**Authors:** Johan L. van Leeuwen, William M. Kier

**Affiliations:** ^1^ Experimental Zoology Group, Department of Animal Sciences, Wageningen University, Wageningen, Netherlands; ^2^ Department of Biology, University of North Carolina, Chapel Hill, NC, United States

**Keywords:** muscle control, muscle model, cephalopods, prey capture, robustness, sensitivity analysis

## Abstract

Squid use eight arms and two slender tentacles to capture prey. The muscular stalks of the tentacles are elongated approximately 80% in 20–40 ms towards the prey, which is adhered to the terminal clubs by arrays of suckers. Using a previously developed forward dynamics model of the extension of the tentacles of the squid *Doryteuthis pealeii* (formerly *Loligo pealeii*), we predict how spatial muscle-activation patterns result in a distribution of muscular power, muscle work, and kinetic and elastic energy along the tentacle. The simulated peak extension speed of the tentacles is remarkably insensitive to delays of activation along the stalk, as well as to random variations in the activation onset. A delay along the tentacle of 50% of the extension time has only a small effect on the peak extension velocity of the tentacle compared with a zero-delay pattern. A slight delay of the distal portion relative to the proximal has a small positive effect on peak extension velocity, whereas negative delays (delay reversed along stalk) always reduce extension performance. In addition, tentacular extension is relatively insensitive to superimposed random variations in the prescribed delays along the stalk. This holds in particular for small positive delays that are similar to delays predicted from measured axonal diameters of motor neurons. This robustness against variation in the activation distribution reduces the accuracy requirements of the neuronal control and is likely due to the non-linear mechanical properties of the muscular tissue in the tentacle.

## 1 Introduction

Squid and cuttlefish capture and subdue their prey using a pair of extensible tentacles and four pairs of arms (Messenger, 1968; Messenger, 1977; Kier, 1982). Rapid elongation of the proximal portion of the tentacles, termed the stalk, brings the terminal club and its suckers in contact with the prey. During a successful strike, the suckers attach to the prey and then the stalks shorten, bringing the prey within reach of the arms, which subdue and manipulate the prey for ingestion. The tentacle strike by squid is remarkably rapid. High-speed cinematography of prey capture by the squid *Doryteuthis pealeii* (formerly *Loligo pealeii*) reveals that the stalks elongate by approximately 50%–80% in only 20–40 ms, reaching peak velocities of over 2 m s^−1^ and peak accelerations of approximately 250 m s^−2^ (25.5 g) (Kier and Van Leeuwen, 1997).

**FIGURE 1:**
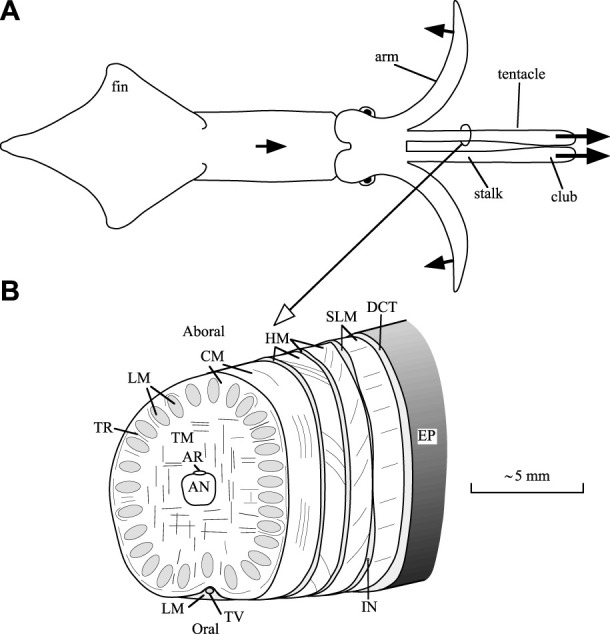
Diagrams of a squid and tentacular stalk morphology. **(A)** Outline drawing of a dorsal view a squid, showing only two of the eight arms. During prey capture, the squid swims forward, bends six arms outward and backward, and elongates the muscular stalks of the tentacles in 20–40 ms. The protruded tentacular clubs adhere to the prey with suckers. Initially, two arms stay aligned with the tentacles to prevent buckling (Kier and Van Leeuwen, 1997). Arrows with solid heads indicate directions of motion. **(B)** Scheme of the internal morphology of the tentacular stalk (based on Kier, 1982). Abbreviations: AN, axial nerve cord; AR, artery; CM, circular muscles, DCT, dermal connective tissue; EP, epithelium; HM, helical muscle; IN, intramuscular nerve cord; LM, longitudinal muscle; SLM, superficial longitudinal muscle; TR, trabeculae of transverse muscle; TM, transverse muscle; TV, superficial tentacular vein. After Van Leeuwen and Kier (1997).

Support and transmission of force required for the movements of the arms and tentacles of squid is achieved with a type of dynamic skeleton referred to as a muscular hydrostat (Kier and Smith, 1985). This system, which is also present in a variety of invertebrate structures, many vertebrate tongues and in the trunk of the elephant, is characterized by a tightly packed three-dimensional array of muscle fibers. No rigid internal or external skeletal elements are present as is observed, for instance, in vertebrates or arthropods. In addition, these structures lack the fluid-filled cavities that characterize hydrostatic skeletal support in many invertebrates (Kier, 2012). Muscle tissue exhibits high bulk modulus, similar to most animal tissues that lack gas-filled spaces, and it therefore resists changes in volume. Support and movement in these structures is thus achieved by controlling the three-dimensional shape, because any decrease in one dimension must result in an increase in another. In the case of the tentacles, which are the focus of this paper, elongation is created by transverse and circular muscle fibers (Kier (1982); Figure 1 shows a diagram of the internal tentacle morphology). The transverse muscle fibers occupy the central core of the tentacles and are continuous with the surrounding thin circular muscle layer. Muscle fibers in the transverse and circular musculature are oriented in planes perpendicular to the long axis of the tentacles. Their contraction decreases the diameter of the tentacular stalk, and because the stalk is essentially constant in volume, the length of the stalk must increase. Following rapid elongation of the tentacles, contraction of longitudinally oriented muscle fibers in the stalks (which are arranged in separate bundles) shortens the tentacles. Just under the skin of the stalk, there are additional right- and left-handed arrays of helical muscle fibers that are responsible for torsion of the tentacles during the strike, but they will not be discussed further here (for more information, see Kier (1982)).

The transverse musculature of the tentacles shows ultrastructural specializations for high shortening velocity (Kier, 1985; Kier, 1991; Kier, 1996; Van Leeuwen and Kier, 1997; Kier and Thompson, 2003). Unlike much of the musculature of cephalopods, the transverse musculature is cross-striated with relatively short myosin filaments (mean for *Doryteuthis pealeii* of approximately 0.8 *μ*m) (Kier and Curtin, 2002). In contrast, the majority of the musculature of cephalopods, including the longitudinal muscle fibers of the tentacles, is obliquely striated, with relatively long myosin filaments (mean for transverse arm muscle of *Doryteuthis pealeii* of approximately 7.4 *μ*m) (Kier and Curtin, 2002; Thompson et al., 2022). In addition, analysis of the biochemistry of the myofilament lattice suggests that relatively little biochemical specialization has occurred in the cross-striated tentacle muscle fibers (Kier and Schachat, 1992; Kier and Schachat, 2008; Shaffer and Kier, 2012). Thus, specialization of these fibers for the rapid shortening that produces the explosive tentacular strike has involved a rearrangement and redimensioning of the myofilaments. There is no evidence for the types of biochemical specializations that have been observed previously in vertebrate muscle fiber types (Kier, 1991; Kier and Schachat, 1992; Kier and Schachat, 2008). The observed ultrastructural specializations would be expected to increase the shortening velocity of the tentacle fibers by an order of magnitude, compared with obliquely striated fibers from a serially homologous muscle mass in the arms. Measurements of the contractile properties of these two fiber types in *Doryteuthis pealeii* are consistent with this prediction; unloaded shortening velocities (*V*_max_) of the cross-striated tentacle fibers were greater than 17 lengths per second (*ℓ*s^−1^) (mean of approximately 15 *ℓ*s^−1^) while the *V*_max_ of the obliquely striated arm fibers was about 1.8 *ℓ*s^−1^ (mean of approximately 1.5 *ℓ*s^−1^) (Kier and Curtin, 2002).

We developed the first forward dynamic model that predicts the extension of the strike from muscle activation, sarcomere ultrastructural parameters and a simplified geometry of the tentacle (Van Leeuwen and Kier, 1997). The stalk was modeled as a longitudinal array of circular discs that interact mechanically. By assuming that the volume of each disc of the stalk is constant, the radial and longitudinal dimensions were coupled, essentially leading to a one-dimensional model. Although bending motions of the tentacles could not be simulated, the model is computationally highly efficient. Hence, this model could be used effectively to investigate the implications of different sarcomere geometries (in particular, myofilament lengths) on the tentacular extension speed. Inspired by this model, more complex finite element models have been constructed by adding dedicated routines describing basic muscle mechanics to commercially available finite element codes. These models (Johansson et al., 2000; Liang et al., 2006; Tang et al., 2009; Vavourakis et al., 2014) predict a very similar extension behavior to the model of Van Leeuwen and Kier (1997). The advantage of the finite element models is their ability to include a three dimensional (3D) spatial resolution while allowing the computation of complex 3D motions such as bending and twisting. The disadvantage, however, is their considerable computational load, especially when the fluid-structure interaction must be resolved. Other approaches attempt to circumvent the considerable computational load of the finite element models while still capturing the key mechanical features of highly deformable musculoskeletal systems. An example is the approach proposed by Zhang et al. (2019) that models the musculoskeletal system as an interacting set of heterogeneous active and passive Cosserat rods. The finite element and Cosserat rod methods both represent useful alternatives for modelling musculoskeletal systems and soft robotic arms. Ultimately, the choice of a particular model is determined by the type of scientific questions and the available computational resources.

We explored previously the implications of these ultrastructural specializations in the tentacle muscle fibers for the overall performance of the tentacles during the strike using this forward dynamic modelling approach (Van Leeuwen and Kier, 1997). This analysis suggests that the short myosin and actin filaments of the cross-striated tentacle fibers are necessary to produce the observed extension performance of the tentacles during the strike. Because we were primarily interested in the implications of the muscle-fiber ultrastructure for overall tentacle performance, we did not explore in detail how changes in the pattern of activation of the extensor muscle fibers along the stalk affect tentacle extension and assumed the same activation along the stalk, ignoring, for example, activation delays along the stalk. Activation delays along the tentacles have not yet been studied *in vivo* due to significant experimental difficulties. The motor input will also differ between different tentacle extensions, for instance because the inputs to the squid’s sensory systems will differ, and the sensory systems, nervous system, and muscle fibers are inherently noisy (see Faisal et al. (2008) for a review on the causes of, and solutions to, noise in the nervous system). Cephalopods have evolved a strikingly effective tentacular prey-capture mechanism (Messenger, 1977; Kier and Van Leeuwen, 1997), suggesting that they can suppress negative effects of stochasticity in the motor input of the tentacles on extension performance. Averaging at multiple linked architectural scales is a possible solution to counteract the stochasticity in the motor system (Faisal et al., 2008). In addition, the properties of the receiver (e.g., a muscle fiber) may be adjusted so as to minimize the effects of additive noise in its input signals. Muscle intrinsic properties may support the stability of motion systems and may reduce the requirements of the neurosensory control system (Nishikawa et al., 2007). Similarly, the non-linear mechanical properties of muscle and connective tissue may help to attenuate negative effects of noise in the neural input on the tentacular motor performance (see Discussion for details).

Rapid excitation-contraction coupling of the transverse and circular muscle fibers of the tentacular stalks is essential for the high performance of the tentacles during prey capture. Comparison of the tentacle fibers with the serially homologous transverse muscle fibers of the arms shows that the ratio of twitch force to peak tetanic force is 0.66 in the tentacle fibers and only 0.03 in the arm fibers (Kier and Curtin, 2002). In a recent study, whole-cell patch clamp recordings of dissociated fibers from the transverse muscle of the tentacles and the transverse muscle of the arms of *Doryteuthis* showed a 10-fold greater sodium conductance in the tentacle fibers compared with the arm fibers and, unlike the arm fibers, the tentacle fibers produce action potentials (Gilly et al., 2020). In addition, *in situ* hybridization with an antisense probe to the voltage-dependent sodium channel present in *Doryteuthis* showed conspicuous expression of sodium channel mRNA in the tentacle fibers and undetectable expression in the arm fibers (Gilly et al., 2020). The activation of the tentacle fibers appears to be an all-or-nothing type of electrical excitability that depends on action potentials based on sodium influx, similar to what has been observed in vertebrate skeletal muscle. Thus, a few closely spaced action potentials likely result in maximal activation of the transverse tentacle muscle. The slower movements of the arms are produced by a graded type of activation without action potentials, which is more typical of invertebrate muscle (Hoyle, 1969; Zachar, 1971).

The arms of cephalopods have inspired engineers to develop soft robotics arms for manipulation of objects (e.g., Margheri et al., 2012). The rapid extension mechanism of the tentacles has received less biomimetic attention than the arm-inspired gripping designs. The tentacles of squid and cuttlefish can extend much more than the arms, due to the unique cross-striated extensor muscles that generate the rapid extension and the specialized obliquely striated longitudinal and helical muscles that accommodate extensions and contractions of up to 80% as well as torsion. Our current simulations will provide insight for the future development of tentacle-inspired soft robotic rapid extension mechanisms.

In this paper, we use the previously developed forward-dynamic model (Van Leeuwen and Kier, 1997) to explore the effects of variation in activation of the extensor muscle fibers on the dynamics of the tentacular strike. In particular, we consider the effects of a delay in the activation of the extensor muscles along the stalk, from the base to the tip and *vice versa*, on the extension performance with metrics such as peak extension velocity. In addition, we examine the effects on the fluctuations of muscle power, elastic energy storage and release, and kinetic-energy variations along the tentacle. Because tentacular stalks slightly taper from proximal to distal, we investigate whether tapering affects the activation delay that maximizes the peak extension velocity. Furthermore, we explore the robustness of tentacle extension against noise in the onset of the activation input of the extensor muscles and examine whether motor-input averaging can improve the extension performance. This analysis is important not only for our understanding of the dynamics of the rapid tentacular strike and the sensitivity to variations in the motor input, but also for understanding the dynamics of movement in other muscular hydrostatic structures.

## 2 Materials and methods

### 2.1 Materials

High-speed films were made of squid (*Doryteuthis pealeii*; (LeSueur, 1821)) capturing shrimps as described by Kier and Van Leeuwen (1997). To quantify the forward body motion and tentacle extension, coordinates of landmarks on the body and tentacle were digitized, smoothed and differentiated using quintic splines in combination with the criterion of Generalized Cross Validation (Woltring, 1986) as explained by Kier and Van Leeuwen (1997). Results of these observations were previously used to test the tentacle extensions predicted by the model (Van Leeuwen and Kier, 1997). Here, we use the same model as the main tool for our analyses of the effects of changes in spatiotemporal activation of the transversely and circularly oriented muscles fibers (see Results).

### 2.2 Model approach

To study the effects of activation delays along the tentacle, we used a slightly modified version of the model of Van Leeuwen and Kier (1997). Here, we summarize the simplifying assumptions and the basic model structure, and outline the simulation procedures. Quantitative details of the model can be found in Van Leeuwen and Kier (1997) and the Supplementary Material S1, Section 2. An overview of the symbols and definitions that we used for the model and the statistical analysis of the model predictions is provided in Supplementary Table S1.

#### 2.2.1 Simplifying assumptions

**FIGURE 2:**
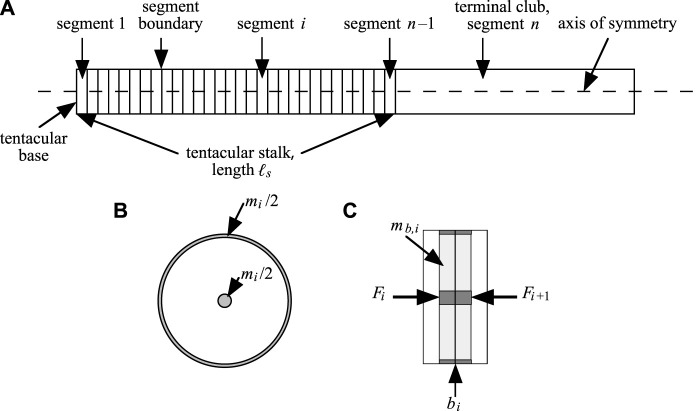
**(A)** Sketch of the model of Van Leeuwen and Kier (1997), as used in this paper. The tentacular stalk is divided in *n* −1 discs and a tentacular club of mass *m*_*club*_ is attached as segment *n*. **(B)** Transverse section of segment. In the model, the segmental mass is divided in two equal portions, one half concentrated in the center and the other concentrated as a ring at the segmental boundary (shaded areas). Both mass concentrations have identical longitudinal positions at all times. **(C)** Sagittal view of two neighboring segments. Longitudinally directed forces *F*_*i*_ and *F*_*i*+1_ (Supplementary Equation S24) on mass *m*_*b*,*i*_ (sum of darkly shaded mass concentrations; *m*_*b*,*i*_ = (*m*_*i*_ + *m*_*i*+1_)/2) for *i* ∈ [1,…, *n* −1] associated with boundary *b*_*i*_. After Van Leeuwen and Kier (1997).

Following Van Leeuwen and Kier (1997), we assume axial symmetry along the tentacle model. The tentacle is represented as a longitudinal array of disc-shaped segments, each with a circular cross-section in a transverse plane and a constant volume (Figure 2). Hence, any change in segment radius *r*_*i*_ is linked to a change in segment height *h*_*i*_ to ensure volume conservation. Along the tentacle, lumped masses occur at the segment boundaries. By assuming axial symmetry and incompressible material, simple relationships can be derived for the radial and longitudinal strain of each segment (see Supplementary Material S1, Section 2). At boundary *i*, half of segment mass *i* and half of segment mass *i* + 1 are assigned to the total boundary mass. Furthermore, half of the boundary mass was assumed to be concentrated in the center and the other half at the periphery. This radial mass division provides an accurate prediction of kinetic energy associated with the radial contraction of each segment (see Van Leeuwen and Kier, 1997). The segments along the stalk have equal volumes. Following Van Leeuwen and Kier (1997), we represented the club as one rigid segment with a considerably larger volume than that of the stalk segments.

In each stalk segment, the circularly arranged extensor muscles (CM, Figure 1) are assumed to have equal activation and strain, and exert the same tensile stress as the transverse muscle fibers. Together, they are called the extensor muscles. Only the extensor muscles are assumed to be active during extension of the stalk. The actions of the thin helically arranged muscle layers are ignored. Only frictional (retarding) and passive tensile forces of the longitudinal muscle fibers are included in the computations. Equal mechanical states of all the muscle fibers in one stalk segment preserves the axisymmetric condition.

The fluid pressure is assumed to be uniform in each stalk segment. Viscous forces in the external fluid and frictional forces on the tentacle are ignored. The short duration of the extension process (about 20–40 ms) is considered too brief for the development of a significant boundary layer. Van Leeuwen and Kier (1997) estimated a boundary layer thickness of only 0.173 mm towards the end of the elongation phase, about 2.3% of the tentacular diameter. They furthermore assumed a value of 8% of the tentacular mass for the effective added mass of water. Here we also implement these estimates. Finally, no interaction between tentacle and prey was assumed, which avoids difficult-to-predict complex movements.

#### 2.2.2 Summary of the model

Here we used the forward dynamics model developed by Van Leeuwen and Kier (1997) to compute the extension of the tentacle. The model inputs are the activation levels of the muscular segments of the stalk. The resulting extension computed by the model depends on the activation distribution, the geometry of the tentacle and sarcomeres, physiological parameters that define the dependence of the muscle-fiber force on the longitudinal strain and strain rate of the muscle fibers, and passive stiffness components. Quantitative details of the model can be found in Van Leeuwen and Kier (1997) and the Supplementary Material S1, Section 2.

**FIGURE 3:**
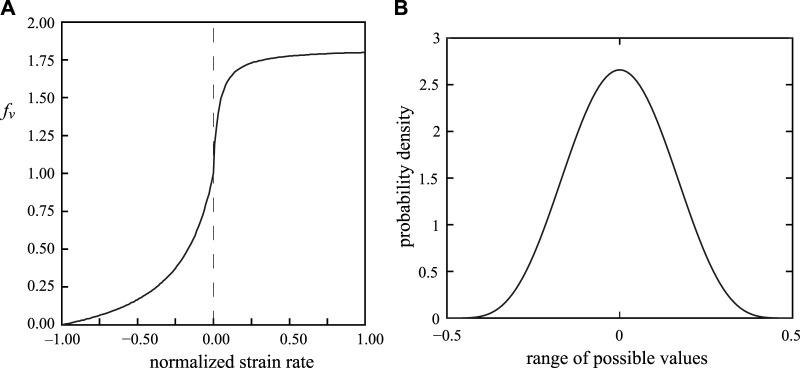
**(A)** Relationship between force *f*_*v*_ and normalized strain rate of a muscle fiber as used in the model. **(B)** Probability density function to add noise to the activation timing of the muscular segments of the stalk. The beta function with parameters *a* = *b* =5.8 was selected. The range of possible values was shifted by adding −0.5 to yield a left-right symmetrical function around zero. The values drawn randomly from the distribution were multiplied by either 10 or 20 to generate random delay values in the intervals [−5 ms 5 ms] and [−10 ms 10 ms].

Following Van Leeuwen and Kier (1997), we assumed that, in each segment, the nominal tensile stress in the extensor muscles (i.e., the tensile force per cross-sectional area of the initial relaxed state) depends on a passive component *σ*_*pas*_ plus the product of the maximum active isometric stress at optimum length *σ*_max_, the normalized active state *f*_*a*_, the velocity dependence function *f*_*v*_ (see Figure 3A), and the filamentary overlap function *f*_*ℓ*_ (Supplementary Material S1, Section 2: Supplementary Equation S1). The velocity dependence function approximates the observed force-velocity relationship of the muscle and the filamentary overlap function approximates the length-force relationship.

The activation of each muscular segment was described with an active state function *f*_*a*,*i*_ that starts from zero (i.e., the inactive state) and rises in sigmoidal fashion to one (the fully active state) (see Supplementary Equations S2–S4). Delays in the onset of the activation *t*_*d*,*i*_ among tentacular segments can be set in an arbitrary manner, where a zero delay is assigned to the most proximal segment. The activation along the tentacle forms essential input of the model, and the effects of its variation on the extension performance are the focus of this paper.

Following Van Leeuwen and Kier (1997), we assumed that

$$\sigma_{\max,i}=\sigma_{\max,ref}\left(\ell_{\mathrm{myo,i}}-\ell_{bz}\right)/\left(\ell_{\mathrm{myo,ref}}-\ell_{bz}\right),$$ (1)

where *ℓ*_*myo*_ and *ℓ*_*bz*_ are the lengths of the myosin filament and the central bare zone on the myosin filament (which lacks cross-bridges). Furthermore, *σ*_*max*,*ref*_ = 280 kPa and *ℓ*_*myo,ref*_ = 1.58 *μ*m represent values of a reference sarcomere. We assumed a fixed value of 0.14 *μ*m for *ℓ*_*bz*_.

The non-linear functions *f*_*v*_, which depends on the longitudinal strain rate of the muscle fiber, and *f*_*ℓ*_, which depends on the strain (and hence the overlap of the myosin and actin filaments in the sarcomere), and the passive component *σ*_*pas*_ are quantified in the Supplementary Material S1, Section 2.

The mathematical background and the details of the numerical solution of the model are provided in Van Leeuwen and Kier (1997) and the Supplementary Material S1, Section 2.

#### 2.2.3 Simulations

The model was implemented numerically in a computer program as described by Van Leeuwen and Kier (1997), but MATLAB code (Versions 2021b and 2022b, The MathWorks, Inc, Natick, MA USA) was used instead of Pascal. The predicted results with MATLAB were identical to those generated with the previous Pascal code for a selected combination of input parameters. Van Leeuwen and Kier (1997) studied only extensions without any delay of the activation along the tentacle.

All simulations started with an identical radius *r*_0_ and cross-sectional area *A*_*c*0_ along the tentacular stalk of with initial length *ℓ*_*s*0_, as well as the same initial cross-sectional area *A*_*cℓ*0_ = 0.15*A*_*c*0_ along the longitudinal muscles. All stalk segments were initialized with the same initial length *h*_0_. Kinetic energies were computed from the velocities of the boundary masses in the longitudinal and radial directions (see Van Leeuwen and Kier, 1997), while power was computed from the dot product of force and velocity.

**FIGURE 4:**
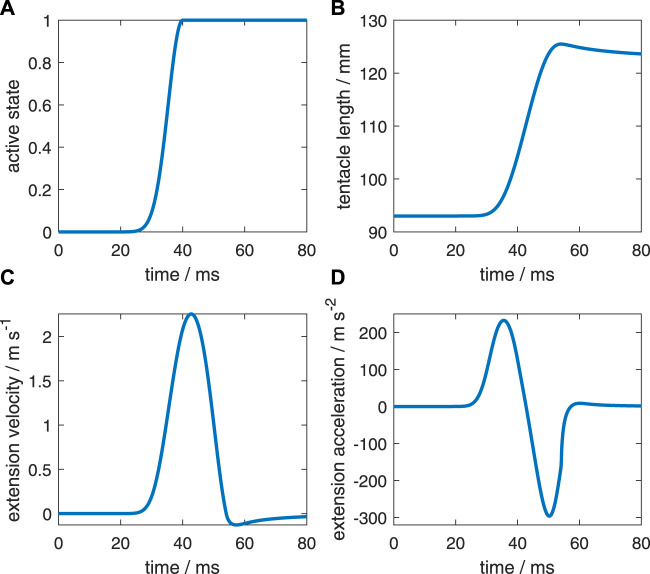
Characteristics of the reference simulation with the same active state along the muscular stalk of the tentacle (after Van Leeuwen and Kier (1997)). **(A)** Active state (model input). **(B)** Tentacle length. **(C)** Extension velocity of the stalk. **(D)** Extension acceleration of the stalk.

**FIGURE 5:**
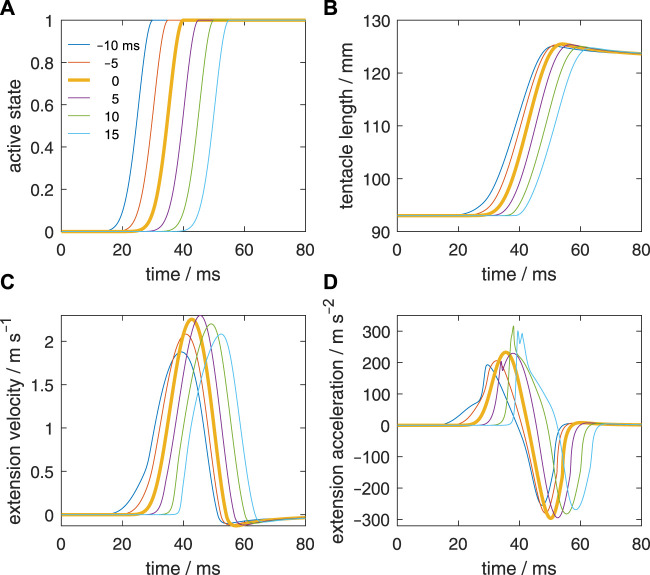
Effect of delays of the active state along the muscular stalk of the tentacle. **(A)** Six different prescribed delays in the active state. Each curve represents the input for a separate simulation. The activation delay of the tip of the stalk with respect to its base is indicated for each curve. A negative delay means that the tip is activated before the base of the stalk. It was assumed that the delay varies linearly along the stalk. **(B)** Changes in tentacle length for the six simulated delays along the stalk. **(C, D)** *Idem*, but now for the extension velocity and extension acceleration.

In addition to the reference simulation with a zero-activation delay (Figure 4), we simulated five delay examples along the stalk for a detailed comparison; each will be denoted by the activation delay Δ*t*_*d*_ between the most distal and most proximal stalk segments. A positive value means that the distal segment is delayed with respect to the proximal segment. The onset of all other segments was linearly interpolated between the values of the base and tip of the stalk. The prescribed delays were −10, −5, 0 (reference case), 5, 10, and 15 ms. Thus, in two cases, the activation started earlier at the tip than at the base of the stalk. The shape of the active state curves was kept identical along the stalk. Figure 5A depicts active state curves of the tip segment. The thick brown curve represents the reference simulation with a uniform activation along the stalk. To determine the Δ*t*_*d*_ that results in the highest peak-extension velocity of the tentacular stalk (denoted by Δ*t*_*d*,*vmax*_), we made a series of simulations between Δ*t*_*d*_ = −10 ms and Δ*t*_*d*_ = 15 ms at 0.5 ms intervals. To estimate Δ*t*_*d*,*vmax*_, we interpolated the collected peak velocities with a cubic spline function in MATLAB.

In the model, parameter *η* represents the fraction of the segmental volume occupied by extensor muscles. For the majority of our simulations, we use *η* = 0.7 for the most proximal segment, and *η* = 0.6 for the distal stalk segment. We assigned *η*-values to all the other segments by linear interpolation between the two extremes. We varied *η* along the stalk to account for a slight tapering of the stalk. The functional interpretation for this tapering is that distal segments have a lower mass in front of them that needs to be accelerated and hence do not need to be as forceful and powerful as the proximal segments. Muscle force is proportional to cross-sectional area and power is proportional to muscle volume. Ideally, both quantities should be adjusted along the stalk to the local mechanical requirements to optimize the spatiotemporal power delivery during the tentacular strike. Because tapering may affect the activation delay that maximizes extension performance, we also made simulations with *η* = 0.7 (i.e., *η* is constant along the stalk), *η* = 0.5, and *η* = 0.4 for the distal stalk segment, while keeping the proximal value constant at 0.7. We used this series of simulations to explore the effect of tapering on the activation delay that maximizes the peak extension speed.

We also examined the effects of random fluctuations in the activation onset of the individual segments along the tentacle. We used a symmetrical beta probability distribution with parameters *a* = *b* = 5.8 over the interval [−0.5 0.5] to assign random delay values to segments in addition to the deterministic delays specified above, which yields (approximately) a 0.5 probability that an assigned value is within [−0.1 0.1] (see Figure 3B). Random values drawn from the beta distribution were multiplied by a factor to convert them into milliseconds. We tested the extension performance using the intervals [−5 ms 5 ms] and [−10 ms 10 ms]. We used a beta function to select random values because this sets minimum and maximum values, in contrast to a normal distribution that extends from −*∞* to *∞*. The drawn random-delay values for each individual segment along the stalk were added to the deterministic delay values as described above. The difference in the activation onset of neighboring segments will increase on average due to the added noise. We simulated two independent series (with different initializations of the pseudo random number generator in MATLAB) of 50 extensions for each randomly affected activation-delay case with 50 segments along the stalk. The two series were merged to obtain 100 samples per noise affected delay group before statistical analysis.

A limitation in the segment-assigned random delay values is that it affects a whole segment as the current model uses the same activation over an entire segment. This worst-case scenario may actually be counteracted by a distributed averaging effect by the neuronal controller. Multiple motor-neurons will presumably contribute to the activation of the muscle fibers in any thin transverse slice through the stalk. This may result in an averaging effect on the overall activation that counteracts the stochastic activation effects on extension performance. Therefore, we made similar simulations (again, two times 50 simulations per case) with random additive activation noise, but for the segmental activation we averaged the active state resulting from ten randomly selected activation delays (while taking the prescribed deterministic delay into account). Effectively, we expect this to reduce the segmental out-of-phase activation effects on extension performance, but it also tends to increase the effective rise time of the active state for each segment.

For the four different added noise regimes and the six selected deterministic values of Δ*t*_*d*_, this resulted in 4 times 600 = 2,400 simulated tentacle extensions. For each noise regime, differences between the various distributions of the maximum extension speed were analyzed with a Bayesian estimation approach for two groups according to Kruschke (2013). A *t* location-scale distribution was used to model the probability distribution of the data:

$$p_{t}=\frac{\Gamma \left(\frac{\nu +1}{2}\right)}{\sigma \sqrt{\left(\nu -2/\nu \right)}\sqrt{\nu \pi }\Gamma \left(\frac{\nu }{2}\right)}{\left[\frac{\nu +{\left(\frac{x-\mu }{\sigma \sqrt{\left(\nu -2/\nu \right)}}\right)}^{2}}{\nu }\right]}^{-\left(\frac{\nu +1}{2}\right)}for\;\nu >2,$$ (2)

with gamma function Γ, location parameter *μ* (the ‘mean’), standard deviation parameter *σ*, and shape parameter *ν* (referred to as the normality parameter by Kruschke (2013)). The lower the value of *ν*, the higher the tails of the distribution are compared with those of the normal distribution. This property is exploited to account for outliers. A high value of *ν* (typically above 100) indicates that the samples are approximately normally distributed. For the two groups, *ν* was assigned the same value for every case. Hence, five parameters were used to describe the probability distributions of the two data sets: the mean and standard deviation for each group and a shared shape parameter: *μ*_1_, *μ*_2_, *σ*_1_, *σ*_2_, and *ν*. Bayesian inference was used to estimate the parameter values. This starts from an intentionally broad prior credibility distribution across parameter values followed by a credibility reallocation towards parameters in accordance with the data. For details of the approach, we refer to Kruschke (2013). We tested whether the means of the maximum extension velocity of the tentacle were different by checking 1) which percentage of the obtained credible values of the difference in the means fell in a region of practical equivalence (ROPE) of [−0.01 m s^−1^ 0.01 m s^−1^] and 2) which percentage of credible values for the effect size $E_{\mathrm{size}}=\left(\mu_{1}-\mu_{2}\right)/\sqrt{\left(\sigma_{1}^{2}+\sigma_{2}^{2}\right)/2}$ fell in a ROPE of [−0.1 0.1]. If values were below 5% for both cases (which is equivalent to the requirement that the 90% highest density interval of credible values falls outside the ROPE), it was accepted that the means of the maximum extension velocities were different from a functional perspective. In addition, we tested whether the means of the noise affected maximum extension velocity differed from the equivalent deterministic maximum extension velocity, *v*_max_, by examining whether the 90% highest density interval of the credible values fell outside a ROPE of [*v*_max_ − 0.01 *v*_max_ + 0.01] m s^−1^. The ±0.01m s^−1^ range for the velocity comparisons corresponds to a range of about ±0.5% around the peak velocity, which we regard as a negligible functional difference. A graphical illustration of the statistical procedure is provided in the Supplementary Material S1, Section 3.

## 3 Results

### 3.1 The reference extension

First, we will briefly describe the reference extension (Figure 4) that was simulated by Van Leeuwen and Kier (1997). Figure 4A shows the prescribed active state. The extensor muscles in all stalk segments were prescribed to have the same active state. Figures 4B–D show respectively the resulting length change of the muscular stalk, its extension velocity, and its extension acceleration. Once the maximum active state was reached, it was maintained until the end of the simulation. The predicted kinematics agreed well with an actual tentacle strike (for details, see Van Leeuwen and Kier (1997)).

### 3.2 Consequences of activation delays for the extension performance of the tentacle

Figure 5B shows the extension curves of the tentacle for all delay cases. The zero-delay case reaches the highest peak length, but the difference with the other cases is small, in particular for Δ*t*_*d*_ = −5 ms and Δ*t*_*d*_ = 5 ms. The steepest rise in the extension is generated in the Δ*t*_*d*_ = 5 ms case, closely followed by the zero-delay case. In all cases, the same tentacle length is generated at the end of the simulation (80 ms) due to identical activation at that stage and the nearly balanced stress of the extensor muscles and longitudinal elastic elements. Peak length is greater than the final length because of the rapid increase in momentum and kinetic energy during the extension phase, which make the longitudinal elastic elements stretch more than in the equilibrium case. The kinetic energy is converted into elastic energy and heat due to viscous losses.

Figure 5C shows the extension velocities of all delay cases. The highest extension velocity is reached by the Δ*t*_*d*_ = 5 ms case, closely followed by the zero-delay case and thereafter the Δ*t*_*d*_ = 10 ms case. The more negative is Δ*t*_*d*_ the lower is the peak extension speed. The peak extension velocity for Δ*t*_*d*_ = −5 ms and Δ*t*_*d*_ = 15 ms are nearly the same. Thus, negative delays are more detrimental for the extension performance than positive delays and a small positive delay may even slightly raise peak extension speed. Given that the tentacle extends in about 25 ms, it is striking that a delay of 15 ms still yields good performance. Thus, the model predicts that the tentacular muscle system is fairly robust to changes in activation delay along the stalk.

Figure 5D shows the extension accelerations for the various delay cases. For Δ*t*_*d*_ = 5, 10, and 15 ms, a steeper rise in the acceleration occurs than in the reference case. The two longest delays show the highest peak accelerations. A double positive peak acceleration occurs for Δ*t*_*d*_ = 5 ms and 15 ms.

**FIGURE 6:**
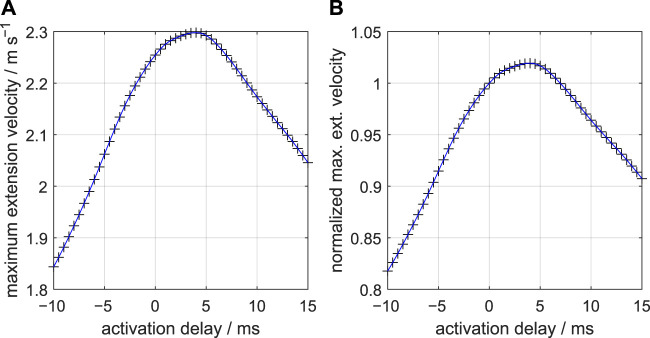
**(A)** Effect of delays of the active state along the muscular stalk of the tentacle on the peak-extension velocity of the tentacular stalk. Fifty-one simulations of single tentacle extensions were made at 0.5 ms steps. Symbols represent actual simulation output. The blue curve is a cubic spline through the data points. A delay of 4.4 ms yields the highest peak velocity. A nearly linear decrease occurs for delays beyond 7 ms. **(B)** Similar to **(A)** but now for peak velocity normalized with respect to the peak velocity at zero delay.

Figure 6 shows how the peak-extension velocity varies with the activation delay along the stalk. A relatively steep rise of the velocity peak occurs from a delay −10 ms to 0 ms. The maximum peak-extension velocity occurs for the delay Δ*t*_*d*,*vmax*_ of 4.4 ms, just above 2% higher than the maximum velocity of the zero-delay example. Peak-extension velocity decreases approximately linearly for delays greater than 7 ms. This decrease is less steep than the increase from Δ*t*_*d*_ = −10 ms to Δ*t*_*d*_ = −2 ms, showing that negative delays are particularly detrimental compared with positive delays. The optimal delay for peak extension velocity increased with a lower reduction of the volume fraction *η* of the extensor muscles along the stalk. The optimal values of Δ*t*_*d*_ were 3.85 ms, 4.2 ms, 4.4 ms, and 4.5 ms for the distal *η* values of 0.4, 0.5, 0.6, and 0.7, respectively (with *η* = 0.7 in the proximal stalk segment). The peak extension velocity of the stalk increased with a larger *η* along the stalk, which can be explained by the overall larger muscle volume that can power the extension.

### 3.3 Intra-segmental pressure variations in the tentacular stalk

A negative gradient in the intra-segmental fluid pressure along the stalk (i.e., with the highest pressure at the base, and the lowest pressure at the tip) provides a positive contribution to the forward acceleration of the material in the stalk and the terminal club (see Supplementary Equations S22, S24). The forward acceleration of the local stalk mass depends also on the gradient of the stresses and viscous forces in the longitudinal elements. The pressure variation along the stalk is the dominant component for this acceleration in the early phase when the strain and strain rates of the extensor muscle fibers, as well as the longitudinal strains and strain rates of the stalk segments are still relatively small. Around the maximum extension velocity, the viscous, retarding forces are highest, whereas the longitudinal elastic forces peak at maximum length of the stalk segments.

**FIGURE 7:**
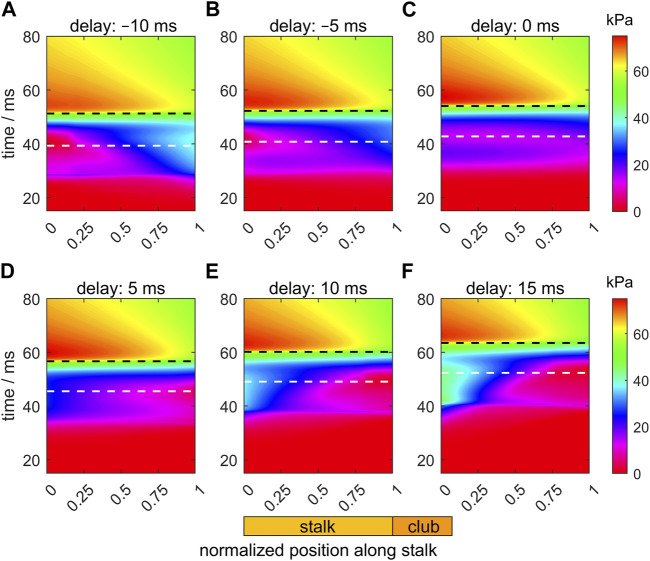
Panels **(A–F)** show the effects of delays of the active state (values provided in each panel title) along the muscular stalk of the tentacle on the variation in the intra-segmental pressure. Each panel shows a heat map of the pressure as a function of normalized location along the tentacle (abscissa; see also diagram of tentacle below panel **(E)**) and time (ordinate). In each panel, the times of maximum extension velocity and maximum tentacle length are shown by, respectively, the white and black dashed horizontal lines. The base and tip of the stalk have normalized positions of 0 and 1, respectively. The time along the ordinate of each panel is relative to the onset of the activity of the stalk for the reference extension with a zero delay (panel **(C)**).

Figure 7 shows heat maps of the intra-segmental pressure along the stalk and through time for the six selected values of the activation delay of the tip with respect to the base of the stalk. The white and black dashed horizontal lines in the panels indicate, respectively, the instants of the peak extension velocity and the maximum tentacle length. For the Δ*t*_*d*_ = −10 ms example (Figure 7A), the pressure in the tip region of the stalk rises first, leading to a positive pressure gradient, which is in contrast to the requirement for a fast overall forward acceleration. The positive pressure gradient is maintained considerably beyond the time of the maximum of the extension velocity. A local pressure minimum occurs at the base of the stalk shortly after the peak-extension velocity. A similar pressure pattern occurs for Δ*t*_*d*_ = −5 ms (Figure 7B), though with lower pressure at the tip of the stalk and a reduced positive pressure gradient before and considerably beyond the instant of maximum extension velocity. Overall, this improves the forward acceleration of the segments and the peak-extension velocity.

For the zero-delay case (Figure 7C), the pressure gradient along the tentacle is negative until just before peak-extension velocity when a slight pressure dip is formed at about 0.35 of the stalk length. At the base of the tentacle, the pressure first rises, then falls, followed by a final increase. Thus, the pressure gradient is again more favorable for forward acceleration of stalk mass, yet it is still not negative over the entire stalk during the duration of extension.

For Δ*t*_*d*_ = 5 ms (Figure 7D), the local pressure minimum around the peak-extension velocity along the stalk has shifted to the tip of the tentacle and the pressure gradient along the stalk is negative during the entire extension. For Δ*t*_*d*_ = 10 and 15 ms (Figures 7E,F), the pressure gradient becomes increasingly negative along the stalk, with a deeper local pressure minimum at the tip due to the late start of the activation. This deeper pressure minimum more than negates the positive effect of the pressure gradient on the acceleration and peak-extension speed because the distal segments extend less and are relatively ineffective in pushing the club forward.

### 3.4 Time-dependent specific muscle power in the tentacular stalk

**FIGURE 8:**
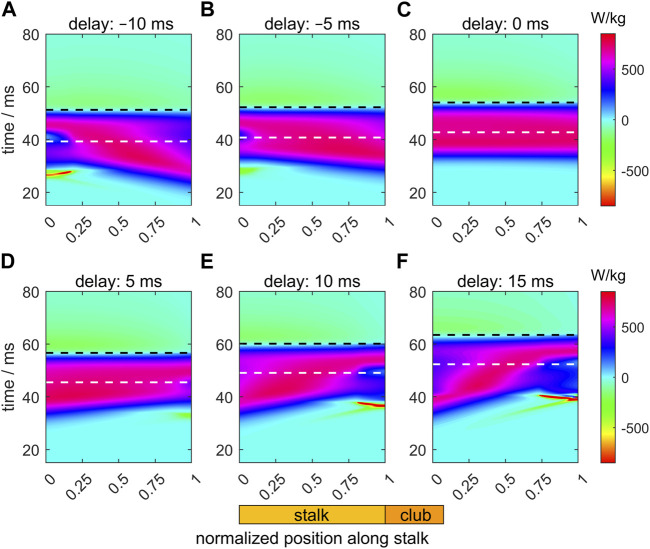
Panels **(A–F)** show the effects of delays of the active state (values provided in the title of each panel) along the muscular stalk of the tentacle on the variation in the specific mechanical muscle power (i.e., power per unit mass). Each panel shows a heat map of the power as a function of normalized location along the tentacle (abscissa) and time (ordinate). In each panel, the instants of the maximum extension velocity and maximum tentacle length are shown by, respectively, the white and black dashed horizontal lines. The time along the ordinate of each panel is relative to the onset of the activity of the stalk for the reference extension with a zero delay (panel **(C)**).

Figure 8 shows heat maps of the specific muscle power (i.e., the power per unit muscle mass in each segment) along the stalk during its extension for the six different activation delays. This takes account of the variation of the fraction occupied by muscle in each segment. The instants of maximum extension velocity and maximum tentacle length are depicted by, respectively, white and black dashed horizontal lines. For the zero-delay reference extension (Δ*t*_*d*_ = 0 ms; Figure 8C), the specific power along the entire stalk is largely synchronized and peaks shortly before the maximum extension velocity occurs. The approximate power synchrony is likely to contribute to the generated high extension velocity of the zero-delay example. Before the maximum tentacle length is reached, muscle power is positive over the entire stalk. After maximum stalk length, muscle power output becomes slightly negative along the tentacle because the extensor muscles are stretched during this phase as the stalk recoils towards its equilibrium length under maximal activation of the extensor muscles.

For the Δ*t*_*d*_ = 5 ms example (Figure 8D), with the highest extension speeds of all simulated delay examples, power synchrony is slightly reduced. A small negative ‘island’ occurs in the distal portion of the tentacle in the early extension phase. The proximal segments are activated and extend first, which compress (in combination with the inertia of the distal club) the still inactive (or slightly active) distal segments longitudinally while expanding them radially, thereby stretching the extensor muscles. Before peak-extension velocity, the power contribution of the proximal segments is larger than for the zero-delay case, while the contribution of the distal segments has decreased. A more negative (Figures 8A, B) or positive (Figures 8E, F) activation delay leads to a greater disruption of the power synchrony along the stalk and brief very negative power regions prior to the time of peak extension velocity. For Δ*t*_*d*_ < 0, the negative power regions occur in the proximal segment because the distal segments are activated first. For the positive delays, a reverse situation occurs. The greater out-of-phase muscle power output along the stalk leads to lower maximum extension speeds (see also Figures 5C; Figures 6).

### 3.5 Time-dependent specific muscle work in the tentacular stalk

**FIGURE 9:**
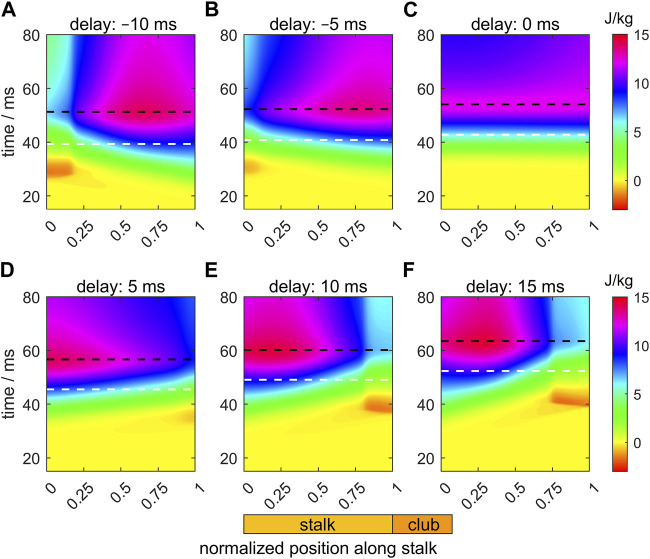
Panels **(A–F)** show the effects of delays of the active state (values provided in the title of each panel) along the muscular stalk of the tentacle on the variation in the specific mechanical muscle work (i.e., work per unit mass). Each panel shows a heat map of work as a function of normalized location along the tentacle (abscissa) and time (ordinate). In each panel, the instants of the maximum extension speed and maximum tentacle length are shown by, respectively, the white and black dashed horizontal lines. The time along the ordinate of each panel is relative to the onset of the activity of the stalk for the reference extension with a zero delay (panel **(C)**).

Figure 9 shows heat maps of the specific mechanical work output (i.e., the work per unit muscle mass) of the extensor muscles along the stalk during its extension for the six different activation delays. Mechanical work was computed by taking the time integral of the muscle specific power output. Times of maximum extension velocity and maximum stalk length are again indicated as horizontal white and black dashed lines. Prior to the maximum extension velocity, the reference example (Figure 9C) shows an almost perfect synchrony in the specific work output along the tentacle, as indicated by the approximately horizontal contours. Maximum work is done along the entire stalk at the instant of maximum extension. Thereafter some negative work is done by the extensor muscle due to stretching towards the final equilibrium length. At the tip and base of the stalk, negative work islands occur in the heat maps for the positive and negative delays, respectively (see Figures 9A, B for negative delays; Figures 9D–F for positive delays). The larger the deviation from the zero-delay case, the deeper these negative-work regions become. For Δ*t*_*d*_ = 5 ms, the proximal parts of the tentacle start to produce work earlier than during the reference extension. This more than compensates for the slight negative work phase at the distal end of the stalk, ultimately leading to a slightly higher maximum extension velocity. This favorable balance between positive and negative work along the stalk has disappeared for the other delay examples, which correlates with a decrease in maximum extension velocity. For all cases, maximum work is done by the stalk extensor muscles when the maximum length is reached.

### 3.6 Consequences of activation delays for the specific muscle, elastic, and kinetic power changes in the stalk

**FIGURE 10:**
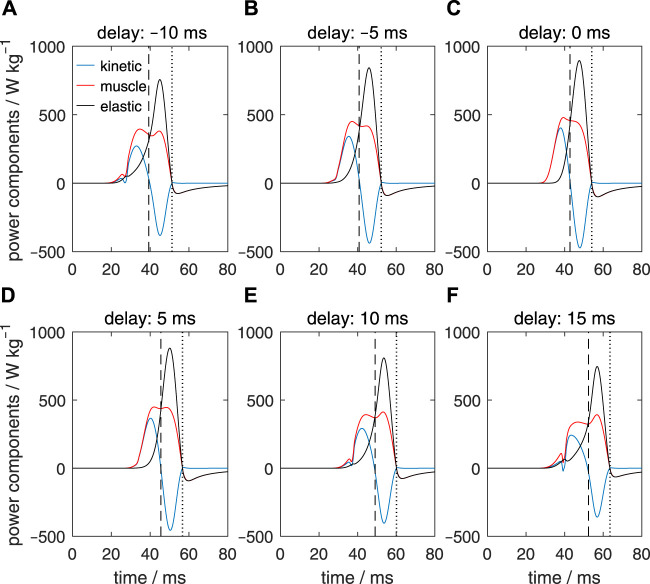
Panels **(A–F)** show the effects of delays of the active state along the muscular stalk of the tentacle on the variation in kinetic power (blue lines), muscle mechanical power (red lines), and elastic power (black lines). In each panel, the vertical dashed line shows the time of the maximum extension velocity of the tentacle, whereas the vertical dotted line indicates the instant the maximum tentacle length. Values of Δ*t*_*d*_ are shown above each panel. In contrast to the specific muscle power computed for Figure 8, specific muscle power was calculated here by taking the entire stalk mass into account.

We will now consider the changes in the specific mechanical power output (i.e., power per unit tissue mass) of the extensor muscles, the elastic power (here defined as positive for an increase in the stored elastic energy), and the kinetic power (the first time derivative of the kinetic energy) for the entire tentacle. Figure 10C shows the power fluctuations for the reference case (i.e., with a zero activation delay along the stalk). The vertical dashed line shows the instant of the maximum extension velocity of the tentacle, whereas the vertical dotted line indicates the time of the maximum tentacle length. The extensor muscles produce positive power over an interval of about 25 ms. This muscle power results initially in a rise in the kinetic power of the tentacle and later in a rise in the elastic power (shown positive when the material stores elastic energy). The elastic elements absorb muscle power as well as kinetic power. The drop in kinematic power is mainly due to a conversion into elastic energy and to a lesser extent to viscous losses (not shown). The kinetic power is zero at the time of the peak extension velocity. After peak extension has been reached, power in the extensor muscles is negative because the muscle fibers are extended while producing tension and thus absorb work. Figure 10D shows that the power fluctuations for Δ*t*_*d*_ = 5 ms are quite similar. For the most extreme delays (−10 ms and 15 ms; see Figures 10A, F), the positive muscle power phase has broadened and is reduced in magnitude compared with the reference and the Δ*t*_*d*_ = 5 ms cases. This change in muscular power delivery is responsible for the decrease in the peak extension velocity described above.

### 3.7 Effect of noise in the muscle-activation onset on the extension performance

**FIGURE 11:**
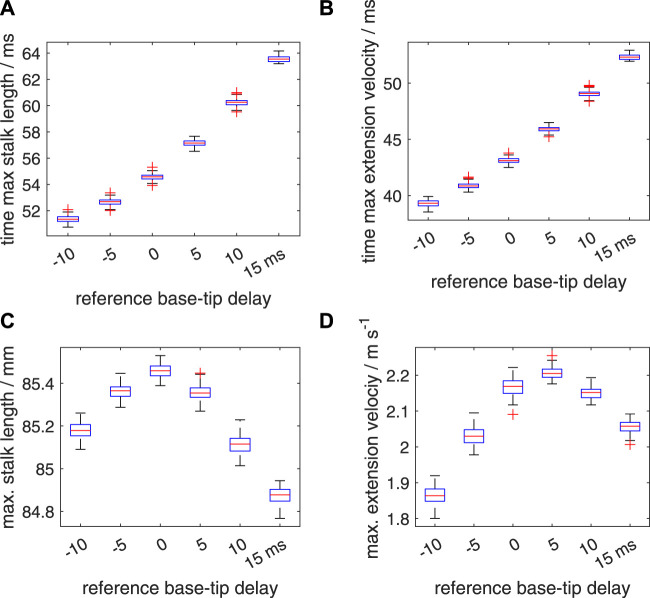
Effects of random variations in the timing of the activation on the extension performance of the tentacle. The tentacular stalk was divided into 50 segments and a single segment was assigned to the club. For each deterministic delay (Δ*t*_*d*_) between base and tip of the stalk (−10,−5,0,5,10, and 15 ms, respectively), 100 simulated extensions were made. For each simulation, an added random noise delay drawn from the interval [−5 ms 5 ms] was assigned to each segment along the stalk in addition to the segmental deterministic delay. Panels **(A–D)** show quartile-based box plots of time of maximum stalk length, time of maximum extension velocity, maximum stalk length, and maximum extension velocity, respectively, against Δ*t*_*d*_. Differences between the generated distributions of the maximum extension velocity were tested with a Bayesian estimation approach for two groups according to Kruschke (2013) (see main text for details).

So far, we discussed the effects of prescribed delays on the extension performance, while ignoring any random effects in the onset of the activation input. As described in section 2.2.3, we first simulated the effect of segmental random noise in the range of [−5 ms 5 ms], added to the prescribed deterministic delays along the stalk of respectively −10, −5, 0, 5, 10, and 15 ms. Box plots of the time to maximum tentacle length, time to maximum extension velocity, maximum stalk length and maximum extension velocity (Figure 11) show that the spreads around the medians of these performance metrics are rather small, with no or only a small number of outliers. Supplementary Table S2 shows the effect of added noise on the maximum extension velocity, *v*_max_. For the noise-modulated zero-delay case, a drop in the median and average *v*_max_ occurred of 3.8% and 3.9%, respectively, compared with the zero-noise value of 2.254 m s^−1^. Similarly, for Δ*t*_*d*_ = 5 ms, *v*_max_ = 2.292 m s^−1^, the median and mean peak velocities dropped both by 3.8%. Thus, the applied motor-input noise has on average only a small effect on the most critical performance indicator in these cases. For Δ*t*_*d*_ = 10 ms, *v*_max_ = 2.173 m s^−1^, the decreases in the median and mean velocities are only 1.0% and 1.1%, bringing it closer to the best performing Δ*t*_*d*_ = 5 ms delay case. Interestingly, for Δ*t*_*d*_ = −10 ms, *v*_max_ = 1.844 m s^−1^, with the lowest performance of the tested deterministic delays, the noise modulated median and mean values of *v*_max_ are actually increased by 1.0% and 1.1%, respectively. This was confirmed by the Bayesian analysis (Supplementary Figure S1) that showed that all credible values were greater than the deterministic value of *v*_max_ and outside the specified velocity ROPE (see section 2.2.3). Similarly for Δ*t*_*d*_ = 15 ms, *v*_max_ = 2.046 m s^−1^, the noise affected median and mean values are 0.6% and 0.5% above the deterministic case. Now, the Bayesian analysis (Supplementary Figure S1) showed that all credible values were greater than the deterministic *v*_max_, but 38% fell in the specified velocity ROPE (i.e., the most credible values were nevertheless outside the ROPE). Thus, on average, a narrow added-noise band improved the performance of the considered extreme negative and positive delay cases, but for Δ*t*_*d*_ = 15 ms this is a marginal gain. The Bayesian estimation illustrated in Supplementary Figure S2 shows that the noise perturbed Δ*t*_*d*_ = 5 ms case has a significantly greater mean extension velocity than the equivalent Δ*t*_*d*_ = 0 ms case.

For each of the 15 possible two-group comparisons of the six noise affected simulation sets, the Bayesian analysis showed that the difference between the credible values for the noise affected mean *v*_max_ fell outside the velocity ROPE, except for the combination of Δ*t*_*d*_ = 0 ms, Δ*t*_*d*_ = 10 ms with only 1.3% of the values in the velocity ROPE, and below the threshold of 5%. In addition, for all 15 effect sizes, 0% of the credible values fell in the effect size ROPE. Thus, mean peak extension speeds of the six simulation sets differed significantly from one another.

**FIGURE 12:**
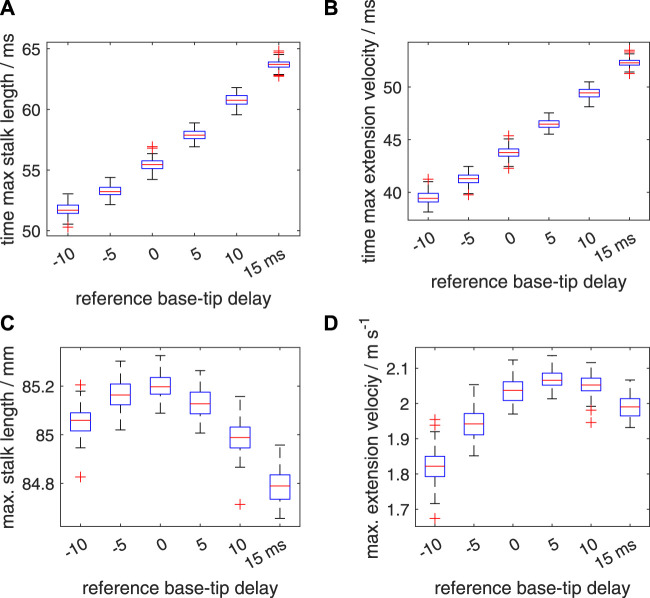
Similar plots as shown in Figure 11, but now for a random distribution interval of [−10 ms 10 ms] for the added noise in the activation delay. Panels **(A–D)** show quartile-based box plots of the maximum stalk length, time of maximum extension velocity, maximum stalk length, and maximum extension velocity, respectively, against Δ*t* *_d_*. Differences between the generated distributions of the maximum extension velocity were again analysed with a Bayesian estimation approach.

An added segmental noise in the timing of the motor input in the range of [−10 ms 10 ms] leads to higher standard deviations in the time to maximum stalk length, time to maximum extension velocity, maximum stalk length, and maximum extension velocity (Figure 12; Supplementary Table S2) than those for the [−5 ms 5 ms] range. For all considered delay cases, the noise-modulated median and mean peak-extension velocities are reduced compared to the deterministic *v*_max_, with all values below the corresponding values of the [−5 ms 5 ms] simulation set. Furthermore, the Δ*t*_*d*_ = 10 ms case is again more resistant to noise than the Δ*t*_*d*_ = 0 ms and Δ*t*_*d*_ = 5 ms cases, leading to an even slightly higher computed median and mean *v*_max_ than for the Δ*t*_*d*_ = 0 ms case, and only slightly below the values of the Δ*t*_*d*_ = 5 ms simulation. The Bayesian analysis of the 15 possible two-group comparisons showed that all mean peak velocities were different. For the combination of Δ*t*_*d*_ = 5 ms and Δ*t*_*d*_ = 10 ms, 10.79% of the credible values fell in the velocity ROPE, which is slightly above the limit of 5%, while only 0.14% fell in the effect size ROPE. The most credible values were outside both ROPEs. This indicates an average small performance difference with limited functional consequences.

**FIGURE 13:**
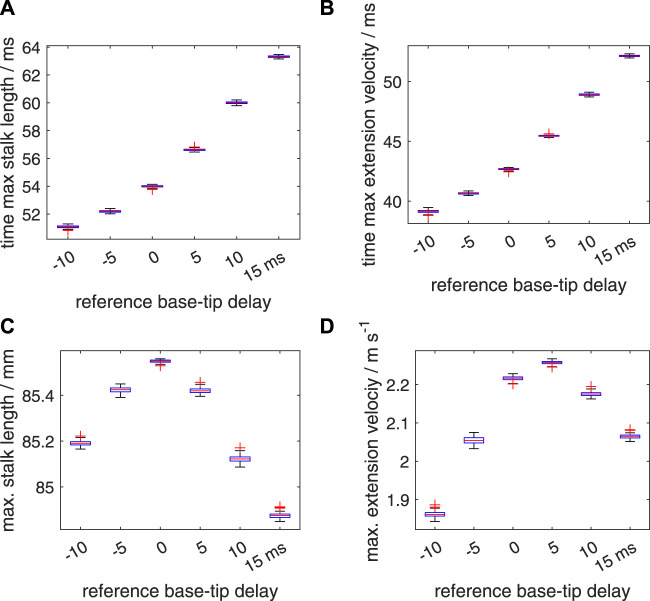
Similar plots as shown in Figure 11, again with a random distribution interval of [−5 ms 5 ms] for the added noise in the activation delay. Panels **(A–D)** show quartile-based box plots of the maximum stalk length, time of maximum extension velocity, maximum stalk length, and maximum extension velocity, respectively, against Δ*t* *_d_*. In contrast to Figure 11, ten random delays were drawn for each segment instead of one. These sampled random delays were averaged with an identical weight for each sample. Differences between the generated distributions of the maximum extension velocity were again analysed with a Bayesian estimation approach.

**FIGURE 14:**
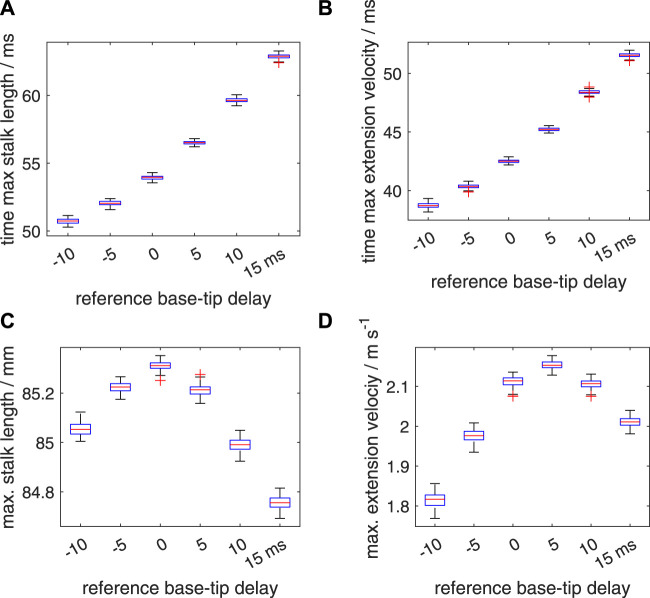
Similar plots as shown in Figure 12, with a random distribution interval of [−10 ms 10 ms] for the added noise in the activation delay. Panels **(A–D)** show quartile-based box plots of the maximum stalk length, time of maximum extension velocity, maximum stalk length, and maximum extension velocity, respectively, against Δ*t* *_d_*. In contrast to Figure 12, ten random delays were drawn instead of one. These sampled random delays were averaged with a identical weight for each sample. Differences between the generated distributions of the maximum extension velocity were again analysed with a Bayesian estimation approach.

So far, we have assigned only a single random delay value to each segment whereas in reality a large number of muscle fibers is present in any transverse section along the tentacular stalk, presumably innervated by multiple motor neurons. Thus, a single random delay is likely to represent a worst case scenario that deviates considerably from biological reality. To tackle this issue within the constraints of a single activation per segment in our dynamic model, we generated ten different random delay factors per segment. These were combined into a single activation input per segment as described in section 2.2.3. As expected, for both noise ranges, this leads to considerably smaller standard deviations in the computed distributions of the time to maximum stalk length, time to maximum extension velocity, maximum stalk length, and maximum extension velocity (Figures 13, 14; Supplementary Table S3) than for the simpler single noise value per segment approach. In addition, it shows a smaller reduction in the peak extension velocities compared with the deterministic values (Supplementary Table S3). The slight increase in the median and mean peak extension velocity compared with the deterministic extension velocity for Δ*t*_*d*_ = −10 ms and Δ*t*_*d*_ = 15 ms is again present for the noise interval of [−5 ms 5 ms]. This was confirmed by the Bayesian analyses that showed that 0% of the credible values fell in the velocity ROPE. For the Δ*t*_*d*_ = −5 ms and Δ*t*_*d*_ = 10 ms with noise interval [−5 ms 5 ms], all credible values were greater than the deterministic *v*_max_, but more than 99% of the values fell in the velocity ROPE, indicating only a marginal increase with negligible functional benefits. For the two noise ranges, the Bayesian analysis showed that all mean peak velocities were significantly different from one another with 0% of the credible values in the effect size ROPE. However, for the Δ*t*_*d*_ = 0 ms, Δ*t*_*d*_ = 10 ms combination with the [−10 10] ms noise interval, 97.5% fell in the velocity ROPE, indicating only a negligible functional difference and a greater robustness of the Δ*t*_*d*_ = 10 ms case against high levels of added noise in the onset of the activation input. Second, for the Δ*t*_*d*_ = −5 ms, Δ*t*_*d*_ = 15 ms combination with the [−5 ms 5 ms] noise interval, 30% of the credible values fell in the velocity ROPE, which indicates a relatively small average performance difference of these two cases.

## 4 Discussion

We have deliberately simplified our model by coupling local extension and contraction by the constant volume constraint, while not allowing bending and simplifying the fluid structure interaction. This allowed us previously (Van Leeuwen and Kier, 1997) to explore the functional relevance of the remarkably short sarcomeres of the extensor muscles, while it enabled us here to address the effects of activation delays along the stalk and additive noise in the activation onset.

The tentacular stalks of squid lack a rigid skeleton and thus support for the explosive elongation of the tentacles depends on the combination of active musculature and passive elastic properties of the muscle and connective tissues. We emphasize that when the musculature is in a relaxed state, the tentacular stalks exhibit extremely low stiffness in axial tension and compression and especially in bending. For instance, when handling an anesthetized animal, the tentacles hang limply and are fully elongated by their own weight simply by lifting the animal from the surface of the water. Given the low longitudinal stiffness of the tentacle, one might assume that for a rapid extension 1) it is essential that activation of the extensor musculature be simultaneous along the entire length of the tentacular stalk in order to avoid compression of inactive or weakly activated regions by highly active regions along the stalk and 2) that activation delays involving a substantial fraction of the time to peak extension velocity significantly degrade performance.

Our simulation results are striking because they suggest, in contrast to expectations, that the system is remarkably resistant to disruption caused by differences in the timing of activation along the length of the stalk. Indeed, the simulations predict, for the reference simulations, that a delay in activation of 4.4 ms at the tip of the stalk relative to the base generates the highest extension velocity during the strike, even higher than with simultaneous activation along the entire length of the stalk. Furthermore, even significantly large delays in activation cause relatively small decreases in performance. For instance, a delay in activation of 15 ms causes a decrease in maximum extension velocity of only 11% even though this delay represents 60% of the total duration of the tentacle strike. An important stabilizing factor is presumably the Hill-type non-linear relationship between muscle-fiber stress and contraction velocity (see Figure 3A). Lower activation tends to induce a lower contraction velocity with a positive feedback on force production that counteracts the unbalancing effect of activation differences between neighboring tentacular segments. Due to this positive effect of a slower contraction on force production, segments with a delay in activation can more easily catch up with segments that started earlier. In addition, distal stalk segments tend be loaded less than the proximal segments because a smaller mass in front of them needs to be accelerated. A slight activation delay along the stalk is therefore not harmful and may even increase the peak extension speed of the stalk because it reduces the phase delay between the *segmental* peak extension velocities along the stalk.

We expect the optimal activation delay to be reduced by tapering of the stalk from proximal to distal because tapering leads to a relatively higher load for the distal extensor muscles. We accounted partially for tapering by reducing linearly the volume fraction *η* of the extensor muscles along the stalk, from proximal to distal, while keeping the segmental masses constant. The simulation results show indeed a reduction in the optimal delay for peak extension velocity with a steeper decrease in *η* along the stalk (for distal values of *η* = 0.4, 0.5, 0.6, and 0.7, optimal delays were computed of approximately 3.85, 4.2, 4.4, and 4.5 ms). Larger maximum extension velocities were found with a lower reduction in *η* along the stalk. A slight reduction in segmental mass along the stalk while keeping the extensor muscle fraction constant may lead to slightly higher extension speeds. This, however, was not tested with simulations. Different loadings along the tentacular stalk could potentially be accommodated by varying the physiological and mechanical properties of the fibers of the extensor musculature. Kier and Curtin (2002) found no structural differences between the sarcomeres of the proximal and distal cross-striated muscle fibers of *D. pealeii*. In addition, Kier and Schachat (1992) and Kier and Schachat (2008) found that the myofilament biochemical properties of the extensor muscle and the obliquely striated muscles were similar. By slightly tapering the stalk and reducing the amount of extensor muscle from proximal to distal, its muscle fibers can be loaded in a nearly identical manner along the stalk. Hence, we do not expect that variation of the properties of the extensor muscle along the stalk would increase performance and, therefore, are likely unnecessary. During contraction of the tentacles, similar tensile forces are to be expected along the tentacular stalk. Hence, we expect only minor or no tapering in the longitudinal musculature along the stalk.

In our simulations, we used a Hill-type model approach in which activation is independent of strain and strain rate. Shue and Crago (1998) extended the Hill approach with a strain-history dependent coupling of activation and strain rate. Their model yielded a better fit with experimental data of the soleus muscle of the cat than the simpler Hill model or a model with length dependent activation. In the absence of experimental data, we do not know whether a similar length-history affected activation coupling occurs in the extensor muscles of the tentacular stalk. However, a compression induced lengthening of the extensor muscle in the distal segments due to a positive activation delay would enhance activation. This would tend to counteract the local compression, making the tentacle even more robust against tentacular activation delays than predicted here.

The peak instantaneous power per unit mass of the extensor muscles along the tentacle predicted here is just above 600W kg^−1^, which is beyond the mechanical limits measured for *in vitro* preparations of the tentacular extensor muscles by Kier and Curtin (2002). The cross-striated muscle fibers are very small (diameter 1 to 2 *μ*m) and are arranged in a meshwork with fibers that may be oriented (nearly) perpendicular to one another. So far, this complexity has prohibited measurements on isolated muscle fibers. The preparations used by Kier and Curtin (2002) contained multiple muscle fibers, including damaged fibers, and fibers that were not aligned with the applied force direction. Therefore, we expect that the *in vitro* measured peak muscle force and mass-specific power are lower than the *in vivo* abilities of single intact muscle fibers. In the model, we have used force and power limits that yield similar tentacle extensions as those recorded in *D. pealeii* (Kier and Van Leeuwen, 1997). The highest mass-specific instantaneous power for cross-striated muscle (average: 1121 W kg^−1^) (Askew and Marsh, 2001) has been measured for the pectoralis muscle of the blue-breasted quail (*Coturnix chinensis*), which is roughly twice as high as the peak power required for the tentacle extension of the squid.

**FIGURE 15:**
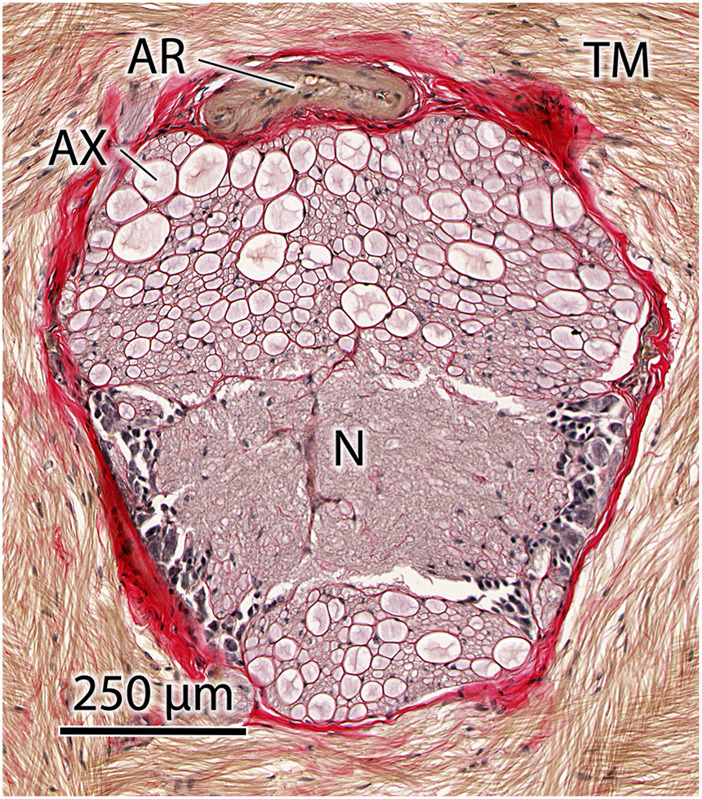
Transverse histological section of the axial nerve cord of the tentacle of *Doryteuthis pealeii* showing large axons (AX) in tracts above and below the central neuropil (N). The axial nerve cord is surrounded by the transverse muscle fibers (TM) of the tentacle, which are responsible for tentacle elongation. An artery (AR) is located in the connective tissue sheath (stained red) surrounding the axial nerve cord. Brightfield microscopy of 10 *μ*m thick paraffin section stained with Picro-Ponceau and hematoxylin.

As described above, the model predicts that a small delay in activation actually produces higher performance, in terms of extension velocity, than simultaneous activation of the extensor musculature. It is thus of interest to estimate the likely delay that occurs in the animal. We make the simplifying assumption that all motor neurons fire simultaneously from the same location. Although the conduction speed of the axons innervating the extensor musculature of the tentacle has not been measured, it is possible to estimate the conduction speed and thus the approximate delay in excitation at the terminal portion of the tentacle from morphological measurements. Figure 15 shows a histological transverse section of the axial nerve cord of the tentacular stalk of an adult *D. pealeii*. Tracts of large axons are visible above and below the central neuropil. Although the innervation of the transverse and circular muscle fibers in the stalk has not been studied in detail, it is reasonable to assume that the largest axons are responsible for stimulating the musculature in the terminal portion of the tentacular stalk, at the greatest distance from the base. Measurement of these axons shows them to be approximately 50–75 *μ*m in diameter. Since the material was fixed in formalin and embedded in paraffin, some shrinkage of the tissue is likely to have occurred. Dobrin (1996) measured the area of arteries cut in transverse section to decrease by 19%–25% if fixed in formalin and embedded in paraffin. This decrease in area results in a 10%–13% decrease in linear dimensions in the section plane. Thus, the actual diameter of the 50 *μ*m axons is likely 55.6 to 57.5 *μ*m and that of the 75 *μ*m axons is 83.3 to 86.2 *μ*m. We thus consider the axon diameters in the tentacle to be in the range 55–85 *μ*m. Using the relation derived by Hodgkin (1954) for unmyelinated axons, we calculate the conduction velocity to range from approximately 8 to 10 m s^−1^. Given the length of the tentacular stalk in adult *D. pealeii* of approximately 60 mm, this range of velocity results in a delay of 6 to 7.5 ms. Our simulations predict that an activation delay of 4.4 ms yields the highest extension velocity, a value that is remarkably close to the predicted delay based on independent morphological measurements and the assumption of synchronous activity of the soma of the motor neurons. In addition, we found that the extension performance is less susceptible to positive delays than to negative delays.

Our simulations show that additive noise in the activation onset of the extensor muscles slightly reduces, on average, the peak extension speed of the −5, 0, 5, and 10 ms delay cases, with the largest negative effects for the Δ*t*_*d*_ = 0 ms and Δ*t*_*d*_ = 5 ms cases which show the best extension performance. For the examined most extreme cases with, respectively, Δ*t*_*d*_ = −10 ms and Δ*t*_*d*_ = 15 ms, an additive noise window of [−5 ms 5 ms] had, on average, a small positive effect on the maximum extension speed of the tentacle. This did not occur for the [−10 ms 10 ms] window. We suggest that (close to) optimal delay cases are relatively susceptible to additive noise because it disturbs the near-optimal performance, whereas large deviations from the optimal activation delay may benefit from a small-amplitude additive noise because it may, on average, induce a shift towards a better delay condition.

Noise tends to increase with activation level in neural systems, which may affect the accuracy of motor control and motor control strategies, described as the “speed-accuracy trade-off” (Fitts, 1954; Harris and Wolpert, 1998). Unfortunately, we lack experimental data that show that the variation of the tentacle strike increases with, for instance, the maximum extension velocity. It may be that squid have evolved mechanisms to reduce noise in (near) maximum performance tentacle strikes, e.g., through the likely all-or-none excitation mechanism mentioned above and the presence of relatively large interneurons and motoneurons that reduce noise by averaging of input signals.

As expected, our model predicts that per-segment averaging of ten noise-affected activation inputs reduces the effect of noise on the extension performance, resulting in higher mean peak performances and a smaller standard deviation. We hypothesize that in the tentacles of squid, similar averaging effects are likely. Large neurons and axons are less susceptible to noise than small ones. The giant axons that activate the squid mantle muscles are an extreme example with many ion channels making the signal transduction nearly deterministic (Faisal et al., 2008). The large diameter of the motor axons in the tentacles of *Dorytheutis*, although one order of magnitude smaller than the giant axons, have nevertheless a considerable size that may help to reduce electrical noise. The all-or-none characteristic of the muscle fibers may be another factor in the suppression of noise effects in the motor output of the muscle fibers. The latter precludes a fine grading of the motor response, which seems an acceptable compromise for a high-speed capture mechanism. This begs the question of to what extent squid are able to vary the intensity of their tentacular strike. Jastrebsky et al. (2017) studied the turning behavior of the squid *Lolligunculla brevis* and showed a correlation between distance to prey and peak tentacle velocity. This implies that some modulation of the tentacle strike is possible in this species. To our knowledge, there is no study that has systematically addressed this question. Squid may also avoid using their tentacles for prey capture. The squid *Illex illecebrosus* captures slow or dead prey by jet-propelling themselves forward and catching the prey with their arms (Bradbury and Aldrich, 1969). A similar behavior was found in the squid *Sepiotheuthis lessoniana* hatchlings before the transverse muscles in the tentacles have differentiated from an obliquely striated to a cross-striated architecture (Kier, 1996).

*In vitro* measurements of conductance velocities of the motor axons may be a useful first step to test our hypotheses on the activation delays. Ideally, our theoretical predictions should be tested with activation measurements of live animals. This is, however, challenging because cephalopods are exceedingly dexterous and the behavior in question involves remarkably rapid movements with large deformations of the active tissues. Electrical recordings with wires from nerves and muscles are therefore not an option. This calls for the development of new remote recording technologies of electrical signals in free-ranging cephalopods.

We have simplified our modelling approach by ignoring bending. In reality, the tentacles easily curve by even relatively small applied external bending moments. This occurs for instance when the terminal clubs hit the prey during the strike. Our model cannot simulate such complex interactions. Several research teams (e.g., Johansson et al., 2000; Liang et al., 2006; Tang et al., 2009; Vavourakis et al., 2014) have produced finite element models with the aim to simulate the 3D motions of the arms and tentacles. Extensions of such models with appropriate contact mechanics, fluid structure interactions, and adequate controls may eventually allow accurate mechanical simulations of the predator-prey interactions of the tentacular strike.

Our simulations highlight the importance of activation-dependent mechanical properties of the muscles for reducing the accuracy requirements of the neural control system (in terms of activation delay and robustness against additive noise). We expect this finding to have general relevance for our understanding of the neuromuscular control of muscular hydrostats. In addition, our results may stimulate 1) the development of novel *in vivo* measurements of the spatiotemporal activation patterns of the tentacular musculature during prey capture and 2) the construction of highly deformable soft robots that simplify motion control by exploiting the intrinsic stabilizing properties of dedicated artificial muscles.

## Data Availability

The original contributions presented in the study are included in the article/Supplementary Material, further inquiries can be directed to the corresponding author.

## References

[B1] Askew G. N. Marsh R. L. (2001). The mechanical power output of the pectoralis muscle of blue-breasted quail (*Coturnix chinensis*): the *in vivo* length cycle and its implications for muscle performance. J. Exp. Biol. 204, 3587–3600. 10.1242/jeb.204.21.3587 11719526

[B2] Aubert X. (1956). Le couplage énergétique de la contraction musculaire. Brussels: Ph.D. thesis, Editions Arscia.

[B3] Bradbury H. E. Aldrich F. A. (1969). Observations on feeding of the squid *Illex illecebrosus illecebrosus* (lesueur, 1821) in captivity. Can. J. Zool. 47, 913–915. 10.1139/z69-148 5103494

[B4] Dobrin P. B. (1996). Effect of histologic preparation on the cross-sectional area of arterial rings. J. Surg. Res. 61, 413–415. 10.1006/jsre.1996.0138 8656617

[B5] Faisal A. A. Selen L. P. J. Wolpert D. M. (2008). Noise in the nervous system. Nat. Rev. Neurosci. 9, 292–303. 10.1038/nrn2258 18319728 PMC2631351

[B6] Fitts P. M. (1954). The information capacity of the human motor system in controlling the amplitude of movement. J. Exp. Psychol. 47, 381–391. 10.1037/h0055392 13174710

[B7] Gilly W. F. Renken C. Rosenthal J. Kier W. M. (2020). Specialization for rapid excitation in fast squid tentacle muscle involves action potentials absent in slow arm muscle. J. Exp. Biol. 223, jeb218081. 10.1242/jeb.218081 31900349

[B8] Harris C. M. Wolpert D. M. (1998). Signal-dependent noise determines motor planning. Nature 394, 780–784. 10.1038/29528 9723616

[B9] Hodgkin A. L. (1954). A note on conduction velocity. J. Physiol. 125, 221–224. 10.1113/jphysiol.1954.sp005152 13192767 PMC1365705

[B10] Hoyle G. (1969). Comparative aspects of muscle. Annu. Rev. Physiol. 31, 43–82. 10.1146/annurev.ph.31.030169.000355 4387945

[B11] Jastrebsky R. Bartol I. Krueger P. (2017). Turning performance of brief squid *Lolliguncula brevis* during attacks on shrimp and fish. J. Exp. Biol. 220, 908–919. 10.1242/jeb.144261 28167806

[B12] Johansson T. Meier P. Blickhan R. (2000). A finite-element model for the mechanical analysis of skeletal muscles. J. Theor. Biol. 206, 131–149. 10.1006/jtbi.2000.2109 10968943

[B13] Kier W. M. Curtin N. A. (2002). Fast muscle in squid (*Loligo pealei*): contractile properties of a specialized muscle fibre type. J. Exp. Biol. 205, 1907–1916. 10.1242/jeb.205.13.1907 12077167

[B14] Kier W. M. (1996). Muscle development in squid: ultrastructural differentiation of a specialized muscle fiber type. J. Morphol. 229, 271–288. 10.1002/(sici)1097-4687(199609)229:3<271::aid-jmor3>3.0.co;2-1 29852595

[B15] Kier W. M. Schachat F. H. (1992). Biochemical comparison of fast- and slow-contracting squid muscle. J. Exp. Biol. 168, 41–56. 10.1242/jeb.168.1.41 1640187

[B16] Kier W. M. Schachat F. H. (2008). Muscle specialization in the squid motor system. J. Exp. Biol. 211, 164–169. 10.1242/jeb.008144 18165243

[B17] Kier W. M. Smith K. K. (1985). Tongues, tentacles and trunks: the biomechanics of movement in muscular-hydrostats. J. Linn. Soc. Lond. Zool. 83, 307–324. 10.1111/j.1096-3642.1985.tb01178.x

[B18] Kier W. M. (1991). Squid cross-striated muscle: the evolution of a specialized muscle fiber type. Bull. Mar. Sci. 49, 389–403.

[B19] Kier W. M. (2012). The diversity of hydrostatic skeletons. J. Exp. Biol. 215, 1247–1257. 10.1242/jeb.056549 22442361

[B20] Kier W. M. (1982). The functional morphology of the musculature of squid (Loliginidae) arms and tentacles. J. Morphol. 172, 179–192. 10.1002/jmor.1051720205 30103569

[B21] Kier W. M. (1985). The musculature of squid arms and tentacles: ultrastructural evidence for functional differences. J. Morphol. 185, 223–239. 10.1002/jmor.1051850208 30011972

[B22] Kier W. M. Thompson J. T. (2003). Muscle arrangement, function and specialization in recent coleoids. Berl. Paläobiol. Abh. 3, 141–162.

[B23] Kier W. M. Van Leeuwen J. L. (1997). A kinematic analysis of tentacle extension in the squid *Loligo pealei* . J. Exp. Biol. 200, 41–53. 10.1242/jeb.200.1.41 9317299

[B24] Kruschke J. K. (2013). Bayesian estimation supersedes the t test. J. Exp. Psychol. General 142, 573–603. 10.1037/a0029146 22774788

[B25] Liang Y. McMeeking R. M. Evans A. G. (2006). A finite element simulation scheme for biological muscular hydrostats. J. Theor. Biol. 242, 142–150. 10.1016/j.jtbi.2006.02.008 16580021

[B26] Margheri L. Laschi C. Mazzolai B. (2012). Soft robotic arm inspired by the octopus: i. From biological functions to artificial requirements. Bioinspir. Biomim. 7, 025004. 10.1088/1748-3182/7/2/025004 22617132

[B27] Messenger J. B. (1977). Prey-capture and learning in the cuttlefish. Sepia (London: Academic Press.

[B28] Messenger J. B. (1968). The visual attack of the cuttlefish, *Sepia officinalis* . Anim. Behav. 16, 342–357. 10.1016/0003-3472(68)90020-1 5691850

[B29] Nishikawa K. Biewener A. A. Aerts P. Ahn A. N. Chiel H. J. Daley M. A. (2007). Neuromechanics: an integrative approach for understanding motor control. Integr. Comp. Biol. 47, 16–54. 10.1093/icb/icm024 21672819

[B30] Otten E. (1987). A myocybernetic model of the jaw system of the rat. J. Neurosci. Meth. 21, 287–302. 10.1016/0165-0270(87)90123-3 3682879

[B31] Shaffer J. F. Kier W. M. (2012). Muscular tissues of the squid *Doryteuthis pealeii* express identical myosin heavy chain isoforms: an alternative mechanism for tuning contractile speed. J. Exp. Biol. 215, 239–246. 10.1242/jeb.064055 22189767 PMC3244340

[B32] Shue G. Crago P. E. (1998). Muscle-tendon model with length history-dependent activation-velocity coupling. Ann. Biomed. Eng. 26, 369–380. 10.1114/1.93 9570220

[B33] Tang C. Y. Zhang G. Tsui C. P. (2009). A 3D skeletal muscle model coupled with active contraction of muscle fibres and hyperelastic behaviour. J. Biomech. 42, 865–872. 10.1016/j.jbiomech.2009.01.021 19264310

[B34] Thompson J. T. Taylor-Burt K. R. Kier W. M. (2022). One size does not fit all: diversity of length-force properties of obliquely striated muscles. J. Exp. Biol. 225, jeb244949. 10.1242/jeb.244949 PMC10658899 36633589

[B35] Van Leeuwen J. L. Kier W. M. (1997). Functional design of tentacles in squid: linking sarcomere ultrastructure to gross morphological dynamics. Phil. Trans. Roy. Soc. Lond. B 352, 551–571. 10.1098/rstb.1997.0038

[B36] Van Leeuwen J. L. (1991). Optimum power output and structural design of sarcomeres. J. Theor. Biol. 149, 229–256. 10.1016/s0022-5193(05)80279-6 2062094

[B37] Vavourakis V. Kazakidi A. Tsakiris D. P. Ekaterinaris J. A. (2014). A nonlinear dynamic finite element approach for simulating muscular hydrostats. Comput. Methods Biomech. Biomed. Engin. 17, 917–931. 10.1080/10255842.2012.723702 23025686

[B38] Woltring H. J. (1986). A fortran package for generalized, cross-validatory spline smoothing and differentiation. Adv. Engng Softw. 8, 104–113. 10.1016/0141-1195(86)90098-7

[B39] Zachar J. (1971). Electrogenesis and contractility in skeletal muscle cells. Baltimore, MD: Univ. Park Press.

[B40] Zhang X. Chan F. K. Parthasarathy T. Gazzola M. (2019). Modeling and simulation of complex dynamic musculoskeletal architectures. Nat. Commun. 10, 4825. 10.1038/s41467-019-12759-5 31645555 PMC6811595

