# Supplementary material for: Predicting the effects of spatiotemporal modifications of muscle activation on the tentacle extension in squid
Source: Front Bioeng Biotechnol. 2023 Oct 19;11:1193409. doi: 10.3389/fbioe.2023.1193409 (PMC10620692; doi:10.3389/fbioe.2023.1193409)
Supplement: Supplementary file 1 [file DataSheet2.PDF]

Supplementary material 2, belonging to Van Leeuwen & Kier (2023) Predicting the Effects of Spatiotemporal Modifications of Muscle Activation on the Tentacle Extension in Squid.
Underlying data for figures 11, 12, 13, and 14; Results os two-group Bayesian comparisons.

Results for 2 group comparison of the peak extension velocities of the tentacle using Bayesian estimation according to Kruschke (2013).
For each case, 2 times 50 = 100 simulations were made (n=100). Bayesian statistics was done using the 100 collected simulations per group.

l_max_1, l_max_2: maximum stalk length of, respectively, simulation series 1 and 2 (50 simulation samples per group for each deterministic delay case: -10, -5, 0, 5, 10 and 15 ms).
u_max_1, u_max_2: maximum extension velocity of, respectively, simulation group 1 and 2 (idem).
t_l_max_1, t_l_max_2: time of maximum stalk length of, respectively, simulation group 1 and 2 (idem).
t_u_max_1, t_u_max_2: time of maximum extension velocity of, respectively, simulation group 1 and 2 (idem).
delay_1: deterministic delay along the tentacle of the group 1.
delay_2: idem for group 2.
mu1_mean: mean of credible values for the mean peak extension velocity of group 1.
mu2_mean: idem for group two.
muDiff_mean: idem for the difference between the two groups.
mu1_median: median of credible values for the mean peak extension velocity of group 1.
mu2_median: idem for group 2
muDiff_median: idem for the difference between the two groups.
mu1_mode: mode of credible values for the mean peak extension velocity of group 1.
mu2_mode: idem for group 2.
muDiff_mode: idem for the difference between the two groups.
[mu1_HDIlow mu1_HDIhigh]: 90% highest density interval of the credible values for group 1; 5% of values are lower and 5% are higher.
[mu2_HDIlow mu2_HDIhigh]: 90% highest density interval of the credible values for group 2; 5% of values are lower and 5% are higher.
[muDiff_HDIlow muDiff_HDIhigh]: idem for the difference between the two groups.
Vdet1: peak deterministic extension velocity for group 1.
Vdet2: idem for group 1.
mu1_pcgtVdet1: percentage of credible values that is greater than Vdet1
mu2_pcgtVdet2: idem for group 2.
muDiff_pcgtZero: percentage of credible values for the difference that is greater than zero.
[mu1_ROPElow mu1_ROPEhigh]: velocity ROPE interval for mu1.
[mu2_ROPElow mu2_ROPEhigh]: velocity ROPE interval for mu2.
[muDiff_ROPElow muDiff_ROPEhigh]: velocity ROPE interval for the difference between mu1 and mu2.
mu1_pcInROPE: percentage of credible values that falls in the velocity ROPE of group 1.
mu2_pcInROPE: idem for group 1.
muDiff_pcInROPE: idem for the difference between groups.
nu_mean: mean of the credible values for nu (shape factor).

nu_median: idem for the median.
nu_mode: idem for the mode.
[nu_HDIlow nu_HDIhigh]: 90% highest density interval of the credible values for nu; 5% of values are lower and 5% are higher.
[nuLog10_HDIlow nuLog10_HDIhigh]: idem for the lof10 transformed values.
nuLog10_mean: mean of the log10 credible values for nu.
nuLog10_median: idem for the median.
nuLog10_mode: idem for the mode.
effSz_mean: mean of the credible values for the effect size of the velocity difference.
effSz_median: idem for the median.
effSz_mode: idem for the mode.
[effSz_HDIlow effSz_HDIhigh]: highest density interval for the 90% most credible values for the effect size.
effSz_pcgtZero: percentage of the credible values of the effect size that is greater than zero.
[effSz_ROPElow effSz_ROPEhigh]: ROPE for the effect size.
effSz_pcInROPE: percentage of credible values for the effect size that falls in the ROPE.
ROPE: region of practical equivalence.
sigma1_mean: mean of the credible values for the standard deviation for peak extension velocity of group 1.
sigma2_mean: idem for group 2.
sigma1_median: median of the credible values for the standard deviation for peak extension velocity of group 1.
sigma2_median: idem for group 2.
sigma1_mode: median of the credible values for the standard deviation for peak extension velocity of group 1.
sigma2_mode: idem for group 2.
[sigma1_HDIlow sigma1_HDIhigh]: highest density interval with 90% most credible values of the standard deviation for group 1.
[sigma2_HDIlow sigma2_HDIhigh]: idem for group 2.
sigmaDiff_mean: mean of the credible values for the difference in the standard deviations.
sigmaDiff_median: idem for the median.
sigmaDiff_mode: idem for the mode.
[sigmaDiff_HDIlow sigmaDiff_HDIhigh]: highest density interval with 90% most credible values for the difference in the standard deviations.
sigmaDiff_pcgtZero: percentage of the most credible values for the difference in the standard deviation that is greater than zero.

delays in ms; velocities in m/s; nu and effSz are dimensionless.

****
Results for noise_window for additive noise in activation used in simulations: [-5 ms 5 ms]; 1 random sample per segment.
****
Each row shows 50 simulation results for additive random noise; from top to bottom the reference deterministic delay was set to -10 -5, 0, 5, 10, 15 ms
t_l_max_1 =   [5.1903920e-02   5.1399920e-02   5.1375920e-02   5.1359920e-02
5.1327920e-02   5.1199920e-02   5.1463920e-02   5.1183920e-02   5.1583920e-02
5.1071920e-02   5.1015920e-02   5.1711920e-02   5.1743920e-02   5.1519920e-02
5.1031920e-02   5.1583920e-02   5.1535920e-02   5.1255920e-02   5.1687920e-02
5.0991920e-02   5.1551920e-02   5.1271920e-02   5.1367920e-02   5.1639920e-02

5.1151920e-02   5.1263920e-02   5.1511920e-02   5.1215920e-02   5.0887920e-02
5.1023920e-02   5.1247920e-02   5.1023920e-02   5.1463920e-02   5.1223920e-02
5.1631920e-02   5.1247920e-02   5.1479920e-02   5.0775920e-02   5.1655920e-02
5.0991920e-02   5.0887920e-02   5.1479920e-02   5.1487920e-02   5.1191920e-02
5.1311920e-02   5.1735920e-02   5.1063920e-02   5.1903920e-02   5.1311920e-02
5.1903920e-02;
5.2695920e-02   5.2471920e-02   5.2823920e-02   5.3087920e-02   5.2615920e-02
5.2647920e-02   5.2383920e-02   5.2727920e-02   5.3127920e-02   5.2735920e-02
5.2559920e-02   5.2719920e-02   5.2559920e-02   5.2303920e-02   5.3087920e-02
5.2487920e-02   5.2727920e-02   5.2703920e-02   5.2439920e-02   5.2703920e-02
5.2207920e-02   5.2831920e-02   5.3183920e-02   5.2911920e-02   5.2463920e-02
5.2407920e-02   5.2583920e-02   5.2815920e-02   5.2607920e-02   5.2951920e-02
5.3167920e-02   5.2919920e-02   5.2775920e-02   5.2847920e-02   5.2671920e-02
5.2815920e-02   5.2903920e-02   5.2655920e-02   5.2543920e-02   5.3023920e-02
5.2463920e-02   5.2575920e-02   5.2759920e-02   5.2695920e-02   5.2671920e-02
5.2119920e-02   5.2479920e-02   5.2447920e-02   5.2775920e-02   5.2807920e-02;
5.4335920e-02   5.4567920e-02   5.4887920e-02   5.4847920e-02   5.5015920e-02
5.4647920e-02   5.4559920e-02   5.4799920e-02   5.4319920e-02   5.4783920e-02
5.4615920e-02   5.4327920e-02   5.4671920e-02   5.4687920e-02   5.4607920e-02
5.4655920e-02   5.4575920e-02   5.4551920e-02   5.4391920e-02   5.4799920e-02
5.4479920e-02   5.4655920e-02   5.4495920e-02   5.4135920e-02   5.4863920e-02
5.4863920e-02   5.4471920e-02   5.4807920e-02   5.4359920e-02   5.5047920e-02
5.4175920e-02   5.4255920e-02   5.4071920e-02   5.4855920e-02   5.4407920e-02
5.4583920e-02   5.4575920e-02   5.4695920e-02   5.4903920e-02   5.4967920e-02
5.4191920e-02   5.4567920e-02   5.4495920e-02   5.4991920e-02   5.4535920e-02
5.4615920e-02   5.4343920e-02   5.4503920e-02   5.4743920e-02   5.4519920e-02;
5.6927920e-02   5.6711920e-02   5.7351920e-02   5.6903920e-02   5.7223920e-02
5.7463920e-02   5.7303920e-02   5.6807920e-02   5.7023920e-02   5.7287920e-02
5.6959920e-02   5.6983920e-02   5.6919920e-02   5.7351920e-02   5.7071920e-02
5.7311920e-02   5.7183920e-02   5.7151920e-02   5.7343920e-02   5.6951920e-02
5.7559920e-02   5.7135920e-02   5.7207920e-02   5.7335920e-02   5.6519920e-02
5.7087920e-02   5.7335920e-02   5.7023920e-02   5.7007920e-02   5.7423920e-02
5.7167920e-02   5.7335920e-02   5.7071920e-02   5.7231920e-02   5.7079920e-02
5.7135920e-02   5.7007920e-02   5.7183920e-02   5.6999920e-02   5.7223920e-02
5.6983920e-02   5.6647920e-02   5.7183920e-02   5.6983920e-02   5.6791920e-02
5.7063920e-02   5.7167920e-02   5.7279920e-02   5.7199920e-02   5.7511920e-02;
6.0271920e-02   6.0327920e-02   6.0383920e-02   5.9519920e-02   6.0207920e-02
6.0471920e-02   6.0351920e-02   6.0199920e-02   6.0055920e-02   5.9943920e-02
6.0487920e-02   5.9919920e-02   6.0215920e-02   6.0263920e-02   6.0015920e-02
6.0783920e-02   6.0231920e-02   6.0207920e-02   6.0143920e-02   6.0063920e-02
6.0223920e-02   6.0775920e-02   6.0895920e-02   5.9991920e-02   6.0151920e-02
6.0199920e-02   6.0791920e-02   6.0351920e-02   6.0135920e-02   5.9919920e-02
6.0087920e-02   5.9871920e-02   6.0151920e-02   6.0351920e-02   5.9799920e-02
6.0855920e-02   6.0351920e-02   6.0263920e-02   5.9903920e-02   6.0207920e-02
6.0415920e-02   6.0351920e-02   6.0295920e-02   6.0399920e-02   6.0791920e-02
6.0375920e-02   6.0687920e-02   6.0367920e-02   6.0383920e-02   6.0311920e-02;
6.3759920e-02   6.3359920e-02   6.3519920e-02   6.3479920e-02   6.3735920e-02
6.3335920e-02   6.3191920e-02   6.3335920e-02   6.3263920e-02   6.3231920e-02
6.3223920e-02   6.3287920e-02   6.3775920e-02   6.3639920e-02   6.3567920e-02
6.3855920e-02   6.3303920e-02   6.3423920e-02   6.3311920e-02   6.4007920e-02
6.3647920e-02   6.3839920e-02   6.3751920e-02   6.3719920e-02   6.3735920e-02
6.3567920e-02   6.3407920e-02   6.3823920e-02   6.3695920e-02   6.3551920e-02
6.3559920e-02   6.3615920e-02   6.3751920e-02   6.3639920e-02   6.3351920e-02
6.3775920e-02   6.4087920e-02   6.3591920e-02   6.3703920e-02   6.3471920e-02

```
6.3271920e-02   6.3719920e-02   6.3239920e-02   6.3727920e-02   6.3495920e-02
6.3375920e-02   6.3455920e-02   6.3287920e-02   6.3247920e-02   6.3703920e-02];
t_u_max_1 =       [3.9567920e-02   3.9151920e-02   3.9575920e-02   3.9631920e-02
 3.9399920e-02   3.9239920e-02   3.9151920e-02   3.8951920e-02   3.9463920e-02
3.9023920e-02   3.8575920e-02   3.9735920e-02   3.9767920e-02   3.9239920e-02
3.9111920e-02   3.9527920e-02   3.9223920e-02   3.9375920e-02   3.9711920e-02
3.8975920e-02   3.9447920e-02   3.8935920e-02   3.9351920e-02   3.9575920e-02
3.9399920e-02   3.9439920e-02   3.9231920e-02   3.9343920e-02   3.8535920e-02
3.8935920e-02   3.9175920e-02   3.9311920e-02   3.9567920e-02   3.9407920e-02
3.9551920e-02   3.9407920e-02   3.9271920e-02   3.8607920e-02   3.9431920e-02
3.8743920e-02   3.8783920e-02   3.9455920e-02   3.9615920e-02   3.9215920e-02
3.9527920e-02   3.9471920e-02   3.9175920e-02   3.9743920e-02   3.9223920e-02
3.9727920e-02;
   4.1031920e-02   4.0559920e-02   4.1103920e-02   4.1543920e-02   4.0703920e-02
  4.0735920e-02   4.0719920e-02   4.0847920e-02   4.1263920e-02   4.0863920e-02
 4.0623920e-02   4.0999920e-02   4.0975920e-02   4.0607920e-02   4.1071920e-02
4.0855920e-02   4.0743920e-02   4.0759920e-02   4.0583920e-02   4.0927920e-02
4.0303920e-02   4.0799920e-02   4.1287920e-02   4.1031920e-02   4.0751920e-02
4.0623920e-02   4.0607920e-02   4.0959920e-02   4.0799920e-02   4.1207920e-02
4.1255920e-02   4.1063920e-02   4.0951920e-02   4.0999920e-02   4.0951920e-02
4.0871920e-02   4.1287920e-02   4.0799920e-02   4.0863920e-02   4.1071920e-02
4.0543920e-02   4.0639920e-02   4.0895920e-02   4.0975920e-02   4.0751920e-02
4.0375920e-02   4.0975920e-02   4.0519920e-02   4.1111920e-02   4.0959920e-02;
   4.2959920e-02   4.3103920e-02   4.3399920e-02   4.3431920e-02   4.3391920e-02
  4.3007920e-02   4.3199920e-02   4.3319920e-02   4.2927920e-02   4.3295920e-02
 4.3159920e-02   4.2919920e-02   4.3191920e-02   4.3135920e-02   4.3199920e-02
4.3063920e-02   4.3151920e-02   4.3231920e-02   4.3103920e-02   4.3303920e-02
4.3039920e-02   4.3111920e-02   4.3079920e-02   4.2671920e-02   4.3455920e-02
4.3359920e-02   4.3023920e-02   4.3383920e-02   4.2999920e-02   4.3615920e-02
4.2823920e-02   4.2919920e-02   4.2735920e-02   4.3439920e-02   4.2871920e-02
4.3087920e-02   4.2959920e-02   4.3207920e-02   4.3455920e-02   4.3431920e-02
4.2823920e-02   4.3143920e-02   4.3055920e-02   4.3399920e-02   4.2975920e-02
4.3231920e-02   4.3031920e-02   4.3079920e-02   4.3063920e-02   4.3151920e-02;
   4.5727920e-02   4.5527920e-02   4.6015920e-02   4.5719920e-02   4.5967920e-02
  4.6271920e-02   4.6095920e-02   4.5551920e-02   4.5847920e-02   4.6063920e-02
 4.5679920e-02   4.5751920e-02   4.5671920e-02   4.6167920e-02   4.5911920e-02
4.6007920e-02   4.5967920e-02   4.5983920e-02   4.6103920e-02   4.5695920e-02
4.6319920e-02   4.6007920e-02   4.6055920e-02   4.6079920e-02   4.5223920e-02
4.5943920e-02   4.6039920e-02   4.5871920e-02   4.5783920e-02   4.6095920e-02
4.5879920e-02   4.5983920e-02   4.5799920e-02   4.5935920e-02   4.5807920e-02
4.5927920e-02   4.5727920e-02   4.5935920e-02   4.5743920e-02   4.6055920e-02
4.5679920e-02   4.5383920e-02   4.5927920e-02   4.5823920e-02   4.5583920e-02
4.5831920e-02   4.5919920e-02   4.6007920e-02   4.5999920e-02   4.6303920e-02;
   4.9127920e-02   4.9127920e-02   4.9167920e-02   4.8383920e-02   4.8991920e-02
  4.9167920e-02   4.9151920e-02   4.9079920e-02   4.8919920e-02   4.8799920e-02
 4.9335920e-02   4.8775920e-02   4.9095920e-02   4.9079920e-02   4.8815920e-02
4.9623920e-02   4.9063920e-02   4.9023920e-02   4.9031920e-02   4.8815920e-02
4.9047920e-02   4.9615920e-02   4.9711920e-02   4.8791920e-02   4.9007920e-02
4.8887920e-02   4.9631920e-02   4.9175920e-02   4.8951920e-02   4.8727920e-02
4.8903920e-02   4.8727920e-02   4.8991920e-02   4.9215920e-02   4.8543920e-02
4.9727920e-02   4.9167920e-02   4.9095920e-02   4.8703920e-02   4.8935920e-02
4.9207920e-02   4.9151920e-02   4.9087920e-02   4.9199920e-02   4.9615920e-02
4.9135920e-02   4.9527920e-02   4.9191920e-02   4.9167920e-02   4.9143920e-02;
   5.2551920e-02   5.2103920e-02   5.2303920e-02   5.2239920e-02   5.2527920e-02
```

5.2119920e-02   5.1983920e-02   5.2071920e-02   5.1983920e-02   5.1975920e-02
5.2039920e-02   5.2063920e-02   5.2559920e-02   5.2367920e-02   5.2351920e-02
5.2647920e-02   5.2111920e-02   5.2151920e-02   5.2079920e-02   5.2791920e-02
5.2423920e-02   5.2631920e-02   5.2559920e-02   5.2511920e-02   5.2543920e-02
5.2335920e-02   5.2143920e-02   5.2615920e-02   5.2383920e-02   5.2287920e-02
5.2319920e-02   5.2391920e-02   5.2527920e-02   5.2375920e-02   5.2119920e-02
5.2527920e-02   5.2855920e-02   5.2391920e-02   5.2463920e-02   5.2255920e-02
5.1999920e-02   5.2503920e-02   5.2015920e-02   5.2495920e-02   5.2231920e-02
5.2159920e-02   5.2271920e-02   5.2079920e-02   5.2031920e-02   5.2495920e-02];
l_max_1 =   [8.5152105e-02   8.5131086e-02   8.5199282e-02   8.5224823e-02
8.5181909e-02   8.5224324e-02   8.5116563e-02   8.5194380e-02   8.5145857e-02
8.5210483e-02   8.5131971e-02   8.5189566e-02   8.5165811e-02   8.5179393e-02
8.5235104e-02   8.5175471e-02   8.5165747e-02   8.5151376e-02   8.5186026e-02
8.5196477e-02   8.5182817e-02   8.5145630e-02   8.5208236e-02   8.5169748e-02
8.5177587e-02   8.5162266e-02   8.5144765e-02   8.5169383e-02   8.5164415e-02
8.5175522e-02   8.5210209e-02   8.5260494e-02   8.5201802e-02   8.5242413e-02
8.5133653e-02   8.5194662e-02   8.5175486e-02   8.5220920e-02   8.5148428e-02
8.5200792e-02   8.5236628e-02   8.5183692e-02   8.5228670e-02   8.5188296e-02
8.5252562e-02   8.5141630e-02   8.5243808e-02   8.5119078e-02   8.5147287e-02
8.5097772e-02;
8.5381545e-02   8.5367240e-02   8.5370846e-02   8.5347479e-02   8.5323134e-02
8.5330759e-02   8.5413171e-02   8.5378217e-02   8.5320760e-02   8.5364109e-02
8.5367814e-02   8.5378752e-02   8.5403978e-02   8.5375602e-02   8.5357238e-02
8.5385626e-02   8.5363843e-02   8.5319235e-02   8.5356358e-02   8.5351556e-02
8.5373392e-02   8.5320660e-02   8.5313839e-02   8.5349342e-02   8.5392046e-02
8.5379802e-02   8.5314759e-02   8.5352528e-02   8.5356771e-02   8.5372119e-02
8.5334374e-02   8.5335860e-02   8.5374319e-02   8.5343141e-02   8.5394664e-02
8.5377252e-02   8.5374698e-02   8.5340630e-02   8.5350387e-02   8.5334097e-02
8.5307456e-02   8.5329882e-02   8.5336353e-02   8.5364754e-02   8.5374846e-02
8.5395810e-02   8.5427936e-02   8.5368600e-02   8.5345676e-02   8.5306011e-02;
8.5465483e-02   8.5510313e-02   8.5418692e-02   8.5464939e-02   8.5426750e-02
8.5419483e-02   8.5458920e-02   8.5396079e-02   8.5505710e-02   8.5402200e-02
8.5472421e-02   8.5487764e-02   8.5443998e-02   8.5453312e-02   8.5504763e-02
8.5412317e-02   8.5486702e-02   8.5469253e-02   8.5444246e-02   8.5431909e-02
8.5497441e-02   8.5456982e-02   8.5484363e-02   8.5462456e-02   8.5415375e-02
8.5464285e-02   8.5479071e-02   8.5467286e-02   8.5504548e-02   8.5400612e-02
8.5487504e-02   8.5488655e-02   8.5479543e-02   8.5431792e-02   8.5445068e-02
8.5423754e-02   8.5412149e-02   8.5477201e-02   8.5455589e-02   8.5446692e-02
8.5482921e-02   8.5457265e-02   8.5454340e-02   8.5421787e-02   8.5435786e-02
8.5435580e-02   8.5444042e-02   8.5473811e-02   8.5422927e-02   8.5487268e-02;
8.5361147e-02   8.5402595e-02   8.5364780e-02   8.5349264e-02   8.5347219e-02
8.5354795e-02   8.5386922e-02   8.5313091e-02   8.5328438e-02   8.5387947e-02
8.5333814e-02   8.5416971e-02   8.5313495e-02   8.5372827e-02   8.5365065e-02
8.5367421e-02   8.5293467e-02   8.5310955e-02   8.5355878e-02   8.5294088e-02
8.5343336e-02   8.5274581e-02   8.5325596e-02   8.5334829e-02   8.5409598e-02
8.5364052e-02   8.5418329e-02   8.5322368e-02   8.5310743e-02   8.5387669e-02
8.5350547e-02   8.5329817e-02   8.5390875e-02   8.5334035e-02   8.5394297e-02
8.5308456e-02   8.5416724e-02   8.5359425e-02   8.5357912e-02   8.5383861e-02
8.5392024e-02   8.5378854e-02   8.5353182e-02   8.5367753e-02   8.5342715e-02
8.5321992e-02   8.5350933e-02   8.5348798e-02   8.5374101e-02   8.5301061e-02;
8.5112834e-02   8.5084885e-02   8.5187206e-02   8.5228736e-02   8.5099996e-02
8.5047867e-02   8.5120128e-02   8.5163303e-02   8.5117284e-02   8.5153390e-02
8.5087623e-02   8.5101393e-02   8.5129700e-02   8.5053049e-02   8.5083110e-02
8.5056625e-02   8.5127323e-02   8.5157883e-02   8.5172611e-02   8.5102467e-02

```
8.5139631e-02   8.5130555e-02   8.5084975e-02   8.5110574e-02   8.5087434e-02
8.5086892e-02   8.5033352e-02   8.5087772e-02   8.5184439e-02   8.5072127e-02
8.5122165e-02   8.5078645e-02   8.5120582e-02   8.5107752e-02   8.5151229e-02
8.5070997e-02   8.5078908e-02   8.5136141e-02   8.5080820e-02   8.5166152e-02
8.5129641e-02   8.5128041e-02   8.5073457e-02   8.5124187e-02   8.5053569e-02
8.5116058e-02   8.5039419e-02   8.5057221e-02   8.5090939e-02   8.5111029e-02;
   8.4846623e-02   8.4856311e-02   8.4871148e-02   8.4832926e-02   8.4858307e-02
  8.4879830e-02   8.4911192e-02   8.4881823e-02   8.4827805e-02   8.4870167e-02
 8.4871730e-02   8.4877279e-02   8.4904867e-02   8.4791565e-02   8.4936555e-02
8.4820397e-02   8.4892671e-02   8.4902989e-02   8.4849078e-02   8.4926589e-02
8.4809395e-02   8.4877604e-02   8.4885478e-02   8.4837044e-02   8.4893418e-02
8.4892117e-02   8.4893620e-02   8.4886757e-02   8.4810297e-02   8.4876007e-02
8.4894688e-02   8.4936822e-02   8.4893789e-02   8.4902938e-02   8.4800878e-02
8.4874355e-02   8.4912433e-02   8.4876740e-02   8.4853632e-02   8.4880102e-02
8.4841527e-02   8.4880476e-02   8.4839508e-02   8.4851939e-02   8.4830056e-02
8.4848520e-02   8.4897774e-02   8.4862407e-02   8.4887422e-02   8.4903049e-02];
u_max_1 =       [1.8435043e+00   1.8333556e+00   1.8708406e+00   1.8967835e+00
1.8631201e+00   1.8970439e+00   1.8285990e+00   1.8933465e+00   1.8452724e+00
1.8921930e+00   1.8501953e+00   1.8691866e+00   1.8399080e+00   1.8670732e+00
1.8982862e+00   1.8606869e+00   1.8595364e+00   1.8458370e+00   1.8580742e+00
1.8706083e+00   1.8637022e+00   1.8618956e+00   1.8750611e+00   1.8501153e+00
1.8599597e+00   1.8517045e+00   1.8587435e+00   1.8551700e+00   1.8630788e+00
1.8565386e+00   1.8889191e+00   1.9161492e+00   1.8726554e+00   1.8938348e+00
1.8264199e+00   1.8712531e+00   1.8556384e+00   1.9017551e+00   1.8466195e+00
1.9052281e+00   1.9138409e+00   1.8661088e+00   1.8871682e+00   1.8595289e+00
1.8951272e+00   1.8388001e+00   1.9036156e+00   1.8138290e+00   1.8453554e+00
1.8094090e+00;
   2.0408943e+00   2.0446680e+00   2.0374714e+00   1.9872816e+00   2.0212228e+00
  1.9989081e+00   2.0736857e+00   2.0474621e+00   2.0062838e+00   2.0270619e+00
 2.0321186e+00   2.0267929e+00   2.0422988e+00   2.0334972e+00   2.0303409e+00
2.0336855e+00   2.0400499e+00   2.0161752e+00   2.0398509e+00   2.0406352e+00
2.0646687e+00   1.9969363e+00   1.9876849e+00   2.0102918e+00   2.0742396e+00
2.0294981e+00   1.9994900e+00   2.0069114e+00   2.0285787e+00   2.0143326e+00
2.0141389e+00   2.0035183e+00   2.0371930e+00   2.0261320e+00   2.0512181e+00
2.0543063e+00   2.0191148e+00   2.0136120e+00   2.0108397e+00   2.0023317e+00
1.9980168e+00   2.0122638e+00   2.0298162e+00   2.0394913e+00   2.0491865e+00
2.0670245e+00   2.0497464e+00   2.0591788e+00   2.0048325e+00   1.9778314e+00;
   2.1845089e+00   2.1818887e+00   2.1434065e+00   2.1574294e+00   2.1340866e+00
  2.1334536e+00   2.1802384e+00   2.1421260e+00   2.1910306e+00   2.1493858e+00
 2.1566495e+00   2.1730891e+00   2.1491167e+00   2.1706920e+00   2.1764601e+00
2.1241697e+00   2.1881534e+00   2.1920552e+00   2.1924167e+00   2.1350382e+00
2.1800940e+00   2.1705447e+00   2.1845876e+00   2.1873351e+00   2.1478749e+00
2.1724334e+00   2.1690209e+00   2.1876639e+00   2.1907219e+00   2.1410495e+00
2.1875061e+00   2.1984909e+00   2.1995236e+00   2.1451858e+00   2.1491115e+00
2.1186858e+00   2.1185340e+00   2.1641862e+00   2.1379045e+00   2.1688944e+00
2.1880719e+00   2.1599980e+00   2.1527077e+00   2.0905113e+00   2.1543874e+00
2.1727141e+00   2.1594411e+00   2.1799732e+00   2.1399170e+00   2.1665095e+00;
   2.2011528e+00   2.2066676e+00   2.1946244e+00   2.2173041e+00   2.2008797e+00
  2.2232995e+00   2.2255566e+00   2.1843130e+00   2.1894335e+00   2.2127138e+00
 2.1912077e+00   2.2270381e+00   2.1779932e+00   2.2101848e+00   2.2217295e+00
2.1977917e+00   2.1839510e+00   2.1957913e+00   2.1986825e+00   2.1837633e+00
2.2137036e+00   2.2052430e+00   2.2169537e+00   2.1916996e+00   2.2092961e+00
2.2316347e+00   2.2064538e+00   2.2044726e+00   2.1938711e+00   2.1910995e+00
2.1763828e+00   2.1780883e+00   2.1995601e+00   2.1903811e+00   2.2089401e+00
```

```
2.1937077e+00   2.2151057e+00   2.1919994e+00   2.2018451e+00   2.2336559e+00
2.2052914e+00   2.1879544e+00   2.2167559e+00   2.2227412e+00   2.1878818e+00
2.1919717e+00   2.2070830e+00   2.1901848e+00   2.2114185e+00   2.1862702e+00;
   2.1548752e+00   2.1434952e+00   2.1699800e+00   2.1935067e+00   2.1433881e+00
  2.1326108e+00   2.1579257e+00   2.1790011e+00   2.1621647e+00   2.1736592e+00
 2.1374749e+00   2.1445904e+00   2.1512730e+00   2.1240858e+00   2.1369665e+00
2.1217140e+00   2.1547919e+00   2.1690254e+00   2.1835811e+00   2.1411386e+00
2.1601333e+00   2.1517947e+00   2.1372915e+00   2.1533292e+00   2.1524619e+00
2.1244830e+00   2.1309307e+00   2.1424911e+00   2.1758382e+00   2.1328593e+00
2.1568061e+00   2.1368045e+00   2.1564272e+00   2.1544905e+00   2.1583067e+00
2.1288483e+00   2.1353673e+00   2.1672683e+00   2.1336180e+00   2.1607149e+00
2.1549146e+00   2.1547814e+00   2.1298334e+00   2.1426776e+00   2.1341106e+00
2.1563250e+00   2.1322651e+00   2.1340737e+00   2.1376211e+00   2.1549064e+00;
   2.0442656e+00   2.0471458e+00   2.0539281e+00   2.0379412e+00   2.0490698e+00
  2.0629706e+00   2.0747315e+00   2.0598838e+00   2.0341490e+00   2.0509079e+00
 2.0545334e+00   2.0556444e+00   2.0675214e+00   2.0209088e+00   2.0867653e+00
2.0307549e+00   2.0670909e+00   2.0683396e+00   2.0482294e+00   2.0807143e+00
2.0240912e+00   2.0604470e+00   2.0635749e+00   2.0387182e+00   2.0665103e+00
2.0548052e+00   2.0655095e+00   2.0593451e+00   2.0176236e+00   2.0583328e+00
2.0702479e+00   2.0831052e+00   2.0677828e+00   2.0622266e+00   2.0219741e+00
2.0566181e+00   2.0614862e+00   2.0591863e+00   2.0495686e+00   2.0637566e+00
2.0395440e+00   2.0585885e+00   2.0389191e+00   2.0408573e+00   2.0323661e+00
2.0461053e+00   2.0697871e+00   2.0513045e+00   2.0656528e+00   2.0714069e+00];
t_l_max_2 = [5.1439920e-02   5.1519920e-02   5.1583920e-02   5.1007920e-02
5.1311920e-02   5.1279920e-02   5.1855920e-02   5.1423920e-02   5.1631920e-02
5.1231920e-02   5.0863920e-02   5.1519920e-02   5.1311920e-02   5.1263920e-02
5.1695920e-02   5.1167920e-02   5.1375920e-02   5.1151920e-02   5.1727920e-02
5.1679920e-02   5.1503920e-02   5.1391920e-02   5.1407920e-02   5.1199920e-02
5.0927920e-02   5.1375920e-02   5.0751920e-02   5.1343920e-02   5.1327920e-02
5.1119920e-02   5.1215920e-02   5.1599920e-02   5.0863920e-02   5.1519920e-02
5.1391920e-02   5.1551920e-02   5.1007920e-02   5.1647920e-02   5.0991920e-02
5.1215920e-02   5.1311920e-02   5.1327920e-02   5.1423920e-02   5.1519920e-02
5.0975920e-02   5.1343920e-02   5.1535920e-02   5.2079920e-02   5.1119920e-02
5.1647920e-02;
   5.3039920e-02   5.2655920e-02   5.2527920e-02   5.2751920e-02   5.2655920e-02
  5.2847920e-02   5.2255920e-02   5.2751920e-02   5.2719920e-02   5.2511920e-02
 5.2383920e-02   5.2735920e-02   5.2639920e-02   5.3119920e-02   5.2527920e-02
5.2511920e-02   5.2479920e-02   5.2591920e-02   5.2511920e-02   5.2431920e-02
5.2511920e-02   5.2127920e-02   5.2367920e-02   5.2703920e-02   5.3087920e-02
5.3359920e-02   5.2559920e-02   5.3007920e-02   5.2079920e-02   5.2799920e-02
5.2799920e-02   5.2703920e-02   5.2415920e-02   5.2767920e-02   5.2287920e-02
5.2559920e-02   5.2799920e-02   5.2015920e-02   5.2815920e-02   5.2735920e-02
5.2431920e-02   5.2815920e-02   5.2943920e-02   5.2591920e-02   5.2719920e-02
5.3007920e-02   5.2575920e-02   5.2223920e-02   5.2495920e-02   5.2751920e-02;
   5.5311920e-02   5.4447920e-02   5.4591920e-02   5.4879920e-02   5.4367920e-02
  5.4415920e-02   5.4687920e-02   5.4383920e-02   5.4671920e-02   5.4671920e-02
 5.4463920e-02   5.4383920e-02   5.4479920e-02   5.4127920e-02   5.4223920e-02
5.4623920e-02   5.4639920e-02   5.4479920e-02   5.4143920e-02   5.4319920e-02
5.4239920e-02   5.4607920e-02   5.4463920e-02   5.4527920e-02   5.4287920e-02
5.4191920e-02   5.4463920e-02   5.4623920e-02   5.4447920e-02   5.4703920e-02
5.4847920e-02   5.4735920e-02   5.3919920e-02   5.4591920e-02   5.4447920e-02
5.4751920e-02   5.4703920e-02   5.4447920e-02   5.4591920e-02   5.5007920e-02
5.4735920e-02   5.4399920e-02   5.4703920e-02   5.4655920e-02   5.4271920e-02
5.4559920e-02   5.4591920e-02   5.4495920e-02   5.4607920e-02   5.4719920e-02;
```

```
   5.7631920e-02   5.7103920e-02   5.7183920e-02   5.7327920e-02   5.7359920e-02
  5.7295920e-02   5.7135920e-02   5.7199920e-02   5.6863920e-02   5.7103920e-02
 5.7039920e-02   5.6879920e-02   5.7423920e-02   5.7215920e-02   5.6991920e-02
5.7343920e-02   5.7199920e-02   5.7151920e-02   5.7407920e-02   5.7279920e-02
5.7343920e-02   5.7311920e-02   5.6943920e-02   5.7311920e-02   5.7279920e-02
5.6959920e-02   5.6943920e-02   5.6783920e-02   5.6927920e-02   5.7103920e-02
5.7167920e-02   5.6943920e-02   5.6959920e-02   5.7119920e-02   5.7455920e-02
5.6783920e-02   5.7455920e-02   5.7663920e-02   5.6783920e-02   5.7311920e-02
5.6975920e-02   5.7343920e-02   5.7039920e-02   5.7087920e-02   5.7199920e-02
5.6991920e-02   5.7519920e-02   5.7055920e-02   5.6767920e-02   5.7391920e-02;
   6.0271920e-02   5.9903920e-02   6.0111920e-02   5.9983920e-02   6.0591920e-02
  6.0255920e-02   6.0511920e-02   6.0463920e-02   6.0511920e-02   6.0991920e-02
 6.0143920e-02   6.0239920e-02   6.0063920e-02   6.0047920e-02   6.0271920e-02
6.0207920e-02   6.0431920e-02   6.0319920e-02   6.0575920e-02   6.0335920e-02
6.0063920e-02   6.0431920e-02   6.0383920e-02   6.0047920e-02   6.0255920e-02
6.0111920e-02   5.9935920e-02   6.0447920e-02   5.9871920e-02   6.0463920e-02
5.9727920e-02   6.0575920e-02   6.0031920e-02   6.0847920e-02   6.0047920e-02
6.0271920e-02   6.0111920e-02   5.9631920e-02   6.0255920e-02   5.9935920e-02
5.9951920e-02   6.0719920e-02   6.0223920e-02   6.0479920e-02   6.0207920e-02
5.9791920e-02   5.9967920e-02   6.0351920e-02   6.0367920e-02   6.0063920e-02;
   6.3647920e-02   6.3871920e-02   6.3519920e-02   6.3215920e-02   6.3455920e-02
  6.3871920e-02   6.3263920e-02   6.3759920e-02   6.3631920e-02   6.3631920e-02
 6.3375920e-02   6.3615920e-02   6.3231920e-02   6.3695920e-02   6.3343920e-02
6.3311920e-02   6.3871920e-02   6.3439920e-02   6.3599920e-02   6.3375920e-02
6.3599920e-02   6.3535920e-02   6.3295920e-02   6.3231920e-02   6.3311920e-02
6.3391920e-02   6.3535920e-02   6.3439920e-02   6.3823920e-02   6.3215920e-02
6.3551920e-02   6.4159920e-02   6.3775920e-02   6.3583920e-02   6.3263920e-02
6.3423920e-02   6.3375920e-02   6.3359920e-02   6.3775920e-02   6.3215920e-02
6.3599920e-02   6.3487920e-02   6.3471920e-02   6.3359920e-02   6.3247920e-02
6.3711920e-02   6.3711920e-02   6.3727920e-02   6.3535920e-02   6.3679920e-02];
t_u_max_2 = [3.9551920e-02   3.9631920e-02   3.9631920e-02   3.8559920e-02
3.9295920e-02   3.8879920e-02   3.9823920e-02   3.9615920e-02   3.9263920e-02
3.9055920e-02   3.8783920e-02   3.9119920e-02   3.8943920e-02   3.9151920e-02
3.9503920e-02   3.9023920e-02   3.9103920e-02   3.8831920e-02   3.9775920e-02
3.9487920e-02   3.9567920e-02   3.9343920e-02   3.9263920e-02   3.9247920e-02
3.8927920e-02   3.9327920e-02   3.8831920e-02   3.9231920e-02   3.9007920e-02
3.9087920e-02   3.9087920e-02   3.9471920e-02   3.8943920e-02   3.9775920e-02
3.9631920e-02   3.9503920e-02   3.8735920e-02   3.9519920e-02   3.8895920e-02
3.9343920e-02   3.9135920e-02   3.9439920e-02   3.9423920e-02   3.9631920e-02
3.9087920e-02   3.9343920e-02   3.9231920e-02   3.9807920e-02   3.8815920e-02
3.9919920e-02;
   4.1343920e-02   4.0927920e-02   4.0655920e-02   4.0767920e-02   4.0799920e-02
  4.0799920e-02   4.0591920e-02   4.0943920e-02   4.0895920e-02   4.0575920e-02
 4.0719920e-02   4.0959920e-02   4.0815920e-02   4.1455920e-02   4.0847920e-02
4.0703920e-02   4.0687920e-02   4.0447920e-02   4.0783920e-02   4.0703920e-02
4.0719920e-02   4.0319920e-02   4.0735920e-02   4.0927920e-02   4.1247920e-02
4.1647920e-02   4.0783920e-02   4.1279920e-02   4.0351920e-02   4.0719920e-02
4.1055920e-02   4.0831920e-02   4.0735920e-02   4.0879920e-02   4.0687920e-02
4.0895920e-02   4.0863920e-02   4.0415920e-02   4.1135920e-02   4.1103920e-02
4.0799920e-02   4.0943920e-02   4.1135920e-02   4.0527920e-02   4.1055920e-02
4.1359920e-02   4.1023920e-02   4.0575920e-02   4.0639920e-02   4.1023920e-02;
   4.3775920e-02   4.3103920e-02   4.3263920e-02   4.3375920e-02   4.2879920e-02
  4.2895920e-02   4.3247920e-02   4.3039920e-02   4.3327920e-02   4.3247920e-02
 4.2959920e-02   4.2991920e-02   4.2911920e-02   4.2607920e-02   4.2831920e-02
```

```
4.3039920e-02   4.3231920e-02   4.3103920e-02   4.2687920e-02   4.2943920e-02
4.2863920e-02   4.3199920e-02   4.3071920e-02   4.3103920e-02   4.2943920e-02
4.2815920e-02   4.2959920e-02   4.3151920e-02   4.2959920e-02   4.3295920e-02
4.3407920e-02   4.3311920e-02   4.2495920e-02   4.3247920e-02   4.2943920e-02
4.3359920e-02   4.3247920e-02   4.2959920e-02   4.2975920e-02   4.3455920e-02
4.3327920e-02   4.3007920e-02   4.3423920e-02   4.3311920e-02   4.2831920e-02
4.3039920e-02   4.3183920e-02   4.2991920e-02   4.3103920e-02   4.3231920e-02;
   4.6383920e-02   4.5871920e-02   4.5935920e-02   4.6127920e-02   4.6127920e-02
  4.6063920e-02   4.5855920e-02   4.5935920e-02   4.5439920e-02   4.5839920e-02
 4.5919920e-02   4.5711920e-02   4.6255920e-02   4.5999920e-02   4.5759920e-02
4.6159920e-02   4.6031920e-02   4.5935920e-02   4.6303920e-02   4.6063920e-02
4.6127920e-02   4.6031920e-02   4.5647920e-02   4.6047920e-02   4.5951920e-02
4.5791920e-02   4.5727920e-02   4.5567920e-02   4.5647920e-02   4.5887920e-02
4.5951920e-02   4.5679920e-02   4.5759920e-02   4.5839920e-02   4.6287920e-02
4.5535920e-02   4.6287920e-02   4.6495920e-02   4.5631920e-02   4.6031920e-02
4.5807920e-02   4.6063920e-02   4.5823920e-02   4.5871920e-02   4.6031920e-02
4.5823920e-02   4.6319920e-02   4.5823920e-02   4.5487920e-02   4.6223920e-02;
   4.9087920e-02   4.8735920e-02   4.8959920e-02   4.8831920e-02   4.9359920e-02
  4.9103920e-02   4.9407920e-02   4.9327920e-02   4.9327920e-02   4.9807920e-02
 4.8959920e-02   4.9071920e-02   4.8943920e-02   4.8879920e-02   4.9071920e-02
4.9071920e-02   4.9231920e-02   4.9135920e-02   4.9375920e-02   4.9119920e-02
4.8911920e-02   4.9119920e-02   4.9199920e-02   4.8895920e-02   4.9119920e-02
4.8927920e-02   4.8751920e-02   4.9279920e-02   4.8719920e-02   4.9311920e-02
4.8543920e-02   4.9407920e-02   4.8831920e-02   4.9679920e-02   4.8879920e-02
4.9087920e-02   4.8895920e-02   4.8447920e-02   4.9119920e-02   4.8719920e-02
4.8815920e-02   4.9567920e-02   4.8991920e-02   4.9279920e-02   4.9071920e-02
4.8607920e-02   4.8719920e-02   4.9135920e-02   4.9199920e-02   4.8895920e-02;
   5.2447920e-02   5.2655920e-02   5.2335920e-02   5.1983920e-02   5.2207920e-02
  5.2559920e-02   5.1999920e-02   5.2511920e-02   5.2415920e-02   5.2415920e-02
 5.2143920e-02   5.2399920e-02   5.1951920e-02   5.2447920e-02   5.2127920e-02
5.2031920e-02   5.2671920e-02   5.2191920e-02   5.2367920e-02   5.2127920e-02
5.2335920e-02   5.2319920e-02   5.2031920e-02   5.2047920e-02   5.2111920e-02
5.2191920e-02   5.2367920e-02   5.2255920e-02   5.2639920e-02   5.2031920e-02
5.2335920e-02   5.2927920e-02   5.2559920e-02   5.2351920e-02   5.2031920e-02
5.2239920e-02   5.2159920e-02   5.2143920e-02   5.2559920e-02   5.1983920e-02
5.2415920e-02   5.2239920e-02   5.2175920e-02   5.2127920e-02   5.2047920e-02
5.2527920e-02   5.2495920e-02   5.2479920e-02   5.2303920e-02   5.2463920e-02];
l_max_2 =   [8.5201095e-02   8.5160402e-02   8.5175657e-02   8.5148676e-02
8.5207839e-02   8.5127063e-02   8.5162662e-02   8.5234415e-02   8.5122394e-02
8.5148538e-02   8.5218143e-02   8.5162478e-02   8.5100634e-02   8.5171948e-02
8.5140346e-02   8.5172942e-02   8.5166052e-02   8.5160181e-02   8.5155215e-02
8.5128041e-02   8.5171722e-02   8.5192921e-02   8.5208204e-02   8.5207376e-02
8.5226597e-02   8.5192884e-02   8.5217180e-02   8.5175263e-02   8.5163153e-02
8.5218194e-02   8.5205649e-02   8.5204478e-02   8.5176630e-02   8.5180895e-02
8.5220838e-02   8.5183274e-02   8.5140122e-02   8.5148537e-02   8.5228156e-02
8.5214410e-02   8.5198496e-02   8.5192992e-02   8.5161411e-02   8.5196974e-02
8.5239006e-02   8.5199263e-02   8.5135103e-02   8.5090795e-02   8.5160292e-02
8.5197990e-02;
   8.5297014e-02   8.5398801e-02   8.5371313e-02   8.5336758e-02   8.5336845e-02
  8.5308323e-02   8.5398121e-02   8.5379262e-02   8.5356518e-02   8.5304973e-02
 8.5398476e-02   8.5384155e-02   8.5347160e-02   8.5356901e-02   8.5359285e-02
8.5398881e-02   8.5345177e-02   8.5341105e-02   8.5370648e-02   8.5409250e-02
8.5377823e-02   8.5368252e-02   8.5392928e-02   8.5379567e-02   8.5339438e-02
8.5375594e-02   8.5378717e-02   8.5312806e-02   8.5373248e-02   8.5287261e-02
```

8.5338800e-02   8.5350072e-02   8.5389904e-02   8.5318532e-02   8.5365120e-02
8.5409853e-02   8.5320432e-02   8.5415672e-02   8.5387912e-02   8.5446396e-02
8.5394897e-02   8.5354770e-02   8.5360422e-02   8.5352937e-02   8.5338398e-02
8.5394538e-02   8.5428683e-02   8.5436274e-02   8.5393533e-02   8.5393984e-02;
8.5392569e-02   8.5447638e-02   8.5463609e-02   8.5432233e-02   8.5427871e-02
8.5464924e-02   8.5528763e-02   8.5521052e-02   8.5457391e-02   8.5448153e-02
8.5432010e-02   8.5471209e-02   8.5455396e-02   8.5448411e-02   8.5482126e-02
8.5432203e-02   8.5439652e-02   8.5436048e-02   8.5453986e-02   8.5486882e-02
8.5475886e-02   8.5515363e-02   8.5440314e-02   8.5449756e-02   8.5529602e-02
8.5479148e-02   8.5474432e-02   8.5423155e-02   8.5482449e-02   8.5468990e-02
8.5474558e-02   8.5506396e-02   8.5500379e-02   8.5502947e-02   8.5496077e-02
8.5487890e-02   8.5453340e-02   8.5469217e-02   8.5434504e-02   8.5434227e-02
8.5460387e-02   8.5445954e-02   8.5503272e-02   8.5467533e-02   8.5469578e-02
8.5464873e-02   8.5415804e-02   8.5388904e-02   8.5457928e-02   8.5430558e-02;
8.5392553e-02   8.5337579e-02   8.5400023e-02   8.5353212e-02   8.5343154e-02
8.5367901e-02   8.5350209e-02   8.5319132e-02   8.5369349e-02   8.5368471e-02
8.5340710e-02   8.5384901e-02   8.5417277e-02   8.5376541e-02   8.5336823e-02
8.5274239e-02   8.5315350e-02   8.5346879e-02   8.5318262e-02   8.5373249e-02
8.5337020e-02   8.5359503e-02   8.5320624e-02   8.5412428e-02   8.5376564e-02
8.5442128e-02   8.5315148e-02   8.5370760e-02   8.5364900e-02   8.5378360e-02
8.5335837e-02   8.5351622e-02   8.5367618e-02   8.5447387e-02   8.5355613e-02
8.5343774e-02   8.5310911e-02   8.5269177e-02   8.5397083e-02   8.5410067e-02
8.5296405e-02   8.5349989e-02   8.5353363e-02   8.5382949e-02   8.5350210e-02
8.5378357e-02   8.5395535e-02   8.5339252e-02   8.5403100e-02   8.5349307e-02;
8.5150210e-02   8.5139256e-02   8.5087975e-02   8.5134972e-02   8.5086141e-02
8.5052399e-02   8.5083010e-02   8.5120433e-02   8.5135308e-02   8.5116248e-02
8.5153962e-02   8.5163806e-02   8.5147258e-02   8.5185882e-02   8.5155429e-02
8.5090147e-02   8.5135645e-02   8.5082089e-02   8.5096233e-02   8.5114208e-02
8.5144238e-02   8.5124788e-02   8.5024780e-02   8.5174565e-02   8.5184945e-02
8.5016282e-02   8.5160291e-02   8.5157478e-02   8.5150265e-02   8.5062861e-02
8.5178061e-02   8.5064480e-02   8.5073610e-02   8.5151508e-02   8.5113648e-02
8.5065135e-02   8.5013961e-02   8.5120130e-02   8.5138436e-02   8.5079775e-02
8.5140235e-02   8.5119196e-02   8.5077388e-02   8.5054651e-02   8.5147552e-02
8.5137766e-02   8.5105146e-02   8.5147447e-02   8.5109472e-02   8.5110197e-02;
8.4918436e-02   8.4888655e-02   8.4930362e-02   8.4897312e-02   8.4858587e-02
8.4767248e-02   8.4876236e-02   8.4847549e-02   8.4888690e-02   8.4843219e-02
8.4911404e-02   8.4910217e-02   8.4793289e-02   8.4821895e-02   8.4911866e-02
8.4881005e-02   8.4851979e-02   8.4829704e-02   8.4905639e-02   8.4870280e-02
8.4821291e-02   8.4868641e-02   8.4841758e-02   8.4937091e-02   8.4912338e-02
8.4875181e-02   8.4906872e-02   8.4842582e-02   8.4925370e-02   8.4908260e-02
8.4874055e-02   8.4893840e-02   8.4867312e-02   8.4880145e-02   8.4907759e-02
8.4931080e-02   8.4868622e-02   8.4898974e-02   8.4843631e-02   8.4943465e-02
8.4940735e-02   8.4891539e-02   8.4861469e-02   8.4910961e-02   8.4908383e-02
8.4845243e-02   8.4840380e-02   8.4839258e-02   8.4868979e-02   8.4899584e-02];
u_max_2 =   [1.8738193e+00   1.8372561e+00   1.8627738e+00   1.8540118e+00
1.8830638e+00   1.8348386e+00   1.8455134e+00   1.8920462e+00   1.8373010e+00
1.8378603e+00   1.8879601e+00   1.8670885e+00   1.8264722e+00   1.8635479e+00
1.8391009e+00   1.8702837e+00   1.8503841e+00   1.8711441e+00   1.8464105e+00
1.8301538e+00   1.8578093e+00   1.8673288e+00   1.8777439e+00   1.8818170e+00
1.9079893e+00   1.8744502e+00   1.8890600e+00   1.8650543e+00   1.8647158e+00
1.8873395e+00   1.8945758e+00   1.8766755e+00   1.8660337e+00   1.8511855e+00
1.8838384e+00   1.8681919e+00   1.8587327e+00   1.8348497e+00   1.8952314e+00
1.8835669e+00   1.8776996e+00   1.8552421e+00   1.8422949e+00   1.8660531e+00
1.9194526e+00   1.8747632e+00   1.8419793e+00   1.8005876e+00   1.8637946e+00

```
1.8567032e+00;
   1.9822878e+00   2.0723928e+00   2.0346561e+00   2.0012904e+00   2.0178276e+00
  1.9842512e+00   2.0511189e+00   2.0442541e+00   2.0084774e+00   1.9957293e+00
 2.0700591e+00   2.0440581e+00   2.0215099e+00   2.0069034e+00   2.0226184e+00
2.0462347e+00   2.0277749e+00   2.0342060e+00   2.0460286e+00   2.0723008e+00
2.0410187e+00   2.0614270e+00   2.0603473e+00   2.0356274e+00   2.0114264e+00
2.0289230e+00   2.0372596e+00   1.9803476e+00   2.0315564e+00   1.9888501e+00
2.0234564e+00   2.0238919e+00   2.0562976e+00   1.9884000e+00   2.0282102e+00
2.0522792e+00   1.9955115e+00   2.0602423e+00   2.0483815e+00   2.0948979e+00
2.0485093e+00   2.0144604e+00   2.0239725e+00   2.0262696e+00   2.0127293e+00
2.0311820e+00   2.0808106e+00   2.0919855e+00   2.0561820e+00   2.0612852e+00;
   2.1265084e+00   2.1783624e+00   2.1710351e+00   2.1661468e+00   2.1546654e+00
  2.1679272e+00   2.1862822e+00   2.2033329e+00   2.1975343e+00   2.1693795e+00
 2.1315928e+00   2.1626407e+00   2.1448675e+00   2.1403987e+00   2.1876544e+00
2.1387813e+00   2.1709697e+00   2.1862211e+00   2.1685405e+00   2.2118752e+00
2.1756831e+00   2.2049347e+00   2.1495819e+00   2.1613306e+00   2.2216680e+00
2.1633920e+00   2.1929071e+00   2.1557279e+00   2.1673283e+00   2.1799573e+00
2.1565961e+00   2.1787377e+00   2.1937282e+00   2.1868471e+00   2.1703613e+00
2.1734352e+00   2.1589923e+00   2.1838388e+00   2.1401453e+00   2.1514612e+00
2.1863803e+00   2.1675789e+00   2.2073049e+00   2.1769937e+00   2.1683357e+00
2.1709736e+00   2.1173342e+00   2.1373941e+00   2.1825655e+00   2.1552325e+00;
   2.2207562e+00   2.2106983e+00   2.2133646e+00   2.2022632e+00   2.2034222e+00
  2.2197370e+00   2.1898495e+00   2.1959525e+00   2.1855003e+00   2.1861063e+00
 2.2249646e+00   2.2276772e+00   2.2548111e+00   2.2042518e+00   2.2038405e+00
2.1759342e+00   2.2096698e+00   2.2006212e+00   2.2222221e+00   2.2148574e+00
2.2032457e+00   2.2166334e+00   2.1838554e+00   2.2305168e+00   2.1957398e+00
2.2417375e+00   2.2026545e+00   2.2130852e+00   2.1948017e+00   2.2025074e+00
2.2006565e+00   2.1983810e+00   2.2092991e+00   2.2255386e+00   2.2172391e+00
2.1934684e+00   2.1998048e+00   2.1968240e+00   2.2222871e+00   2.2173313e+00
2.2194298e+00   2.2053582e+00   2.2084201e+00   2.2220316e+00   2.2183230e+00
2.2223911e+00   2.2243770e+00   2.2077151e+00   2.2120416e+00   2.2028317e+00;
   2.1746206e+00   2.1546372e+00   2.1290298e+00   2.1519364e+00   2.1346522e+00
  2.1257899e+00   2.1542305e+00   2.1658247e+00   2.1571197e+00   2.1571946e+00
 2.1598588e+00   2.1613915e+00   2.1664582e+00   2.1719959e+00   2.1675385e+00
2.1404979e+00   2.1417584e+00   2.1423082e+00   2.1399739e+00   2.1400218e+00
2.1588972e+00   2.1410380e+00   2.1171177e+00   2.1767658e+00   2.1792765e+00
2.1234574e+00   2.1617362e+00   2.1691872e+00   2.1661017e+00   2.1379022e+00
2.1727966e+00   2.1366908e+00   2.1346419e+00   2.1589532e+00   2.1553040e+00
2.1414408e+00   2.1211634e+00   2.1497602e+00   2.1620916e+00   2.1417316e+00
2.1632820e+00   2.1492048e+00   2.1396263e+00   2.1271682e+00   2.1659312e+00
2.1674791e+00   2.1484130e+00   2.1590944e+00   2.1508819e+00   2.1495491e+00;
   2.0768337e+00   2.0649679e+00   2.0793239e+00   2.0687826e+00   2.0509979e+00
  2.0065139e+00   2.0580415e+00   2.0429225e+00   2.0620194e+00   2.0464228e+00
 2.0719991e+00   2.0712509e+00   2.0195116e+00   2.0308201e+00   2.0738539e+00
2.0535200e+00   2.0445922e+00   2.0346418e+00   2.0689996e+00   2.0529139e+00
2.0318911e+00   2.0577330e+00   2.0389554e+00   2.0873931e+00   2.0752284e+00
2.0549154e+00   2.0674333e+00   2.0386627e+00   2.0797979e+00   2.0704660e+00
2.0579461e+00   2.0668739e+00   2.0551791e+00   2.0566666e+00   2.0785607e+00
2.0815434e+00   2.0565574e+00   2.0685172e+00   2.0447459e+00   2.0859607e+00
2.0919020e+00   2.0627128e+00   2.0444072e+00   2.0722707e+00   2.0734375e+00
2.0424001e+00   2.0363081e+00   2.0351798e+00   2.0555381e+00   2.0682519e+00];

 delay_1  delay_2   mu1_mean mu1_median   mu1_mode mu1_HDIlow mu1_HDIhigh
Vdet1 mu1_pcgtVdet1 mu1_ROPElow mu1_ROPEhigh mu1_pcInROPE
```

```
     -10       -5     1.8649     1.8649     1.8649     1.8600      1.8695
1.8440        100.00      1.8340       1.8540         0.00
     -10        0     1.8649     1.8649     1.8646     1.8601      1.8696
1.8440        100.00      1.8340       1.8540         0.00
     -10        5     1.8648     1.8648     1.8648     1.8601      1.8697
1.8440        100.00      1.8340       1.8540         0.00
     -10       10     1.8649     1.8649     1.8648     1.8600      1.8696
1.8440        100.00      1.8340       1.8540         0.00
     -10       15     1.8649     1.8649     1.8651     1.8600      1.8695
1.8440        100.00      1.8340       1.8540         0.00
      -5        0     2.0303     2.0303     2.0305     2.0252      2.0355
2.0620          0.00      2.0520       2.0720         0.00
      -5        5     2.0303     2.0303     2.0305     2.0249      2.0353
2.0620          0.00      2.0520       2.0720         0.00
      -5       10     2.0303     2.0303     2.0303     2.0250      2.0354
2.0620          0.00      2.0520       2.0720         0.00
      -5       15     2.0303     2.0303     2.0301     2.0251      2.0354
2.0620          0.00      2.0520       2.0720         0.00
       0        5     2.1667     2.1667     2.1667     2.1619      2.1713
2.2540          0.00      2.2440       2.2640         0.00
       0       10     2.1666     2.1667     2.1668     2.1618      2.1712
2.2540          0.00      2.2440       2.2640         0.00
       0       15     2.1667     2.1667     2.1668     2.1619      2.1713
2.2540          0.00      2.2440       2.2640         0.00
       5       10     2.2057     2.2057     2.2058     2.2026      2.2087
2.2920          0.00      2.2820       2.3020         0.00
       5       15     2.2057     2.2057     2.2057     2.2027      2.2087
2.2920          0.00      2.2820       2.3020         0.00
      10       15     2.1501     2.1502     2.1502     2.1469      2.1534
2.1730          0.00      2.1630       2.1830         0.00

 delay_1  delay_2   mu2_mean mu2_median   mu2_mode mu2_HDIlow mu2_HDIhigh
Vdet2 mu2_pcgtVdet2 mu2_ROPElow mu2_ROPEhigh mu2_pcInROPE
     -10       -5     2.0303     2.0303     2.0303     2.0250      2.0354
2.0620          0.00      2.0520       2.0720         0.00
     -10        0     2.1667     2.1667     2.1668     2.1620      2.1715
2.2540          0.00      2.2440       2.2640         0.00
     -10        5     2.2057     2.2057     2.2055     2.2026      2.2087
2.2920          0.00      2.2820       2.3020         0.00
     -10       10     2.1502     2.1502     2.1500     2.1470      2.1534
2.1730          0.00      2.1630       2.1830         0.00
     -10       15     2.0565     2.0565     2.0566     2.0530      2.0599
2.0460        100.00      2.0360       2.0560        38.04
      -5        0     2.1667     2.1667     2.1668     2.1620      2.1714
2.2540          0.00      2.2440       2.2640         0.00
      -5        5     2.2057     2.2057     2.2057     2.2027      2.2088
2.2920          0.00      2.2820       2.3020         0.00
      -5       10     2.1502     2.1502     2.1500     2.1468      2.1533
2.1730          0.00      2.1630       2.1830         0.00
      -5       15     2.0565     2.0565     2.0565     2.0531      2.0600
2.0460        100.00      2.0360       2.0560        38.41
       0        5     2.2057     2.2057     2.2058     2.2027      2.2087
2.2920          0.00      2.2820       2.3020         0.00
       0       10     2.1502     2.1502     2.1501     2.1469      2.1533
```

```
2.1730          0.00      2.1630       2.1830        0.00
       0       15      2.0565     2.0565     2.0566     2.0530      2.0599
2.0460        100.00      2.0360       2.0560       37.88
       5       10      2.1502     2.1502     2.1501     2.1470      2.1534
2.1730          0.00      2.1630       2.1830        0.00
       5       15      2.0565     2.0565     2.0566     2.0530      2.0600
2.0460        100.00      2.0360       2.0560       37.89
      10       15      2.0565     2.0565     2.0565     2.0530      2.0600
2.0460        100.00      2.0360       2.0560       38.66

 delay_1  delay_2 muDiff_mean muDiff_median muDiff_mode muDiff_HDIlow
muDiff_HDIhigh muDiff_pcgtZero muDiff_ROPElow muDiff_ROPEhigh   muDiff_pcInROPE
     -10       -5     -0.1654       -0.1654     -0.1653       -0.1724
-0.1583           0.00        -0.0100          0.0100             0.00
     -10        0     -0.3018       -0.3018     -0.3017       -0.3085
-0.2951           0.00        -0.0100          0.0100             0.00
     -10        5     -0.3408       -0.3408     -0.3408       -0.3465
-0.3352           0.00        -0.0100          0.0100             0.00
     -10       10     -0.2853       -0.2853     -0.2852       -0.2910
-0.2795           0.00        -0.0100          0.0100             0.00
     -10       15     -0.1917       -0.1917     -0.1919       -0.1976
-0.1857           0.00        -0.0100          0.0100             0.00
      -5        0     -0.1364       -0.1364     -0.1364       -0.1433
-0.1294           0.00        -0.0100          0.0100             0.00
      -5        5     -0.1754       -0.1754     -0.1754       -0.1815
-0.1695           0.00        -0.0100          0.0100             0.00
      -5       10     -0.1199       -0.1199     -0.1201       -0.1260
-0.1138           0.00        -0.0100          0.0100             0.00
      -5       15     -0.0262       -0.0263     -0.0264       -0.0325
-0.0201           0.00        -0.0100          0.0100             0.00
       0        5     -0.0390       -0.0390     -0.0391       -0.0446
-0.0334           0.00        -0.0100          0.0100             0.00
       0       10      0.0165        0.0165      0.0165        0.0108
0.0221          100.00       -0.0100          0.0100             1.30
       0       15      0.1102        0.1102      0.1099        0.1043
0.1160          100.00       -0.0100          0.0100             0.00
       5       10      0.0555        0.0555      0.0556        0.0512
0.0600          100.00       -0.0100          0.0100             0.00
       5       15      0.1492        0.1492      0.1493        0.1446
0.1538          100.00       -0.0100          0.0100             0.00
      10       15      0.0936        0.0937      0.0938        0.0888
0.0983          100.00       -0.0100          0.0100             0.00

  delay_1  delay_2    nu_mean  nu_median    nu_mode  nu_HDIlow nu_HDIhigh
nuLog10_mean nuLog10_median nuLog10_mode nuLog10_HDIlow nuLog10_HDIhigh
     -10       -5    48.5997    39.8374    26.0374     6.3079   114.6550
1.5952         1.6003       1.5988        1.0404          2.1392
     -10        0    43.2875    34.7878    20.0526     5.2733   105.0950
1.5375         1.5414       1.5592        0.9623          2.0954
     -10        5    45.6046    36.8858    20.3809     5.9301   109.6040
1.5638         1.5669       1.5940        0.9996          2.1152
     -10       10    50.8664    42.2317    25.5705     6.6868   117.2770
1.6206         1.6256       1.6580        1.0750          2.1407
     -10       15    45.8627    37.2034    22.1082     5.5564   109.8980
```

```
1.5650         1.5706       1.5574        0.9906          2.1181
      -5         0    46.1347    37.6391    22.0523     5.9124   109.9850
1.5706         1.5756       1.5667        1.0066          2.1128
      -5         5    47.8912    39.4521    25.3105     6.3731   112.1480
1.5913         1.5961       1.5690        1.0471          2.1302
      -5        10    52.9570    44.5263    30.1174     7.7475   120.2950
1.6432         1.6486       1.6317        1.1192          2.1581
      -5        15    48.4718    39.8465    23.7617     6.0519   114.2820
1.5945         1.6004       1.5969        1.0405          2.1393
       0         5    42.9702    34.5492    21.1484     5.6210   103.7330
1.5367         1.5384       1.5413        0.9794          2.0949
       0        10    48.3925    39.9705    24.7773     6.4590   112.8410
1.5966         1.6017       1.6466        1.0472          2.1218
       0        15    43.3102    34.6981    22.2099     5.1975   105.3440
1.5376         1.5403       1.5232        0.9667          2.1001
       5        10    50.3870    41.7227    25.7867     6.8593   116.7230
1.6172         1.6204       1.6213        1.0891          2.1455
       5        15    45.6342    36.9518    23.0210     5.7716   109.1230
1.5644         1.5676       1.5640        0.9953          2.1072
      10        15    50.9890    42.4081    24.8664     7.2371   118.4680
1.6216         1.6274       1.6582        1.0790          2.1435

 delay_1  delay_2 effSz_mean effSz_median effSz_mode effSz_HDIlow effSz_HDIhigh
effSz_pcgtZero effSz_ROPElow effSz_ROPEhigh effSz_pcInROPE
     -10       -5    -6.7511      -6.7411    -6.7227      -7.5708       -5.9774
       0.0000       -0.1000         0.1000         0.0000
     -10        0   -13.0153     -12.9866   -12.9382     -14.5617      -11.5249
       0.0000       -0.1000         0.1000         0.0000
     -10        5   -17.3698     -17.3424   -17.3026     -19.4887      -15.2782
       0.0000       -0.1000         0.1000         0.0000
     -10       10   -14.2248     -14.2112   -14.1916     -15.9000      -12.5833
       0.0000       -0.1000         0.1000         0.0000
     -10       15    -9.3679      -9.3526    -9.3589     -10.5146       -8.2304
       0.0000       -0.1000         0.1000         0.0000
      -5        0    -5.6227      -5.6127    -5.5966      -6.3213       -4.9333
       0.0000       -0.1000         0.1000         0.0000
      -5        5    -8.4183      -8.4086    -8.3382      -9.4513       -7.3604
       0.0000       -0.1000         0.1000         0.0000
      -5       10    -5.6420      -5.6381    -5.6362      -6.3599       -4.9417
       0.0000       -0.1000         0.1000         0.0000
      -5       15    -1.2141      -1.2130    -1.2178      -1.5348       -0.8899
       0.0000       -0.1000         0.1000         0.0000
       0        5    -2.0177      -2.0162    -1.9959      -2.3875       -1.6475
       0.0000       -0.1000         0.1000         0.0000
       0       10     0.8341       0.8335     0.8441       0.5349        1.1442
     100.0000       -0.1000         0.1000         0.0000
       0       15     5.4601       5.4489     5.4193       4.7715        6.1929
     100.0000       -0.1000         0.1000         0.0000
       5       10     3.6067       3.6033     3.5966       3.1294        4.0924
     100.0000       -0.1000         0.1000         0.0000
       5       15     9.3463       9.3316     9.2907       8.2732       10.4209
     100.0000       -0.1000         0.1000         0.0000
      10       15     5.6877       5.6820     5.6867       5.0124        6.3559
     100.0000       -0.1000         0.1000         0.0000
```

delay_1	delay_2	sigma1_mean	sigma1_median	sigma1_mode	sigma1_HDIlow	sigma1_HDIhigh	sigma2_mean	sigma2_median	sigma2_mode	sigma2_HDIlow	sigma2_HDIhigh	sigmaDiff_mean	sigmaDiff_median	sigmaDiff_mode	sigmaDiff_HDIlow	sigmaDiff_HDIhigh	sigmaDiff_pcgtZero
-10	-5	0.023535	0.023444	0.023197	0.019988	0.027123	0.025509	0.025409	0.025072	0.021760	0.029405	-0.001974	-0.001959	-0.001916	-0.007065	0.003161	22.24
-10	0	0.023436	0.023352	0.022988	0.019923	0.027099	0.023033	0.022951	0.022721	0.019505	0.026650	0.000403	0.000400	0.000275	-0.004478	0.005296	56.47
-10	5	0.023493	0.023403	0.023123	0.020032	0.027218	0.014905	0.014845	0.014754	0.012690	0.017232	0.008588	0.008536	0.008548	0.004514	0.012795	100.00
-10	10	0.023569	0.023476	0.023215	0.020132	0.027194	0.015893	0.015828	0.015558	0.013621	0.018301	0.007677	0.007638	0.007766	0.003570	0.011975	99.99
-10	15	0.023491	0.023403	0.023375	0.019974	0.027178	0.017009	0.016948	0.016771	0.014504	0.019699	0.006482	0.006437	0.006234	0.002193	0.010833	99.87
-5	0	0.025474	0.025379	0.025127	0.021685	0.029361	0.023079	0.022992	0.022810	0.019619	0.026672	0.002395	0.002376	0.002136	-0.002652	0.007493	82.46
-5	5	0.025495	0.025400	0.025140	0.021785	0.029390	0.014940	0.014880	0.014737	0.012750	0.017261	0.010555	0.010496	0.010249	0.006260	0.014945	100.00
-5	10	0.025580	0.025477	0.025376	0.021941	0.029483	0.015908	0.015843	0.015721	0.013645	0.018314	0.009671	0.009615	0.009484	0.005334	0.014149	100.00
-5	15	0.025485	0.025380	0.025268	0.021793	0.029413	0.017030	0.016965	0.016714	0.014535	0.019679	0.008455	0.008407	0.008319	0.003978	0.013013	99.99
0	5	0.023011	0.022926	0.022590	0.019578	0.026655	0.014873	0.014818	0.014698	0.012676	0.017216	0.008137	0.008088	0.007916	0.004153	0.012300	100.00
0	10	0.023101	0.023014	0.022706	0.019702	0.026668	0.015860	0.015796	0.015642	0.013563	0.018273	0.007242	0.007200	0.007214	0.003126	0.011388	99.98
0	15	0.023018	0.022937	0.022718	0.019509	0.026624	0.016974	0.016915	0.016875	0.014425	0.019633	0.006043	0.006010	0.005949	0.001750	0.010270	99.80
5	10	0.014949	0.014888	0.014737	0.012774												

```
0.017241    0.015888      0.015823     0.015689      0.013602       0.018295
 -0.000939        -0.000925      -0.000847       -0.004153          0.002258
          28.14
       5       15    0.014901      0.014848    0.014789      0.012669
0.017193    0.017006      0.016942     0.016754      0.014426       0.019613
 -0.002104        -0.002092      -0.001902       -0.005424          0.001290
          10.71
      10       15    0.015892      0.015827    0.015811      0.013592
0.018276    0.017071      0.017006     0.016852      0.014580       0.019707
 -0.001178        -0.001173      -0.001275       -0.004601          0.002287
          24.90


****************************************************
Results for noise_window for additive noise in activation used in simulations:
[-5 ms  5 ms]; 10 random samples per segment.
****************************************************
Each row shows 50 simulation results for additive random noise; from top to
bottom the reference deterministic delay was set to -10 -5, 0, 5, 10, 15 ms
t_l_max_1 =     [5.1047920e-02   5.1031920e-02   5.0967920e-02   5.0975920e-02
5.1007920e-02   5.1247920e-02   5.1135920e-02   5.1143920e-02   5.1047920e-02
5.1191920e-02   5.1175920e-02   5.1239920e-02   5.0967920e-02   5.1143920e-02
5.1159920e-02   5.1079920e-02   5.1151920e-02   5.0823920e-02   5.1135920e-02
5.1039920e-02   5.1087920e-02   5.1103920e-02   5.1071920e-02   5.1039920e-02
5.1191920e-02   5.1015920e-02   5.1167920e-02   5.1047920e-02   5.0903920e-02
5.1127920e-02   5.0935920e-02   5.1215920e-02   5.0999920e-02   5.1055920e-02
5.0991920e-02   5.1087920e-02   5.1103920e-02   5.1087920e-02   5.1103920e-02
5.1015920e-02   5.1087920e-02   5.1215920e-02   5.1095920e-02   5.1055920e-02
5.1207920e-02   5.1079920e-02   5.1127920e-02   5.1111920e-02   5.1079920e-02
5.1175920e-02;
   5.2207920e-02   5.2263920e-02   5.2199920e-02   5.2183920e-02   5.2175920e-02
  5.2175920e-02   5.2151920e-02   5.2151920e-02   5.2183920e-02   5.2279920e-02
 5.2255920e-02   5.2103920e-02   5.2303920e-02   5.2071920e-02   5.2287920e-02
5.2239920e-02   5.2215920e-02   5.2119920e-02   5.2175920e-02   5.2287920e-02
5.2111920e-02   5.2175920e-02   5.2303920e-02   5.2247920e-02   5.2127920e-02
5.2119920e-02   5.2079920e-02   5.2231920e-02   5.2159920e-02   5.2287920e-02
5.2119920e-02   5.2183920e-02   5.2311920e-02   5.2063920e-02   5.2207920e-02
5.2151920e-02   5.2295920e-02   5.2087920e-02   5.2199920e-02   5.2159920e-02
5.2183920e-02   5.2143920e-02   5.2223920e-02   5.2287920e-02   5.2103920e-02
5.2239920e-02   5.2111920e-02   5.2183920e-02   5.2159920e-02   5.2143920e-02;
   5.4079920e-02   5.4023920e-02   5.3999920e-02   5.4039920e-02   5.4143920e-02
  5.3951920e-02   5.3983920e-02   5.4023920e-02   5.4087920e-02   5.4023920e-02
 5.3975920e-02   5.3967920e-02   5.3895920e-02   5.3951920e-02   5.4015920e-02
5.3983920e-02   5.3999920e-02   5.3903920e-02   5.3983920e-02   5.4039920e-02
5.4095920e-02   5.3919920e-02   5.4007920e-02   5.4031920e-02   5.4047920e-02
5.3895920e-02   5.3983920e-02   5.4015920e-02   5.4135920e-02   5.3991920e-02
5.3863920e-02   5.4023920e-02   5.3983920e-02   5.4047920e-02   5.3759920e-02
5.3879920e-02   5.3959920e-02   5.3887920e-02   5.3983920e-02   5.4079920e-02
5.4039920e-02   5.4023920e-02   5.4055920e-02   5.4031920e-02   5.4039920e-02
5.3935920e-02   5.4079920e-02   5.4087920e-02   5.3927920e-02   5.3999920e-02;
   5.6495920e-02   5.6495920e-02   5.6455920e-02   5.6663920e-02   5.6647920e-02
  5.6631920e-02   5.6567920e-02   5.6567920e-02   5.6639920e-02   5.6543920e-02
 5.6519920e-02   5.6607920e-02   5.6631920e-02   5.6575920e-02   5.6631920e-02
5.6639920e-02   5.6631920e-02   5.6679920e-02   5.6663920e-02   5.6719920e-02
```

```
5.6527920e-02   5.6543920e-02   5.6631920e-02   5.6575920e-02   5.6671920e-02
5.6663920e-02   5.6471920e-02   5.6695920e-02   5.6575920e-02   5.6663920e-02
5.6543920e-02   5.6719920e-02   5.6623920e-02   5.6631920e-02   5.6647920e-02
5.6543920e-02   5.6663920e-02   5.6463920e-02   5.6559920e-02   5.6631920e-02
5.6599920e-02   5.6607920e-02   5.6631920e-02   5.6647920e-02   5.6567920e-02
5.6567920e-02   5.6559920e-02   5.6575920e-02   5.6671920e-02   5.6695920e-02;
   5.9943920e-02   6.0031920e-02   5.9935920e-02   6.0159920e-02   5.9927920e-02
  5.9879920e-02   5.9983920e-02   6.0023920e-02   6.0031920e-02   5.9871920e-02
 6.0007920e-02   6.0079920e-02   5.9887920e-02   5.9959920e-02   6.0015920e-02
5.9903920e-02   5.9991920e-02   5.9975920e-02   5.9871920e-02   6.0111920e-02
6.0055920e-02   5.9959920e-02   6.0039920e-02   6.0095920e-02   5.9911920e-02
5.9847920e-02   6.0039920e-02   5.9967920e-02   5.9975920e-02   6.0023920e-02
5.9807920e-02   5.9999920e-02   5.9959920e-02   5.9967920e-02   5.9847920e-02
5.9943920e-02   5.9967920e-02   6.0063920e-02   5.9991920e-02   5.9863920e-02
6.0007920e-02   5.9887920e-02   5.9815920e-02   5.9951920e-02   6.0047920e-02
6.0159920e-02   6.0095920e-02   5.9951920e-02   5.9927920e-02   6.0023920e-02;
   6.3351920e-02   6.3335920e-02   6.3271920e-02   6.3399920e-02   6.3319920e-02
  6.3255920e-02   6.3311920e-02   6.3399920e-02   6.3247920e-02   6.3415920e-02
 6.3255920e-02   6.3367920e-02   6.3215920e-02   6.3295920e-02   6.3423920e-02
6.3287920e-02   6.3167920e-02   6.3287920e-02   6.3207920e-02   6.3335920e-02
6.3415920e-02   6.3415920e-02   6.3215920e-02   6.3295920e-02   6.3359920e-02
6.3239920e-02   6.3319920e-02   6.3199920e-02   6.3439920e-02   6.3391920e-02
6.3415920e-02   6.3271920e-02   6.3271920e-02   6.3327920e-02   6.3447920e-02
6.3463920e-02   6.3215920e-02   6.3231920e-02   6.3303920e-02   6.3263920e-02
6.3255920e-02   6.3367920e-02   6.3375920e-02   6.3367920e-02   6.3383920e-02
6.3215920e-02   6.3231920e-02   6.3303920e-02   6.3327920e-02   6.3207920e-02];
t_u_max_1 =     [3.9151920e-02   3.9119920e-02   3.9087920e-02   3.9079920e-02
3.9023920e-02   3.9271920e-02   3.9071920e-02   3.9231920e-02   3.8975920e-02
3.9207920e-02   3.9247920e-02   3.9463920e-02   3.8983920e-02   3.9255920e-02
3.9223920e-02   3.9119920e-02   3.9175920e-02   3.8863920e-02   3.9191920e-02
3.9095920e-02   3.9223920e-02   3.9167920e-02   3.9239920e-02   3.9207920e-02
3.9447920e-02   3.8959920e-02   3.9223920e-02   3.9191920e-02   3.8983920e-02
3.9191920e-02   3.9023920e-02   3.9263920e-02   3.9119920e-02   3.9167920e-02
3.9143920e-02   3.8999920e-02   3.9111920e-02   3.9055920e-02   3.9199920e-02
3.9095920e-02   3.9127920e-02   3.9247920e-02   3.9215920e-02   3.9111920e-02
3.9335920e-02   3.9087920e-02   3.9327920e-02   3.9087920e-02   3.9079920e-02
3.9239920e-02;
   4.0655920e-02   4.0695920e-02   4.0655920e-02   4.0631920e-02   4.0663920e-02
  4.0559920e-02   4.0535920e-02   4.0695920e-02   4.0711920e-02   4.0743920e-02
 4.0735920e-02   4.0527920e-02   4.0767920e-02   4.0631920e-02   4.0759920e-02
4.0679920e-02   4.0743920e-02   4.0591920e-02   4.0663920e-02   4.0767920e-02
4.0615920e-02   4.0623920e-02   4.0711920e-02   4.0599920e-02   4.0623920e-02
4.0559920e-02   4.0583920e-02   4.0767920e-02   4.0471920e-02   4.0615920e-02
4.0607920e-02   4.0639920e-02   4.0687920e-02   4.0535920e-02   4.0727920e-02
4.0511920e-02   4.0855920e-02   4.0575920e-02   4.0599920e-02   4.0639920e-02
4.0695920e-02   4.0687920e-02   4.0679920e-02   4.0695920e-02   4.0559920e-02
4.0631920e-02   4.0615920e-02   4.0695920e-02   4.0583920e-02   4.0711920e-02;
   4.2759920e-02   4.2703920e-02   4.2671920e-02   4.2735920e-02   4.2815920e-02
  4.2639920e-02   4.2671920e-02   4.2711920e-02   4.2783920e-02   4.2687920e-02
 4.2671920e-02   4.2663920e-02   4.2591920e-02   4.2639920e-02   4.2719920e-02
4.2671920e-02   4.2671920e-02   4.2583920e-02   4.2647920e-02   4.2695920e-02
4.2767920e-02   4.2583920e-02   4.2703920e-02   4.2703920e-02   4.2735920e-02
4.2599920e-02   4.2655920e-02   4.2695920e-02   4.2823920e-02   4.2695920e-02
4.2559920e-02   4.2711920e-02   4.2671920e-02   4.2719920e-02   4.2455920e-02
```

4.2567920e-02   4.2639920e-02   4.2559920e-02   4.2647920e-02   4.2743920e-02
4.2719920e-02   4.2727920e-02   4.2743920e-02   4.2727920e-02   4.2727920e-02
4.2639920e-02   4.2743920e-02   4.2751920e-02   4.2623920e-02   4.2671920e-02;
4.5335920e-02   4.5335920e-02   4.5311920e-02   4.5479920e-02   4.5503920e-02
4.5495920e-02   4.5423920e-02   4.5375920e-02   4.5487920e-02   4.5407920e-02
4.5351920e-02   4.5447920e-02   4.5495920e-02   4.5415920e-02   4.5439920e-02
4.5479920e-02   4.5447920e-02   4.5511920e-02   4.5503920e-02   4.5591920e-02
4.5375920e-02   4.5391920e-02   4.5479920e-02   4.5399920e-02   4.5503920e-02
4.5511920e-02   4.5319920e-02   4.5583920e-02   4.5423920e-02   4.5511920e-02
4.5383920e-02   4.5559920e-02   4.5463920e-02   4.5471920e-02   4.5495920e-02
4.5391920e-02   4.5487920e-02   4.5319920e-02   4.5423920e-02   4.5479920e-02
4.5447920e-02   4.5439920e-02   4.5455920e-02   4.5439920e-02   4.5383920e-02
4.5391920e-02   4.5407920e-02   4.5415920e-02   4.5511920e-02   4.5551920e-02;
4.8855920e-02   4.8927920e-02   4.8847920e-02   4.9047920e-02   4.8823920e-02
4.8767920e-02   4.8887920e-02   4.8927920e-02   4.8919920e-02   4.8775920e-02
4.8911920e-02   4.8983920e-02   4.8791920e-02   4.8847920e-02   4.8919920e-02
4.8807920e-02   4.8895920e-02   4.8871920e-02   4.8791920e-02   4.9015920e-02
4.8951920e-02   4.8871920e-02   4.8943920e-02   4.8983920e-02   4.8799920e-02
4.8743920e-02   4.8935920e-02   4.8871920e-02   4.8871920e-02   4.8911920e-02
4.8711920e-02   4.8895920e-02   4.8855920e-02   4.8871920e-02   4.8751920e-02
4.8847920e-02   4.8879920e-02   4.8943920e-02   4.8895920e-02   4.8767920e-02
4.8911920e-02   4.8783920e-02   4.8719920e-02   4.8863920e-02   4.8951920e-02
4.9047920e-02   4.8983920e-02   4.8847920e-02   4.8823920e-02   4.8911920e-02;
5.2159920e-02   5.2135920e-02   5.2087920e-02   5.2215920e-02   5.2143920e-02
5.2071920e-02   5.2143920e-02   5.2215920e-02   5.2055920e-02   5.2231920e-02
5.2047920e-02   5.2175920e-02   5.2031920e-02   5.2103920e-02   5.2247920e-02
5.2103920e-02   5.1983920e-02   5.2103920e-02   5.2023920e-02   5.2143920e-02
5.2223920e-02   5.2231920e-02   5.2031920e-02   5.2103920e-02   5.2175920e-02
5.2063920e-02   5.2135920e-02   5.2023920e-02   5.2239920e-02   5.2199920e-02
5.2223920e-02   5.2079920e-02   5.2087920e-02   5.2151920e-02   5.2255920e-02
5.2311920e-02   5.2055920e-02   5.2039920e-02   5.2111920e-02   5.2079920e-02
5.2079920e-02   5.2183920e-02   5.2191920e-02   5.2175920e-02   5.2191920e-02
5.2023920e-02   5.2015920e-02   5.2143920e-02   5.2135920e-02   5.2023920e-02];
l_max_1 =   [8.5189934e-02   8.5191563e-02   8.5203934e-02   8.5191300e-02
8.5194388e-02   8.5170643e-02   8.5182804e-02   8.5190653e-02   8.5188624e-02
8.5193390e-02   8.5176703e-02   8.5196132e-02   8.5190410e-02   8.5184976e-02
8.5190023e-02   8.5206842e-02   8.5181506e-02   8.5211431e-02   8.5192905e-02
8.5180707e-02   8.5199074e-02   8.5187541e-02   8.5197461e-02   8.5211255e-02
8.5201652e-02   8.5182976e-02   8.5188425e-02   8.5194232e-02   8.5202413e-02
8.5189296e-02   8.5200533e-02   8.5179822e-02   8.5199994e-02   8.5177126e-02
8.5198017e-02   8.5169602e-02   8.5196545e-02   8.5181688e-02   8.5191684e-02
8.5204046e-02   8.5188795e-02   8.5184907e-02   8.5199654e-02   8.5190340e-02
8.5195865e-02   8.5196885e-02   8.5199733e-02   8.5194321e-02   8.5184810e-02
8.5180286e-02;
8.5425788e-02   8.5415346e-02   8.5422844e-02   8.5425665e-02   8.5418211e-02
8.5401649e-02   8.5416038e-02   8.5436155e-02   8.5431791e-02   8.5427926e-02
8.5421176e-02   8.5432725e-02   8.5410805e-02   8.5445540e-02   8.5428974e-02
8.5407568e-02   8.5437318e-02   8.5412702e-02   8.5420975e-02   8.5423790e-02
8.5419118e-02   8.5418001e-02   8.5405612e-02   8.5401515e-02   8.5430010e-02
8.5421941e-02   8.5438525e-02   8.5444354e-02   8.5390684e-02   8.5403381e-02
8.5420241e-02   8.5428381e-02   8.5414673e-02   8.5429655e-02   8.5430568e-02
8.5407447e-02   8.5419817e-02   8.5434335e-02   8.5426949e-02   8.5418590e-02
8.5425258e-02   8.5435572e-02   8.5417925e-02   8.5411445e-02   8.5419365e-02
8.5413005e-02   8.5428679e-02   8.5430505e-02   8.5421304e-02   8.5440856e-02;

```
   8.5548319e-02   8.5552289e-02   8.5548817e-02   8.5553661e-02   8.5551571e-02
   8.5554111e-02   8.5557424e-02   8.5542662e-02   8.5557036e-02   8.5554878e-02
   8.5554471e-02   8.5544427e-02   8.5546060e-02   8.5555041e-02   8.5557503e-02
8.5556331e-02   8.5543113e-02   8.5542225e-02   8.5544770e-02   8.5542838e-02
8.5535906e-02   8.5549692e-02   8.5551410e-02   8.5552815e-02   8.5559063e-02
8.5544756e-02   8.5549479e-02   8.5543009e-02   8.5548012e-02   8.5556990e-02
8.5552463e-02   8.5554397e-02   8.5554109e-02   8.5550576e-02   8.5556928e-02
8.5548497e-02   8.5546063e-02   8.5545062e-02   8.5539791e-02   8.5545190e-02
8.5553044e-02   8.5549794e-02   8.5557900e-02   8.5553451e-02   8.5541612e-02
8.5557269e-02   8.5542653e-02   8.5550476e-02   8.5551349e-02   8.5550047e-02;
   8.5435638e-02   8.5428084e-02   8.5417704e-02   8.5433853e-02   8.5428156e-02
   8.5423538e-02   8.5413126e-02   8.5429340e-02   8.5418134e-02   8.5396089e-02
   8.5431906e-02   8.5423077e-02   8.5398076e-02   8.5421234e-02   8.5414596e-02
8.5417716e-02   8.5422819e-02   8.5417329e-02   8.5411000e-02   8.5400006e-02
8.5420318e-02   8.5418333e-02   8.5416522e-02   8.5428796e-02   8.5434322e-02
8.5409527e-02   8.5421888e-02   8.5412708e-02   8.5418073e-02   8.5424216e-02
8.5427243e-02   8.5428143e-02   8.5398394e-02   8.5403397e-02   8.5417951e-02
8.5424665e-02   8.5408088e-02   8.5436706e-02   8.5417896e-02   8.5407657e-02
8.5417084e-02   8.5414288e-02   8.5432106e-02   8.5423899e-02   8.5422611e-02
8.5442510e-02   8.5426652e-02   8.5424213e-02   8.5418819e-02   8.5404297e-02;
   8.5133729e-02   8.5116315e-02   8.5158235e-02   8.5118329e-02   8.5128566e-02
   8.5128658e-02   8.5097293e-02   8.5111779e-02   8.5121119e-02   8.5135284e-02
   8.5110301e-02   8.5110837e-02   8.5136366e-02   8.5121958e-02   8.5113379e-02
8.5144430e-02   8.5136544e-02   8.5125483e-02   8.5170569e-02   8.5120953e-02
8.5113343e-02   8.5144741e-02   8.5140526e-02   8.5092597e-02   8.5115807e-02
8.5152177e-02   8.5102234e-02   8.5132650e-02   8.5117099e-02   8.5106200e-02
8.5146223e-02   8.5112628e-02   8.5125144e-02   8.5111645e-02   8.5149777e-02
8.5129190e-02   8.5127248e-02   8.5092035e-02   8.5142816e-02   8.5136329e-02
8.5143982e-02   8.5128313e-02   8.5118396e-02   8.5118258e-02   8.5127608e-02
8.5105644e-02   8.5105456e-02   8.5103888e-02   8.5107591e-02   8.5117161e-02;
   8.4862473e-02   8.4860413e-02   8.4878867e-02   8.4876902e-02   8.4875199e-02
   8.4862856e-02   8.4893890e-02   8.4868613e-02   8.4875071e-02   8.4875228e-02
   8.4864057e-02   8.4863106e-02   8.4869830e-02   8.4884032e-02   8.4879902e-02
8.4866194e-02   8.4889045e-02   8.4877510e-02   8.4874625e-02   8.4876259e-02
8.4855590e-02   8.4861683e-02   8.4881000e-02   8.4861214e-02   8.4875395e-02
8.4885204e-02   8.4888734e-02   8.4881431e-02   8.4872028e-02   8.4862873e-02
8.4869581e-02   8.4868495e-02   8.4888479e-02   8.4888685e-02   8.4856188e-02
8.4912770e-02   8.4909235e-02   8.4866005e-02   8.4875212e-02   8.4866058e-02
8.4879999e-02   8.4873310e-02   8.4870578e-02   8.4880361e-02   8.4867493e-02
8.4879890e-02   8.4852965e-02   8.4892571e-02   8.4867947e-02   8.4889831e-02];
u_max_1 =       [1.8628861e+00   1.8589719e+00   1.8693799e+00   1.8616466e+00
1.8665915e+00   1.8464577e+00   1.8597290e+00   1.8600221e+00   1.8675333e+00
1.8647007e+00   1.8497599e+00   1.8559352e+00   1.8630553e+00   1.8543949e+00
1.8607754e+00   1.8733134e+00   1.8564264e+00   1.8863800e+00   1.8591428e+00
1.8550548e+00   1.8643063e+00   1.8598507e+00   1.8617879e+00   1.8743655e+00
1.8609810e+00   1.8580617e+00   1.8576976e+00   1.8621947e+00   1.8716222e+00
1.8575761e+00   1.8703377e+00   1.8542561e+00   1.8683647e+00   1.8470643e+00
1.8642432e+00   1.8522845e+00   1.8725500e+00   1.8584606e+00   1.8599061e+00
1.8764586e+00   1.8614105e+00   1.8625541e+00   1.8628567e+00   1.8589602e+00
1.8641874e+00   1.8680832e+00   1.8604572e+00   1.8685527e+00   1.8563259e+00
1.8519006e+00;
   2.0538080e+00   2.0459702e+00   2.0558985e+00   2.0608620e+00   2.0438374e+00
   2.0333611e+00   2.0526505e+00   2.0610618e+00   2.0561412e+00   2.0533317e+00
   2.0490507e+00   2.0651242e+00   2.0475939e+00   2.0684227e+00   2.0535482e+00
```

```
2.0452544e+00   2.0598128e+00   2.0448688e+00   2.0468032e+00   2.0500200e+00
2.0521876e+00   2.0493561e+00   2.0419908e+00   2.0395655e+00   2.0589378e+00
2.0551156e+00   2.0648113e+00   2.0627555e+00   2.0353526e+00   2.0455229e+00
2.0522638e+00   2.0582553e+00   2.0509145e+00   2.0589505e+00   2.0548630e+00
2.0470190e+00   2.0422295e+00   2.0619367e+00   2.0627813e+00   2.0498325e+00
2.0533063e+00   2.0572713e+00   2.0498858e+00   2.0441641e+00   2.0520028e+00
2.0511482e+00   2.0577330e+00   2.0603234e+00   2.0558339e+00   2.0626856e+00;
    2.2091846e+00   2.2187839e+00   2.2129966e+00   2.2155585e+00   2.2161593e+00
  2.2251825e+00   2.2181460e+00   2.2150014e+00   2.2141330e+00   2.2255004e+00
 2.2153692e+00   2.2152125e+00   2.2242230e+00   2.2118011e+00   2.2238524e+00
2.2179369e+00   2.2153298e+00   2.2128572e+00   2.2120618e+00   2.2075180e+00
2.2076355e+00   2.2164251e+00   2.2169941e+00   2.2202886e+00   2.2148594e+00
2.2168545e+00   2.2130786e+00   2.2136038e+00   2.2094987e+00   2.2240886e+00
2.2208261e+00   2.2133035e+00   2.2244163e+00   2.2168000e+00   2.2207029e+00
2.2150091e+00   2.2026871e+00   2.2123710e+00   2.2129022e+00   2.2111887e+00
2.2211201e+00   2.2178584e+00   2.2202521e+00   2.2175896e+00   2.2118406e+00
2.2274540e+00   2.2127935e+00   2.2133027e+00   2.2260924e+00   2.2125175e+00;
    2.2628194e+00   2.2613919e+00   2.2617592e+00   2.2597666e+00   2.2643848e+00
  2.2669494e+00   2.2593425e+00   2.2540510e+00   2.2569492e+00   2.2545838e+00
 2.2561036e+00   2.2600152e+00   2.2546092e+00   2.2587962e+00   2.2480845e+00
2.2615768e+00   2.2577285e+00   2.2536002e+00   2.2576799e+00   2.2515418e+00
2.2610570e+00   2.2546458e+00   2.2576909e+00   2.2540894e+00   2.2616338e+00
2.2574122e+00   2.2571129e+00   2.2584999e+00   2.2594581e+00   2.2617248e+00
2.2605175e+00   2.2603532e+00   2.2469094e+00   2.2530587e+00   2.2568953e+00
2.2613006e+00   2.2515602e+00   2.2659143e+00   2.2594705e+00   2.2569704e+00
2.2568953e+00   2.2583455e+00   2.2583173e+00   2.2457911e+00   2.2541916e+00
2.2629745e+00   2.2589304e+00   2.2594534e+00   2.2557805e+00   2.2584656e+00;
    2.1796455e+00   2.1747949e+00   2.1885980e+00   2.1702290e+00   2.1769423e+00
  2.1777070e+00   2.1664155e+00   2.1704207e+00   2.1749217e+00   2.1811758e+00
 2.1709809e+00   2.1718603e+00   2.1822038e+00   2.1722547e+00   2.1720595e+00
2.1836214e+00   2.1813896e+00   2.1763604e+00   2.1951213e+00   2.1749132e+00
2.1714257e+00   2.1840416e+00   2.1814253e+00   2.1634843e+00   2.1729522e+00
2.1826059e+00   2.1697238e+00   2.1797048e+00   2.1729583e+00   2.1678236e+00
2.1851140e+00   2.1691989e+00   2.1751209e+00   2.1721077e+00   2.1859362e+00
2.1777891e+00   2.1762735e+00   2.1645838e+00   2.1833211e+00   2.1808921e+00
2.1845235e+00   2.1779840e+00   2.1763755e+00   2.1740019e+00   2.1785982e+00
2.1678981e+00   2.1706616e+00   2.1687629e+00   2.1699994e+00   2.1725830e+00;
    2.0594866e+00   2.0575674e+00   2.0661111e+00   2.0658708e+00   2.0651689e+00
  2.0586550e+00   2.0743536e+00   2.0620058e+00   2.0651307e+00   2.0651032e+00
 2.0603034e+00   2.0594965e+00   2.0628057e+00   2.0697007e+00   2.0673029e+00
2.0614959e+00   2.0726635e+00   2.0664018e+00   2.0645510e+00   2.0657181e+00
2.0557408e+00   2.0594668e+00   2.0684421e+00   2.0582908e+00   2.0647699e+00
2.0691125e+00   2.0725992e+00   2.0681191e+00   2.0635386e+00   2.0591387e+00
2.0629774e+00   2.0626186e+00   2.0706027e+00   2.0703601e+00   2.0555104e+00
2.0818111e+00   2.0813135e+00   2.0611796e+00   2.0655110e+00   2.0609278e+00
2.0665080e+00   2.0645994e+00   2.0630818e+00   2.0678203e+00   2.0611874e+00
2.0679812e+00   2.0545707e+00   2.0728023e+00   2.0604376e+00   2.0722921e+00];
t_l_max_2 = [5.1151920e-02   5.0975920e-02   5.1055920e-02   5.0991920e-02
5.1167920e-02   5.1039920e-02   5.1023920e-02   5.1247920e-02   5.1151920e-02
5.1023920e-02   5.1039920e-02   5.1071920e-02   5.1023920e-02   5.1071920e-02
5.1023920e-02   5.1119920e-02   5.0991920e-02   5.1007920e-02   5.0943920e-02
5.1071920e-02   5.0927920e-02   5.1087920e-02   5.1167920e-02   5.1231920e-02
5.0959920e-02   5.1023920e-02   5.1007920e-02   5.1215920e-02   5.0943920e-02
5.1295920e-02   5.1215920e-02   5.1039920e-02   5.1007920e-02   5.1151920e-02
```

```
5.1039920e-02   5.1135920e-02   5.1151920e-02   5.1071920e-02   5.1183920e-02
5.0831920e-02   5.1151920e-02   5.1039920e-02   5.1055920e-02   5.1103920e-02
5.1103920e-02   5.1167920e-02   5.1103920e-02   5.1055920e-02   5.1199920e-02
5.1023920e-02;
   5.2303920e-02   5.2127920e-02   5.2143920e-02   5.2079920e-02   5.2175920e-02
  5.2159920e-02   5.2111920e-02   5.2191920e-02   5.2191920e-02   5.2127920e-02
 5.2191920e-02   5.2079920e-02   5.2207920e-02   5.2063920e-02   5.2095920e-02
5.2223920e-02   5.2271920e-02   5.2303920e-02   5.2159920e-02   5.2287920e-02
5.2399920e-02   5.2271920e-02   5.2239920e-02   5.2079920e-02   5.2111920e-02
5.2159920e-02   5.2255920e-02   5.2143920e-02   5.2239920e-02   5.2143920e-02
5.2015920e-02   5.2143920e-02   5.2143920e-02   5.2191920e-02   5.2255920e-02
5.2303920e-02   5.2239920e-02   5.2287920e-02   5.2255920e-02   5.2095920e-02
5.2111920e-02   5.2143920e-02   5.2191920e-02   5.2111920e-02   5.2159920e-02
5.2175920e-02   5.2271920e-02   5.2191920e-02   5.2223920e-02   5.2335920e-02;
   5.3903920e-02   5.3919920e-02   5.3951920e-02   5.3919920e-02   5.3951920e-02
  5.3967920e-02   5.3999920e-02   5.4015920e-02   5.3983920e-02   5.4031920e-02
 5.3935920e-02   5.4079920e-02   5.4079920e-02   5.3967920e-02   5.4063920e-02
5.4095920e-02   5.3919920e-02   5.3903920e-02   5.4015920e-02   5.3919920e-02
5.3999920e-02   5.4015920e-02   5.4015920e-02   5.3951920e-02   5.3983920e-02
5.4079920e-02   5.3999920e-02   5.3983920e-02   5.4015920e-02   5.4047920e-02
5.4015920e-02   5.3951920e-02   5.3855920e-02   5.4127920e-02   5.3871920e-02
5.4127920e-02   5.4063920e-02   5.4079920e-02   5.3903920e-02   5.4031920e-02
5.3919920e-02   5.3919920e-02   5.3983920e-02   5.3967920e-02   5.4063920e-02
5.4031920e-02   5.4015920e-02   5.4095920e-02   5.3871920e-02   5.4079920e-02;
   5.6591920e-02   5.6607920e-02   5.6767920e-02   5.6687920e-02   5.6719920e-02
  5.6527920e-02   5.6639920e-02   5.6639920e-02   5.6639920e-02   5.6591920e-02
 5.6591920e-02   5.6639920e-02   5.6719920e-02   5.6703920e-02   5.6735920e-02
5.6719920e-02   5.6495920e-02   5.6671920e-02   5.6655920e-02   5.6751920e-02
5.6687920e-02   5.6575920e-02   5.6607920e-02   5.6703920e-02   5.6607920e-02
5.6591920e-02   5.6607920e-02   5.6639920e-02   5.6543920e-02   5.6703920e-02
5.6671920e-02   5.6607920e-02   5.6575920e-02   5.6815920e-02   5.6591920e-02
5.6687920e-02   5.6591920e-02   5.6607920e-02   5.6639920e-02   5.6607920e-02
5.6543920e-02   5.6607920e-02   5.6575920e-02   5.6671920e-02   5.6575920e-02
5.6703920e-02   5.6607920e-02   5.6783920e-02   5.6591920e-02   5.6639920e-02;
   6.0095920e-02   5.9903920e-02   5.9871920e-02   5.9983920e-02   6.0111920e-02
  5.9791920e-02   5.9983920e-02   5.9967920e-02   6.0111920e-02   5.9903920e-02
 6.0047920e-02   6.0207920e-02   6.0143920e-02   6.0063920e-02   6.0175920e-02
5.9855920e-02   6.0063920e-02   6.0047920e-02   6.0031920e-02   6.0047920e-02
5.9951920e-02   5.9919920e-02   6.0063920e-02   5.9999920e-02   5.9983920e-02
5.9967920e-02   6.0047920e-02   5.9983920e-02   6.0095920e-02   6.0047920e-02
5.9983920e-02   5.9999920e-02   6.0111920e-02   6.0063920e-02   6.0031920e-02
5.9807920e-02   6.0079920e-02   6.0047920e-02   6.0015920e-02   6.0031920e-02
6.0063920e-02   6.0063920e-02   6.0175920e-02   6.0015920e-02   6.0015920e-02
5.9983920e-02   6.0063920e-02   6.0079920e-02   5.9887920e-02   6.0063920e-02;
   6.3151920e-02   6.3311920e-02   6.3391920e-02   6.3279920e-02   6.3263920e-02
  6.3263920e-02   6.3391920e-02   6.3375920e-02   6.3295920e-02   6.3167920e-02
 6.3167920e-02   6.3375920e-02   6.3263920e-02   6.3311920e-02   6.3279920e-02
6.3343920e-02   6.3343920e-02   6.3343920e-02   6.3343920e-02   6.3375920e-02
6.3279920e-02   6.3295920e-02   6.3279920e-02   6.3375920e-02   6.3231920e-02
6.3279920e-02   6.3167920e-02   6.3327920e-02   6.3279920e-02   6.3311920e-02
6.3311920e-02   6.3407920e-02   6.3231920e-02   6.3279920e-02   6.3247920e-02
6.3295920e-02   6.3455920e-02   6.3327920e-02   6.3215920e-02   6.3423920e-02
6.3375920e-02   6.3199920e-02   6.3407920e-02   6.3295920e-02   6.3471920e-02
6.3311920e-02   6.3359920e-02   6.3359920e-02   6.3295920e-02   6.3199920e-02];
```

```
t_u_max_2 = [3.9199920e-02   3.8959920e-02   3.9135920e-02   3.8975920e-02
3.9247920e-02   3.9151920e-02   3.9199920e-02   3.9375920e-02   3.9183920e-02
3.8975920e-02   3.8959920e-02   3.9135920e-02   3.9167920e-02   3.9231920e-02
3.9231920e-02   3.9119920e-02   3.9199920e-02   3.9055920e-02   3.8895920e-02
3.9071920e-02   3.8879920e-02   3.9039920e-02   3.9247920e-02   3.9391920e-02
3.8895920e-02   3.9135920e-02   3.9039920e-02   3.9407920e-02   3.9055920e-02
3.9359920e-02   3.9311920e-02   3.9151920e-02   3.9087920e-02   3.9023920e-02
3.8991920e-02   3.9103920e-02   3.9199920e-02   3.8959920e-02   3.9231920e-02
3.8815920e-02   3.9311920e-02   3.9071920e-02   3.9151920e-02   3.9103920e-02
3.9263920e-02   3.9295920e-02   3.9135920e-02   3.9135920e-02   3.9343920e-02
3.9087920e-02;
   4.0799920e-02   4.0623920e-02   4.0607920e-02   4.0543920e-02   4.0687920e-02
  4.0559920e-02   4.0623920e-02   4.0655920e-02   4.0671920e-02   4.0591920e-02
 4.0639920e-02   4.0559920e-02   4.0703920e-02   4.0559920e-02   4.0623920e-02
4.0639920e-02   4.0799920e-02   4.0703920e-02   4.0575920e-02   4.0751920e-02
4.0847920e-02   4.0591920e-02   4.0623920e-02   4.0511920e-02   4.0623920e-02
4.0639920e-02   4.0719920e-02   4.0607920e-02   4.0735920e-02   4.0671920e-02
4.0495920e-02   4.0639920e-02   4.0623920e-02   4.0655920e-02   4.0719920e-02
4.0751920e-02   4.0703920e-02   4.0767920e-02   4.0591920e-02   4.0543920e-02
4.0655920e-02   4.0623920e-02   4.0639920e-02   4.0559920e-02   4.0639920e-02
4.0607920e-02   4.0735920e-02   4.0703920e-02   4.0751920e-02   4.0799920e-02;
   4.2591920e-02   4.2607920e-02   4.2639920e-02   4.2607920e-02   4.2639920e-02
  4.2655920e-02   4.2671920e-02   4.2671920e-02   4.2655920e-02   4.2719920e-02
 4.2607920e-02   4.2735920e-02   4.2751920e-02   4.2655920e-02   4.2751920e-02
4.2799920e-02   4.2607920e-02   4.2591920e-02   4.2687920e-02   4.2623920e-02
4.2687920e-02   4.2719920e-02   4.2703920e-02   4.2639920e-02   4.2671920e-02
4.2767920e-02   4.2687920e-02   4.2671920e-02   4.2703920e-02   4.2719920e-02
4.2703920e-02   4.2655920e-02   4.2527920e-02   4.2815920e-02   4.2575920e-02
4.2815920e-02   4.2735920e-02   4.2783920e-02   4.2591920e-02   4.2719920e-02
4.2607920e-02   4.2623920e-02   4.2655920e-02   4.2655920e-02   4.2751920e-02
4.2703920e-02   4.2703920e-02   4.2783920e-02   4.2543920e-02   4.2767920e-02;
   4.5407920e-02   4.5423920e-02   4.5615920e-02   4.5551920e-02   4.5535920e-02
  4.5343920e-02   4.5471920e-02   4.5439920e-02   4.5439920e-02   4.5423920e-02
 4.5423920e-02   4.5487920e-02   4.5567920e-02   4.5567920e-02   4.5551920e-02
4.5535920e-02   4.5327920e-02   4.5519920e-02   4.5487920e-02   4.5615920e-02
4.5551920e-02   4.5407920e-02   4.5439920e-02   4.5551920e-02   4.5423920e-02
4.5423920e-02   4.5439920e-02   4.5455920e-02   4.5359920e-02   4.5519920e-02
4.5487920e-02   4.5439920e-02   4.5375920e-02   4.5631920e-02   4.5455920e-02
4.5535920e-02   4.5471920e-02   4.5439920e-02   4.5455920e-02   4.5455920e-02
4.5391920e-02   4.5439920e-02   4.5423920e-02   4.5503920e-02   4.5439920e-02
4.5551920e-02   4.5471920e-02   4.5631920e-02   4.5439920e-02   4.5471920e-02;
   4.8991920e-02   4.8815920e-02   4.8767920e-02   4.8879920e-02   4.9007920e-02
  4.8703920e-02   4.8879920e-02   4.8863920e-02   4.9007920e-02   4.8799920e-02
 4.8943920e-02   4.9103920e-02   4.9039920e-02   4.8959920e-02   4.9071920e-02
4.8767920e-02   4.8975920e-02   4.8943920e-02   4.8943920e-02   4.8927920e-02
4.8863920e-02   4.8831920e-02   4.8959920e-02   4.8895920e-02   4.8879920e-02
4.8863920e-02   4.8943920e-02   4.8895920e-02   4.8975920e-02   4.8959920e-02
4.8863920e-02   4.8895920e-02   4.9007920e-02   4.8943920e-02   4.8927920e-02
4.8703920e-02   4.8975920e-02   4.8927920e-02   4.8911920e-02   4.8943920e-02
4.8959920e-02   4.8959920e-02   4.9071920e-02   4.8927920e-02   4.8911920e-02
4.8879920e-02   4.8959920e-02   4.8975920e-02   4.8799920e-02   4.8959920e-02;
   5.1967920e-02   5.2127920e-02   5.2223920e-02   5.2095920e-02   5.2063920e-02
  5.2079920e-02   5.2207920e-02   5.2191920e-02   5.2111920e-02   5.1983920e-02
 5.1983920e-02   5.2207920e-02   5.2079920e-02   5.2143920e-02   5.2095920e-02
```

```
5.2191920e-02   5.2143920e-02   5.2143920e-02   5.2175920e-02   5.2191920e-02
5.2095920e-02   5.2127920e-02   5.2095920e-02   5.2175920e-02   5.2063920e-02
5.2095920e-02   5.1983920e-02   5.2143920e-02   5.2079920e-02   5.2143920e-02
5.2111920e-02   5.2239920e-02   5.2031920e-02   5.2079920e-02   5.2063920e-02
5.2095920e-02   5.2271920e-02   5.2143920e-02   5.2031920e-02   5.2239920e-02
5.2159920e-02   5.2015920e-02   5.2223920e-02   5.2111920e-02   5.2271920e-02
5.2111920e-02   5.2191920e-02   5.2159920e-02   5.2111920e-02   5.1999920e-02];
l_max_2 =   [8.5173380e-02   8.5194511e-02   8.5175661e-02   8.5183915e-02
8.5186848e-02   8.5194447e-02   8.5205933e-02   8.5175805e-02   8.5176895e-02
8.5182291e-02   8.5169972e-02   8.5184070e-02   8.5204109e-02   8.5215275e-02
8.5222068e-02   8.5186140e-02   8.5207664e-02   8.5188013e-02   8.5188607e-02
8.5169539e-02   8.5177154e-02   8.5169298e-02   8.5194997e-02   8.5200398e-02
8.5193650e-02   8.5198336e-02   8.5187784e-02   8.5189316e-02   8.5211892e-02
8.5170359e-02   8.5181122e-02   8.5189446e-02   8.5196488e-02   8.5178764e-02
8.5189903e-02   8.5192371e-02   8.5165524e-02   8.5172829e-02   8.5187082e-02
8.5199949e-02   8.5203867e-02   8.5184122e-02   8.5197249e-02   8.5185284e-02
8.5206591e-02   8.5197186e-02   8.5189639e-02   8.5194730e-02   8.5184309e-02
8.5194498e-02;
   8.5421488e-02   8.5434273e-02   8.5429363e-02   8.5430130e-02   8.5433447e-02
  8.5408472e-02   8.5432474e-02   8.5429309e-02   8.5435264e-02   8.5429560e-02
 8.5424889e-02   8.5442128e-02   8.5432797e-02   8.5437152e-02   8.5449761e-02
8.5404113e-02   8.5424437e-02   8.5408177e-02   8.5410142e-02   8.5417102e-02
8.5405362e-02   8.5401697e-02   8.5406888e-02   8.5417724e-02   8.5434759e-02
8.5432122e-02   8.5432140e-02   8.5441359e-02   8.5423619e-02   8.5432529e-02
8.5446983e-02   8.5433760e-02   8.5429876e-02   8.5420697e-02   8.5417703e-02
8.5407618e-02   8.5417827e-02   8.5421218e-02   8.5407279e-02   8.5425795e-02
8.5447386e-02   8.5412375e-02   8.5425969e-02   8.5433724e-02   8.5434495e-02
8.5420154e-02   8.5411822e-02   8.5433369e-02   8.5432208e-02   8.5414995e-02;
   8.5538774e-02   8.5559532e-02   8.5556720e-02   8.5547582e-02   8.5555936e-02
  8.5557649e-02   8.5537801e-02   8.5544839e-02   8.5545504e-02   8.5529257e-02
 8.5539221e-02   8.5544022e-02   8.5548123e-02   8.5551286e-02   8.5548581e-02
8.5548227e-02   8.5539891e-02   8.5550592e-02   8.5553855e-02   8.5552520e-02
8.5538623e-02   8.5543359e-02   8.5555270e-02   8.5550151e-02   8.5554033e-02
8.5544791e-02   8.5544399e-02   8.5552517e-02   8.5550117e-02   8.5554374e-02
8.5546370e-02   8.5558396e-02   8.5558987e-02   8.5550309e-02   8.5546680e-02
8.5543461e-02   8.5529325e-02   8.5556427e-02   8.5544150e-02   8.5556816e-02
8.5555383e-02   8.5557288e-02   8.5538500e-02   8.5551930e-02   8.5549558e-02
8.5545181e-02   8.5555630e-02   8.5544997e-02   8.5550601e-02   8.5542489e-02;
   8.5434729e-02   8.5431891e-02   8.5406841e-02   8.5414988e-02   8.5410513e-02
  8.5429937e-02   8.5419846e-02   8.5403492e-02   8.5423089e-02   8.5417744e-02
 8.5443922e-02   8.5428021e-02   8.5413453e-02   8.5400275e-02   8.5425747e-02
8.5419731e-02   8.5433885e-02   8.5406220e-02   8.5409403e-02   8.5410872e-02
8.5411432e-02   8.5430193e-02   8.5408509e-02   8.5404526e-02   8.5430078e-02
8.5423120e-02   8.5441818e-02   8.5428305e-02   8.5421392e-02   8.5420694e-02
8.5425707e-02   8.5438088e-02   8.5448462e-02   8.5399712e-02   8.5421515e-02
8.5404629e-02   8.5418981e-02   8.5414617e-02   8.5429959e-02   8.5428791e-02
8.5417468e-02   8.5397030e-02   8.5421436e-02   8.5430307e-02   8.5405398e-02
8.5414025e-02   8.5412453e-02   8.5402366e-02   8.5432964e-02   8.5457201e-02;
   8.5144728e-02   8.5124126e-02   8.5133778e-02   8.5117615e-02   8.5116535e-02
  8.5136390e-02   8.5130544e-02   8.5115870e-02   8.5125729e-02   8.5124546e-02
 8.5123738e-02   8.5092085e-02   8.5119040e-02   8.5103308e-02   8.5103734e-02
8.5142628e-02   8.5118925e-02   8.5122593e-02   8.5120150e-02   8.5124609e-02
8.5110239e-02   8.5128550e-02   8.5123975e-02   8.5138271e-02   8.5124638e-02
8.5130194e-02   8.5102086e-02   8.5115547e-02   8.5128195e-02   8.5120917e-02
```

```
8.5112960e-02   8.5142064e-02   8.5113026e-02   8.5108060e-02   8.5127501e-02
8.5139025e-02   8.5112716e-02   8.5086504e-02   8.5122556e-02   8.5120118e-02
8.5123573e-02   8.5125513e-02   8.5106796e-02   8.5111735e-02   8.5143201e-02
8.5119237e-02   8.5106505e-02   8.5097049e-02   8.5131613e-02   8.5128868e-02;
   8.4882194e-02   8.4860866e-02   8.4881147e-02   8.4874972e-02   8.4880601e-02
  8.4882886e-02   8.4881577e-02   8.4882209e-02   8.4874850e-02   8.4879617e-02
 8.4885107e-02   8.4860895e-02   8.4871341e-02   8.4906805e-02   8.4872891e-02
8.4911317e-02   8.4861150e-02   8.4850781e-02   8.4888358e-02   8.4869820e-02
8.4876465e-02   8.4909954e-02   8.4879108e-02   8.4849149e-02   8.4880837e-02
8.4875712e-02   8.4873918e-02   8.4865338e-02   8.4861433e-02   8.4876206e-02
8.4867127e-02   8.4881326e-02   8.4869194e-02   8.4875982e-02   8.4862663e-02
8.4865014e-02   8.4861783e-02   8.4872134e-02   8.4860730e-02   8.4873583e-02
8.4853913e-02   8.4877730e-02   8.4883763e-02   8.4875371e-02   8.4860529e-02
8.4874269e-02   8.4883096e-02   8.4877254e-02   8.4882115e-02   8.4856403e-02];
u_max_2 =   [1.8464049e+00   1.8709514e+00   1.8492804e+00   1.8583833e+00
1.8571624e+00   1.8609182e+00   1.8694150e+00   1.8475026e+00   1.8487098e+00
1.8584379e+00   1.8528077e+00   1.8562641e+00   1.8720831e+00   1.8774979e+00
1.8805023e+00   1.8610383e+00   1.8690563e+00   1.8645103e+00   1.8666813e+00
1.8474735e+00   1.8592776e+00   1.8547636e+00   1.8617043e+00   1.8613162e+00
1.8692392e+00   1.8668045e+00   1.8631039e+00   1.8565922e+00   1.8765870e+00
1.8448274e+00   1.8509944e+00   1.8589309e+00   1.8649916e+00   1.8590262e+00
1.8656570e+00   1.8627440e+00   1.8430535e+00   1.8572958e+00   1.8571879e+00
1.8701193e+00   1.8653696e+00   1.8555425e+00   1.8650379e+00   1.8623794e+00
1.8690092e+00   1.8642295e+00   1.8630262e+00   1.8650171e+00   1.8537728e+00
1.8637528e+00;
   2.0495060e+00   2.0663610e+00   2.0625018e+00   2.0547667e+00   2.0630510e+00
  2.0428453e+00   2.0639017e+00   2.0594632e+00   2.0632675e+00   2.0592213e+00
 2.0547285e+00   2.0649094e+00   2.0630500e+00   2.0619745e+00   2.0752606e+00
2.0385325e+00   2.0530966e+00   2.0530541e+00   2.0432133e+00   2.0478576e+00
2.0327998e+00   2.0451340e+00   2.0458437e+00   2.0547734e+00   2.0597274e+00
2.0619341e+00   2.0594637e+00   2.0687492e+00   2.0520044e+00   2.0563333e+00
2.0725082e+00   2.0631928e+00   2.0608919e+00   2.0504697e+00   2.0478108e+00
2.0447464e+00   2.0486330e+00   2.0490190e+00   2.0435439e+00   2.0559841e+00
2.0729886e+00   2.0467094e+00   2.0626974e+00   2.0649663e+00   2.0638388e+00
2.0539002e+00   2.0418442e+00   2.0622841e+00   2.0536828e+00   2.0447662e+00;
   2.2123958e+00   2.2183202e+00   2.2192719e+00   2.2194018e+00   2.2285171e+00
  2.2203034e+00   2.2186031e+00   2.2102220e+00   2.2137739e+00   2.2017498e+00
 2.2168329e+00   2.2100425e+00   2.2153056e+00   2.2179040e+00   2.2078098e+00
2.2206668e+00   2.2147837e+00   2.2136605e+00   2.2171036e+00   2.2207824e+00
2.2033226e+00   2.2209451e+00   2.2166385e+00   2.2214496e+00   2.2189833e+00
2.2194973e+00   2.2158061e+00   2.2197483e+00   2.2220057e+00   2.2225839e+00
2.2181075e+00   2.2245043e+00   2.2238524e+00   2.2172799e+00   2.2072862e+00
2.2146632e+00   2.2049946e+00   2.2176387e+00   2.2171562e+00   2.2212780e+00
2.2190475e+00   2.2182771e+00   2.2113108e+00   2.2112487e+00   2.2088099e+00
2.2090844e+00   2.2267398e+00   2.2173030e+00   2.2189511e+00   2.2091584e+00;
   2.2561182e+00   2.2582595e+00   2.2523398e+00   2.2642908e+00   2.2515074e+00
  2.2538169e+00   2.2546550e+00   2.2494434e+00   2.2509010e+00   2.2544652e+00
 2.2623064e+00   2.2668370e+00   2.2604290e+00   2.2561656e+00   2.2530189e+00
2.2559105e+00   2.2616120e+00   2.2579226e+00   2.2555861e+00   2.2605050e+00
2.2564089e+00   2.2601560e+00   2.2558344e+00   2.2539234e+00   2.2592836e+00
2.2559191e+00   2.2619168e+00   2.2582540e+00   2.2547952e+00   2.2469721e+00
2.2549066e+00   2.2600260e+00   2.2574764e+00   2.2498907e+00   2.2599936e+00
2.2525923e+00   2.2607320e+00   2.2535504e+00   2.2552564e+00   2.2602592e+00
2.2559744e+00   2.2524592e+00   2.2587444e+00   2.2603632e+00   2.2607719e+00
```

```
2.2606375e+00   2.2572434e+00   2.2559007e+00   2.2656302e+00   2.2672149e+00;
   2.1844593e+00   2.1770651e+00   2.1789652e+00   2.1715462e+00   2.1713481e+00
  2.1786656e+00   2.1776069e+00   2.1732099e+00   2.1788055e+00   2.1772821e+00
 2.1752808e+00   2.1628234e+00   2.1738474e+00   2.1681698e+00   2.1694176e+00
2.1832496e+00   2.1745864e+00   2.1756684e+00   2.1756861e+00   2.1746276e+00
2.1709921e+00   2.1761331e+00   2.1731651e+00   2.1814815e+00   2.1753646e+00
2.1788606e+00   2.1684476e+00   2.1744799e+00   2.1769234e+00   2.1762051e+00
2.1729285e+00   2.1822304e+00   2.1719689e+00   2.1692412e+00   2.1777496e+00
2.1820160e+00   2.1713659e+00   2.1626925e+00   2.1768012e+00   2.1756568e+00
2.1768486e+00   2.1780156e+00   2.1697919e+00   2.1721284e+00   2.1834769e+00
2.1751021e+00   2.1714181e+00   2.1636169e+00   2.1788683e+00   2.1758912e+00;
   2.0684406e+00   2.0574217e+00   2.0685530e+00   2.0650183e+00   2.0689882e+00
  2.0692327e+00   2.0680008e+00   2.0681916e+00   2.0644024e+00   2.0677725e+00
 2.0707706e+00   2.0574942e+00   2.0636216e+00   2.0805825e+00   2.0641491e+00
2.0824415e+00   2.0584059e+00   2.0538832e+00   2.0707305e+00   2.0630002e+00
2.0666144e+00   2.0820138e+00   2.0682831e+00   2.0513167e+00   2.0667668e+00
2.0656782e+00   2.0653697e+00   2.0600751e+00   2.0586924e+00   2.0655812e+00
2.0613971e+00   2.0664532e+00   2.0634164e+00   2.0657907e+00   2.0600939e+00
2.0610427e+00   2.0580362e+00   2.0634876e+00   2.0577105e+00   2.0640451e+00
2.0548279e+00   2.0670013e+00   2.0685588e+00   2.0646510e+00   2.0578166e+00
2.0643560e+00   2.0687078e+00   2.0654680e+00   2.0685182e+00   2.0575495e+00];

 delay_1  delay_2   mu1_mean mu1_median   mu1_mode mu1_HDIlow mu1_HDIhigh
Vdet1 mu1_pcgtVdet1 mu1_ROPElow mu1_ROPEhigh mu1_pcInROPE
     -10       -5     1.8614     1.8614     1.8614     1.8599      1.8630
1.8440         100.00       1.8340       1.8540         0.00
     -10        0     1.8614     1.8614     1.8614     1.8598      1.8630
1.8440         100.00       1.8340       1.8540         0.00
     -10        5     1.8614     1.8614     1.8614     1.8598      1.8630
1.8440         100.00       1.8340       1.8540         0.00
     -10       10     1.8614     1.8614     1.8614     1.8598      1.8630
1.8440         100.00       1.8340       1.8540         0.00
     -10       15     1.8614     1.8614     1.8614     1.8598      1.8630
1.8440         100.00       1.8340       1.8540         0.00
      -5        0     2.0542     2.0542     2.0542     2.0524      2.0559
2.0620           0.00       2.0520       2.0720        99.22
      -5        5     2.0542     2.0542     2.0541     2.0524      2.0559
2.0620           0.00       2.0520       2.0720        99.28
      -5       10     2.0542     2.0542     2.0541     2.0525      2.0560
2.0620           0.00       2.0520       2.0720        99.28
      -5       15     2.0542     2.0542     2.0542     2.0524      2.0559
2.0620           0.00       2.0520       2.0720        99.27
       0        5     2.2163     2.2163     2.2163     2.2152      2.2174
2.2540           0.00       2.2440       2.2640         0.00
       0       10     2.2163     2.2163     2.2163     2.2152      2.2174
2.2540           0.00       2.2440       2.2640         0.00
       0       15     2.2163     2.2163     2.2163     2.2152      2.2174
2.2540           0.00       2.2440       2.2640         0.00
       5       10     2.2575     2.2575     2.2575     2.2566      2.2584
2.2920           0.00       2.2820       2.3020         0.00
       5       15     2.2575     2.2575     2.2575     2.2567      2.2584
2.2920           0.00       2.2820       2.3020         0.00
      10       15     2.1753     2.1753     2.1753     2.1742      2.1765
2.1730          99.99       2.1630       2.1830       100.00
```

delay_1	delay_2	mu2_mean	mu2_median	mu2_mode	mu2_HDIlow	mu2_HDIhigh	Vdet2	mu2_pcgtVdet1	mu2_ROPElow	mu2_ROPEhigh	mu2_pcInROPE
-10	-5	2.0542	2.0542	2.0542	2.0524	2.0560	2.0620	0.00	2.0520	2.0720	99.25
-10	0	2.2163	2.2163	2.2163	2.2152	2.2174	2.2540	0.00	2.2440	2.2640	0.00
-10	5	2.2575	2.2575	2.2575	2.2566	2.2583	2.2920	0.00	2.2820	2.3020	0.00
-10	10	2.1754	2.1754	2.1753	2.1742	2.1765	2.1730	100.00	2.1630	2.1830	100.00
-10	15	2.0647	2.0647	2.0648	2.0635	2.0659	2.0460	100.00	2.0360	2.0560	0.00
-5	0	2.2163	2.2163	2.2163	2.2152	2.2174	2.2540	0.00	2.2440	2.2640	0.00
-5	5	2.2575	2.2575	2.2575	2.2566	2.2583	2.2920	0.00	2.2820	2.3020	0.00
-5	10	2.1754	2.1754	2.1754	2.1742	2.1765	2.1730	99.99	2.1630	2.1830	100.00
-5	15	2.0648	2.0648	2.0648	2.0636	2.0660	2.0460	100.00	2.0360	2.0560	0.00
0	5	2.2575	2.2575	2.2575	2.2566	2.2583	2.2920	0.00	2.2820	2.3020	0.00
0	10	2.1754	2.1754	2.1754	2.1742	2.1765	2.1730	100.00	2.1630	2.1830	100.00
0	15	2.0648	2.0648	2.0648	2.0636	2.0659	2.0460	100.00	2.0360	2.0560	0.00
5	10	2.1754	2.1754	2.1754	2.1742	2.1765	2.1730	100.00	2.1630	2.1830	100.00
5	15	2.0647	2.0648	2.0647	2.0635	2.0659	2.0460	100.00	2.0360	2.0560	0.00
10	15	2.0647	2.0648	2.0647	2.0636	2.0659	2.0460	100.00	2.0360	2.0560	0.00

delay_1	delay_2	muDiff_mean	muDiff_median	muDiff_mode	muDiff_HDIlow	muDiff_HDIhigh	muDiff_pcgtZero	muDiff_ROPElow	muDiff_ROPEhigh	pcInROPE
-10	-5	-0.1928	-0.1928	-0.1927	-0.1951	-0.1904	0.00	-0.0100	0.0100	0.00
-10	0	-0.3549	-0.3549	-0.3549	-0.3568	-0.3530	0.00	-0.0100	0.0100	0.00
-10	5	-0.3961	-0.3961	-0.3962	-0.3979	-0.3943	0.00	-0.0100	0.0100	0.00
-10	10	-0.3139	-0.3139	-0.3138	-0.3159	-0.3120	0.00	-0.0100	0.0100	0.00
-10	15	-0.2033	-0.2033	-0.2033	-0.2053	-0.2014	0.00	-0.0100	0.0100	0.00
-5	0	-0.1621	-0.1621	-0.1620	-0.1642	-0.1601	0.00	-0.0100	0.0100	0.00
-5	5	-0.2033	-0.2033	-0.2034	-0.2053	-0.2014	0.00	-0.0100	0.0100	0.00
-5	10	-0.1212	-0.1212	-0.1211	-0.1233	-0.1191	0.00	-0.0100	0.0100	0.00
-5	15	-0.0106	-0.0106	-0.0107	-0.0127	-0.0084	0.00	-0.0100	0.0100	29.86

0	5	-0.0412	-0.0412	-0.0413	-0.0426	-0.0398	0.00	-0.0100	0.0100	0.00
0	10	0.0409	0.0409	0.0410	0.0393	0.0425	100.00	-0.0100	0.0100	0.00
0	15	0.1515	0.1515	0.1515	0.1499	0.1531	100.00	-0.0100	0.0100	0.00
5	10	0.0821	0.0821	0.0822	0.0806	0.0835	100.00	-0.0100	0.0100	0.00
5	15	0.1928	0.1928	0.1927	0.1913	0.1942	100.00	-0.0100	0.0100	0.00
10	15	0.1106	0.1106	0.1106	0.1089	0.1122	100.00	-0.0100	0.0100	0.00

delay_1	delay_2	nu_mean	nu_median	nu_mode	nu_HDIlow	nu_HDIhigh	nuLog10_mean	nuLog10_median	nuLog10_mode	nuLog10_HDIlow	nuLog10_HDIhigh
-10	-5	42.3746	33.7446	18.7153	5.0017	103.9590	1.5243	1.5282	1.5692	0.9385	2.0933
-10	0	37.4973	28.8312	16.2227	4.1565	96.7308	1.4585	1.4599	1.4883	0.8432	2.0582
-10	5	34.2775	25.4421	11.7014	3.6528	91.7013	1.4096	1.4056	1.3957	0.7868	2.0305
-10	10	32.7468	24.0727	13.8505	3.7048	88.1509	1.3910	1.3815	1.3753	0.7792	2.0104
-10	15	21.1165	13.3899	7.2431	2.6036	64.4259	1.1741	1.1268	1.0031	0.5962	1.8695
-5	0	47.1242	38.5918	24.8501	5.9583	111.7420	1.5803	1.5865	1.5844	1.0102	2.1180
-5	5	44.5134	36.0090	20.9907	5.4112	107.3300	1.5502	1.5564	1.5718	0.9766	2.1103
-5	10	42.9215	34.2088	19.1868	5.3578	105.2690	1.5328	1.5341	1.5300	0.9552	2.0913
-5	15	32.5222	23.8141	9.8550	3.7569	87.2781	1.3878	1.3768	1.3261	0.7812	2.0028
0	5	40.0704	31.3293	17.3300	4.1727	100.7480	1.4923	1.4959	1.5014	0.8939	2.0890
0	10	38.3418	29.6050	17.3716	4.3215	97.4430	1.4722	1.4714	1.4790	0.8766	2.0643
0	15	27.1138	18.4372	10.5647	3.0198	78.0701	1.2925	1.2657	1.1503	0.6841	1.9528
5	10	35.0682	26.3236	12.2204	4.1527	92.2047	1.4258	1.4203	1.4131	0.8207	2.0350
5	15	23.1425	15.2933	7.6343	2.9757	68.2847	1.2219	1.1845	1.0370	0.6365	1.8941
10	15	22.0851	14.6502	8.8635	2.9764	64.8139	1.2067	1.1658	1.0381	0.6416	1.8729

delay_1	delay_2	effSz_mean	effSz_median	effSz_mode	effSz_HDIlow	effSz_HDIhigh	effSz_pcgtZero	effSz_ROPElow	effSz_ROPEhigh	effSz_pcInROPE
-10	-5	-23.5000	-23.4399	-23.3238	-26.3073	-20.8077	0.0000	-0.1000	0.1000	0.0000
-10	0	-53.3522	-53.1409	-53.0544	-60.4534	-46.4798	0.0000	-0.1000	0.1000	0.0000
-10	5	-64.0794	-63.7719	-63.4527	-73.5930	-55.1529	0.0000	-0.1000	0.1000	0.0000

```
     -10       10   -46.8784     -46.6552   -46.2119     -53.4567       -40.7305
       0.0000        -0.1000         0.1000         0.0000
     -10       15   -31.1496     -30.8772   -30.4192     -36.5272       -26.4361
       0.0000        -0.1000         0.1000         0.0000
      -5        0   -22.4630     -22.4310   -22.2839     -25.1364       -19.7754
       0.0000        -0.1000         0.1000         0.0000
      -5        5   -29.9210     -29.8823   -29.8645     -33.7903       -26.1371
       0.0000        -0.1000         0.1000         0.0000
      -5       10   -16.6377     -16.6081   -16.4892     -18.6299       -14.6260
       0.0000        -0.1000         0.1000         0.0000
      -5       15    -1.4585      -1.4562    -1.4589      -1.7938        -1.1102
       0.0000        -0.1000         0.1000         0.0000
       0        5    -8.4855      -8.4606    -8.4060      -9.5899        -7.4278
       0.0000        -0.1000         0.1000         0.0000
       0       10     7.4314       7.4065     7.3843       6.4883         8.4017
     100.0000        -0.1000         0.1000         0.0000
       0       15    27.7579      27.5535    27.1327      23.9182        32.0876
     100.0000        -0.1000         0.1000         0.0000
       5       10    16.5869      16.5184    16.3388      14.4365        18.8067
     100.0000        -0.1000         0.1000         0.0000
       5       15    39.4774      39.1332    38.7686      33.4390        46.2005
     100.0000        -0.1000         0.1000         0.0000
      10       15    20.1333      19.9856    19.7291      17.2061        23.3750
     100.0000        -0.1000         0.1000         0.0000

 delay_1  delay_2 sigma1_mean sigma1_median sigma1_mode sigma1_HDIlow
sigma1_HDIhigh sigma2_mean sigma2_median  sigma2_mode sigma2_HDIlow
sigma2_HDIhigh sigmaDiff_mean sigmaDiff_median sigmaDiff_mode sigmaDiff_HDIlow
sigmaDiff_HDIhigh sigmaDiff_pcgtZero
     -10       -5    0.007795      0.007771    0.007729      0.006577
0.009072    0.008625      0.008595     0.008541      0.007328       0.009971
 -0.000830        -0.000825      -0.000844        -0.002548          0.000932
          17.06
     -10        0    0.007741      0.007722    0.007654      0.006484
0.009044    0.005395      0.005379     0.005342      0.004547       0.006276
  0.002346         0.002332       0.002343         0.000923          0.003804
          99.95
     -10        5    0.007699      0.007682    0.007635      0.006419
0.009030    0.004217      0.004207     0.004190      0.003518       0.004934
  0.003482         0.003463       0.003458         0.002142          0.004861
         100.00
     -10       10    0.007686      0.007667    0.007640      0.006411
0.009032    0.005589      0.005575     0.005587      0.004672       0.006532
  0.002097         0.002083       0.002049         0.000667          0.003591
          99.80
     -10       15    0.007449      0.007444    0.007431      0.006020
0.008839    0.005536      0.005531     0.005498      0.004408       0.006641
  0.001913         0.001900       0.001846         0.000443          0.003391
          99.55
      -5        0    0.008656      0.008623    0.008620      0.007373
0.009969    0.005453      0.005432     0.005391      0.004640       0.006314
  0.003203         0.003183       0.003150         0.001735          0.004757
         100.00
      -5        5    0.008640      0.008605    0.008550      0.007382
```

```
0.009991    0.004277      0.004262     0.004220      0.003631       0.004973
  0.004363          0.004336       0.004289         0.002951           0.005812
         100.00
      -5        10    0.008626      0.008593    0.008499      0.007371
0.009990    0.005674      0.005653     0.005590      0.004804       0.006592
  0.002953          0.002938       0.002980         0.001444           0.004520
          99.99
      -5        15    0.008525      0.008497    0.008398      0.007204
0.009908    0.005741      0.005733     0.005713      0.004720       0.006767
  0.002784          0.002768       0.002769         0.001246           0.004356
          99.99
       0         5    0.005413      0.005396    0.005346      0.004573
0.006290    0.004251      0.004238     0.004194      0.003576       0.004952
  0.001163          0.001155       0.001185         0.000123           0.002203
          98.72
       0        10    0.005403      0.005389    0.005363      0.004573
0.006284    0.005636      0.005620     0.005616      0.004743       0.006572
 -0.000233         -0.000232      -0.000277        -0.001408           0.000937
          34.65
       0        15    0.005300      0.005292    0.005290      0.004408
0.006226    0.005656      0.005652     0.005584      0.004583       0.006730
 -0.000356         -0.000353      -0.000341        -0.001599           0.000844
          27.96
       5        10    0.004225      0.004215    0.004211      0.003543
0.004931    0.005611      0.005597     0.005580      0.004701       0.006549
 -0.001385         -0.001377      -0.001383        -0.002449          -0.000322
           0.45
       5        15    0.004118      0.004112    0.004118      0.003379
0.004874    0.005589      0.005587     0.005610      0.004498       0.006693
 -0.001471         -0.001463      -0.001493        -0.002629          -0.000369
           0.46
      10        15    0.005461      0.005450    0.005426      0.004502
0.006450    0.005574      0.005569     0.005600      0.004478       0.006663
 -0.000114         -0.000112      -0.000075        -0.001380           0.001106
          42.84


****************************************************
Results for noise_window for additive noise in activation used in simulations:
[-10 ms  10 ms]; 1 random sample per segment.
****************************************************
Each row shows 50 simulation results for additive random noise; from top to
bottom the reference deterministic delay was set to -10 -5, 0, 5, 10, 15 ms
t_l_max_1 =     [5.1783920e-02   5.1759920e-02   5.2167920e-02   5.1199920e-02
5.1631920e-02   5.1655920e-02   5.0935920e-02   5.1623920e-02   5.1903920e-02
5.2839920e-02   5.2207920e-02   5.1919920e-02   5.1535920e-02   5.1359920e-02
5.1319920e-02   5.2327920e-02   5.0295920e-02   5.2391920e-02   5.2207920e-02
5.1783920e-02   5.2383920e-02   5.1367920e-02   5.2047920e-02   5.1079920e-02
5.2215920e-02   5.1687920e-02   5.3039920e-02   5.1135920e-02   5.1511920e-02
5.1127920e-02   5.1847920e-02   5.2711920e-02   5.1687920e-02   5.1935920e-02
5.1647920e-02   5.1543920e-02   5.1239920e-02   5.1479920e-02   5.1447920e-02
5.2047920e-02   5.1399920e-02   5.1735920e-02   5.1199920e-02   5.2167920e-02
5.1703920e-02   5.2327920e-02   5.1847920e-02   5.2343920e-02   5.1695920e-02
5.1663920e-02;
```

```
   5.3535920e-02   5.3151920e-02   5.4071920e-02   5.2999920e-02   5.2943920e-02
  5.2959920e-02   5.3415920e-02   5.3111920e-02   5.3359920e-02   5.3967920e-02
 5.3423920e-02   5.2575920e-02   5.3991920e-02   5.2823920e-02   5.2903920e-02
5.3391920e-02   5.3351920e-02   5.3447920e-02   5.4391920e-02   5.3839920e-02
5.2591920e-02   5.3143920e-02   5.2983920e-02   5.4095920e-02   5.4247920e-02
5.3359920e-02   5.2735920e-02   5.3527920e-02   5.2903920e-02   5.4183920e-02
5.3199920e-02   5.3007920e-02   5.3759920e-02   5.3015920e-02   5.3207920e-02
5.3767920e-02   5.3823920e-02   5.3575920e-02   5.4071920e-02   5.3239920e-02
5.2791920e-02   5.3511920e-02   5.3455920e-02   5.2999920e-02   5.3423920e-02
5.3687920e-02   5.3327920e-02   5.3175920e-02   5.2943920e-02   5.2959920e-02;
   5.5383920e-02   5.5447920e-02   5.5135920e-02   5.5391920e-02   5.5903920e-02
  5.5231920e-02   5.5631920e-02   5.5207920e-02   5.5911920e-02   5.4975920e-02
 5.4695920e-02   5.5335920e-02   5.5919920e-02   5.5495920e-02   5.6015920e-02
5.5759920e-02   5.5335920e-02   5.6167920e-02   5.4871920e-02   5.5655920e-02
5.5511920e-02   5.5031920e-02   5.6343920e-02   5.5559920e-02   5.5191920e-02
5.5183920e-02   5.5607920e-02   5.5551920e-02   5.5127920e-02   5.5591920e-02
5.4255920e-02   5.5607920e-02   5.5399920e-02   5.4407920e-02   5.5551920e-02
5.4655920e-02   5.5487920e-02   5.5447920e-02   5.5559920e-02   5.5143920e-02
5.4911920e-02   5.4999920e-02   5.5375920e-02   5.6007920e-02   5.5927920e-02
5.5927920e-02   5.5183920e-02   5.5095920e-02   5.5631920e-02   5.6175920e-02;
   5.7647920e-02   5.7567920e-02   5.7607920e-02   5.7383920e-02   5.8223920e-02
  5.7863920e-02   5.7367920e-02   5.8223920e-02   5.7479920e-02   5.7791920e-02
 5.7311920e-02   5.7847920e-02   5.7503920e-02   5.8215920e-02   5.8247920e-02
5.7887920e-02   5.8383920e-02   5.8583920e-02   5.7399920e-02   5.7927920e-02
5.8015920e-02   5.8127920e-02   5.7823920e-02   5.8487920e-02   5.7831920e-02
5.7663920e-02   5.8007920e-02   5.8567920e-02   5.8087920e-02   5.7831920e-02
5.7911920e-02   5.8431920e-02   5.7975920e-02   5.8087920e-02   5.7863920e-02
5.8215920e-02   5.8807920e-02   5.7615920e-02   5.7759920e-02   5.8415920e-02
5.8039920e-02   5.6951920e-02   5.7927920e-02   5.7903920e-02   5.7935920e-02
5.8159920e-02   5.7879920e-02   5.8423920e-02   5.8487920e-02   5.7607920e-02;
   6.0407920e-02   6.0559920e-02   6.0759920e-02   6.0815920e-02   6.0799920e-02
  6.0743920e-02   5.9871920e-02   6.1231920e-02   5.9999920e-02   6.0719920e-02
 6.0263920e-02   6.1223920e-02   6.0719920e-02   6.0375920e-02   6.1143920e-02
6.1087920e-02   6.0847920e-02   6.0135920e-02   6.0503920e-02   6.1159920e-02
6.1095920e-02   6.1519920e-02   6.0671920e-02   5.9551920e-02   6.1215920e-02
6.1007920e-02   6.1159920e-02   6.0423920e-02   6.1287920e-02   6.0343920e-02
6.0503920e-02   6.1199920e-02   6.0711920e-02   6.1207920e-02   6.1255920e-02
6.0199920e-02   6.1031920e-02   6.0847920e-02   6.0919920e-02   6.0359920e-02
6.0167920e-02   6.0551920e-02   6.0471920e-02   6.1055920e-02   6.1215920e-02
6.1071920e-02   6.1111920e-02   6.0495920e-02   6.1287920e-02   6.0591920e-02;
   6.3847920e-02   6.3463920e-02   6.4391920e-02   6.4039920e-02   6.3687920e-02
  6.3839920e-02   6.3935920e-02   6.3487920e-02   6.3903920e-02   6.3959920e-02
 6.3487920e-02   6.3839920e-02   6.3647920e-02   6.3615920e-02   6.3831920e-02
6.3847920e-02   6.2807920e-02   6.3487920e-02   6.3543920e-02   6.3751920e-02
6.3079920e-02   6.3903920e-02   6.3391920e-02   6.3847920e-02   6.3791920e-02
6.4103920e-02   6.4295920e-02   6.3951920e-02   6.3071920e-02   6.3807920e-02
6.3623920e-02   6.4191920e-02   6.3815920e-02   6.3559920e-02   6.3575920e-02
6.3471920e-02   6.3471920e-02   6.3879920e-02   6.3559920e-02   6.3223920e-02
6.3671920e-02   6.3911920e-02   6.3143920e-02   6.4287920e-02   6.3767920e-02
6.3335920e-02   6.3791920e-02   6.3695920e-02   6.3351920e-02   6.3415920e-02];
t_u_max_1 =      [3.9631920e-02   3.9735920e-02   4.0207920e-02   3.8903920e-02
3.9063920e-02   3.9087920e-02   3.8487920e-02   3.9407920e-02   4.0007920e-02
4.1007920e-02   4.0351920e-02   3.9799920e-02   3.9447920e-02   3.9311920e-02
3.9023920e-02   4.0567920e-02   3.8127920e-02   3.9295920e-02   4.0071920e-02
```

3.9103920e-02 4.0183920e-02 3.8823920e-02 3.9623920e-02 3.8647920e-02
3.9927920e-02 3.9487920e-02 4.1239920e-02 3.8663920e-02 3.9623920e-02
3.8463920e-02 3.9711920e-02 4.0599920e-02 3.9463920e-02 3.9159920e-02
3.9719920e-02 3.8911920e-02 3.9231920e-02 3.9031920e-02 3.9119920e-02
3.9599920e-02 3.9511920e-02 3.9183920e-02 3.8751920e-02 4.0503920e-02
3.9351920e-02 3.9991920e-02 3.9615920e-02 4.0423920e-02 3.9055920e-02
3.9231920e-02;
4.1503920e-02 4.1183920e-02 4.2359920e-02 4.0823920e-02 4.1367920e-02
4.0407920e-02 4.1487920e-02 4.0503920e-02 4.1543920e-02 4.1975920e-02
4.1303920e-02 4.0487920e-02 4.2239920e-02 4.0399920e-02 4.1143920e-02
4.0943920e-02 4.1399920e-02 4.1799920e-02 4.1983920e-02 4.1903920e-02
3.9871920e-02 4.1311920e-02 4.1007920e-02 4.1847920e-02 4.2447920e-02
4.1407920e-02 4.1047920e-02 4.1231920e-02 4.0815920e-02 4.2295920e-02
4.1263920e-02 4.1351920e-02 4.1663920e-02 4.1183920e-02 4.1391920e-02
4.1903920e-02 4.1607920e-02 4.1823920e-02 4.2151920e-02 4.0847920e-02
4.0727920e-02 4.1519920e-02 4.1271920e-02 4.1095920e-02 4.1591920e-02
4.1775920e-02 4.0839920e-02 4.1407920e-02 4.1151920e-02 4.0999920e-02;
4.3919920e-02 4.4031920e-02 4.3255920e-02 4.3919920e-02 4.4303920e-02
4.3423920e-02 4.4087920e-02 4.3295920e-02 4.4503920e-02 4.3423920e-02
4.3279920e-02 4.3743920e-02 4.4335920e-02 4.3047920e-02 4.4159920e-02
4.4335920e-02 4.3511920e-02 4.4759920e-02 4.3111920e-02 4.4079920e-02
4.3815920e-02 4.3303920e-02 4.4703920e-02 4.4071920e-02 4.3647920e-02
4.3719920e-02 4.3943920e-02 4.3599920e-02 4.3399920e-02 4.4079920e-02
4.2455920e-02 4.4047920e-02 4.3479920e-02 4.2599920e-02 4.3895920e-02
4.2951920e-02 4.4087920e-02 4.3775920e-02 4.3567920e-02 4.3479920e-02
4.3359920e-02 4.3223920e-02 4.3903920e-02 4.4191920e-02 4.4479920e-02
4.4367920e-02 4.3495920e-02 4.3303920e-02 4.3871920e-02 4.4615920e-02;
4.6375920e-02 4.6239920e-02 4.6271920e-02 4.5783920e-02 4.6703920e-02
4.6615920e-02 4.5799920e-02 4.6783920e-02 4.6111920e-02 4.6423920e-02
4.5807920e-02 4.6319920e-02 4.6015920e-02 4.6847920e-02 4.6879920e-02
4.6455920e-02 4.7143920e-02 4.7215920e-02 4.5903920e-02 4.6391920e-02
4.6767920e-02 4.6727920e-02 4.6279920e-02 4.7231920e-02 4.6495920e-02
4.6279920e-02 4.6535920e-02 4.7103920e-02 4.6767920e-02 4.6263920e-02
4.6503920e-02 4.7087920e-02 4.6511920e-02 4.6615920e-02 4.6487920e-02
4.6743920e-02 4.7543920e-02 4.6199920e-02 4.6295920e-02 4.7023920e-02
4.6519920e-02 4.5583920e-02 4.6631920e-02 4.6495920e-02 4.6623920e-02
4.6847920e-02 4.6439920e-02 4.7087920e-02 4.7119920e-02 4.6191920e-02;
4.8991920e-02 4.9247920e-02 4.9479920e-02 4.9487920e-02 4.9447920e-02
4.9391920e-02 4.8535920e-02 4.9847920e-02 4.8671920e-02 4.9471920e-02
4.8863920e-02 4.9815920e-02 4.9423920e-02 4.9007920e-02 4.9863920e-02
4.9679920e-02 4.9551920e-02 4.8879920e-02 4.9111920e-02 4.9839920e-02
4.9711920e-02 5.0111920e-02 4.9479920e-02 4.8119920e-02 4.9927920e-02
4.9623920e-02 4.9647920e-02 4.9031920e-02 4.9919920e-02 4.8935920e-02
4.9303920e-02 4.9879920e-02 4.9479920e-02 4.9951920e-02 4.9991920e-02
4.8815920e-02 4.9727920e-02 4.9439920e-02 4.9583920e-02 4.9063920e-02
4.8783920e-02 4.9247920e-02 4.9063920e-02 4.9607920e-02 4.9919920e-02
4.9759920e-02 4.9831920e-02 4.9319920e-02 5.0031920e-02 4.9359920e-02;
5.2447920e-02 5.1967920e-02 5.2959920e-02 5.2735920e-02 5.2303920e-02
5.2423920e-02 5.2695920e-02 5.2135920e-02 5.2471920e-02 5.2327920e-02
5.2111920e-02 5.2519920e-02 5.2135920e-02 5.2207920e-02 5.2503920e-02
5.2407920e-02 5.1439920e-02 5.2119920e-02 5.2151920e-02 5.2487920e-02
5.1695920e-02 5.2495920e-02 5.2063920e-02 5.2471920e-02 5.2431920e-02
5.2711920e-02 5.2983920e-02 5.2495920e-02 5.1663920e-02 5.2431920e-02
5.2271920e-02 5.2887920e-02 5.2423920e-02 5.2151920e-02 5.2223920e-02

```
5.2111920e-02   5.1999920e-02   5.2615920e-02   5.2151920e-02   5.1807920e-02
5.2327920e-02   5.2599920e-02   5.1663920e-02   5.2951920e-02   5.2391920e-02
5.2079920e-02   5.2343920e-02   5.2183920e-02   5.1927920e-02   5.1983920e-02];
l_max_1 =       [8.5058685e-02   8.5065158e-02   8.5070846e-02   8.4998556e-02
8.4957906e-02   8.5056523e-02   8.5148472e-02   8.5022979e-02   8.5012293e-02
8.4997612e-02   8.5131709e-02   8.5077520e-02   8.5081896e-02   8.5179215e-02
8.5026729e-02   8.5033851e-02   8.5111350e-02   8.4826331e-02   8.5026444e-02
8.5023830e-02   8.5018811e-02   8.5088335e-02   8.5092263e-02   8.5097417e-02
8.5038678e-02   8.5078713e-02   8.4946371e-02   8.5004175e-02   8.5063038e-02
8.4997315e-02   8.5122225e-02   8.4989768e-02   8.5054082e-02   8.5074571e-02
8.5090840e-02   8.5009587e-02   8.5079206e-02   8.5138883e-02   8.5125064e-02
8.4962291e-02   8.5118083e-02   8.5031880e-02   8.5062374e-02   8.5062367e-02
8.4963663e-02   8.5092804e-02   8.5069351e-02   8.4996160e-02   8.5018077e-02
8.5033697e-02;
   8.5159949e-02   8.5119357e-02   8.5252208e-02   8.5144547e-02   8.5262825e-02
  8.5072398e-02   8.5113373e-02   8.5089430e-02   8.5190320e-02   8.5129026e-02
 8.5123222e-02   8.5109896e-02   8.5134350e-02   8.5093151e-02   8.5257870e-02
8.5067383e-02   8.5149169e-02   8.5274295e-02   8.5092173e-02   8.5176959e-02
8.5130807e-02   8.5139676e-02   8.5126409e-02   8.5117396e-02   8.5131969e-02
8.5160913e-02   8.5215764e-02   8.5102273e-02   8.5143557e-02   8.5198187e-02
8.5231371e-02   8.5194388e-02   8.5198649e-02   8.5233673e-02   8.5190177e-02
8.5154646e-02   8.5122808e-02   8.5258235e-02   8.5114478e-02   8.5164342e-02
8.5172600e-02   8.5169647e-02   8.5096535e-02   8.5248411e-02   8.5150454e-02
8.5149322e-02   8.5147894e-02   8.5241790e-02   8.5214276e-02   8.5211136e-02;
   8.5264477e-02   8.5267356e-02   8.5251955e-02   8.5215828e-02   8.5169915e-02
  8.5234453e-02   8.5168311e-02   8.5195128e-02   8.5213955e-02   8.5196266e-02
 8.5254565e-02   8.5249439e-02   8.5280052e-02   8.5138258e-02   8.5193515e-02
8.5172469e-02   8.5166088e-02   8.5197729e-02   8.5166519e-02   8.5236600e-02
8.5221674e-02   8.5146322e-02   8.5173623e-02   8.5138542e-02   8.5196315e-02
8.5134921e-02   8.5235487e-02   8.5250616e-02   8.5248900e-02   8.5260818e-02
8.5274865e-02   8.5181334e-02   8.5250583e-02   8.5233132e-02   8.5235962e-02
8.5230028e-02   8.5211826e-02   8.5230995e-02   8.5181608e-02   8.5202548e-02
8.5315735e-02   8.5138221e-02   8.5275445e-02   8.5088850e-02   8.5237621e-02
8.5187102e-02   8.5187687e-02   8.5174488e-02   8.5144000e-02   8.5228623e-02;
   8.5254597e-02   8.5181406e-02   8.5070067e-02   8.5095805e-02   8.5157878e-02
  8.5018819e-02   8.5175450e-02   8.5079102e-02   8.5174876e-02   8.5138564e-02
 8.5081026e-02   8.5096756e-02   8.5180865e-02   8.5118276e-02   8.5121886e-02
8.5099244e-02   8.5189631e-02   8.5119869e-02   8.5148042e-02   8.5144879e-02
8.5219697e-02   8.5122526e-02   8.5064345e-02   8.5074046e-02   8.5186693e-02
8.5223396e-02   8.5133188e-02   8.5206493e-02   8.5054407e-02   8.5166722e-02
8.5100718e-02   8.5202905e-02   8.5066684e-02   8.5153079e-02   8.5060396e-02
8.5165059e-02   8.5101220e-02   8.5168943e-02   8.5119308e-02   8.5048953e-02
8.5198459e-02   8.5082104e-02   8.5222273e-02   8.5203574e-02   8.5128741e-02
8.5098705e-02   8.5166759e-02   8.5209825e-02   8.5110372e-02   8.5024529e-02;
   8.5026730e-02   8.5157213e-02   8.4952824e-02   8.5093227e-02   8.4909674e-02
  8.5058321e-02   8.4924733e-02   8.4898902e-02   8.5037446e-02   8.4949640e-02
 8.5156541e-02   8.4981865e-02   8.5120362e-02   8.5006651e-02   8.4901578e-02
8.4885708e-02   8.4980107e-02   8.4941207e-02   8.4946775e-02   8.5052739e-02
8.4969907e-02   8.4887262e-02   8.5086173e-02   8.5069588e-02   8.5003787e-02
8.5146144e-02   8.4903115e-02   8.4713654e-02   8.4914564e-02   8.4984310e-02
8.5018349e-02   8.5050474e-02   8.5030677e-02   8.4957245e-02   8.4940452e-02
8.5081369e-02   8.4977824e-02   8.4946401e-02   8.4987422e-02   8.5026102e-02
8.4990600e-02   8.4959989e-02   8.4995637e-02   8.4926973e-02   8.4939419e-02
8.4883268e-02   8.5051692e-02   8.5015856e-02   8.4929383e-02   8.4989467e-02;
```

```
   8.4805718e-02   8.4722127e-02   8.4789866e-02   8.4840288e-02   8.4834157e-02
  8.4788058e-02   8.4827021e-02   8.4826191e-02   8.4739011e-02   8.4716279e-02
 8.4843449e-02   8.4831654e-02   8.4791032e-02   8.4693882e-02   8.4882443e-02
8.4835838e-02   8.4957440e-02   8.4744664e-02   8.4756553e-02   8.4888531e-02
8.4833413e-02   8.4785074e-02   8.4910540e-02   8.4761986e-02   8.4733551e-02
8.4721346e-02   8.4808523e-02   8.4853868e-02   8.4760493e-02   8.4734892e-02
8.4851280e-02   8.4846723e-02   8.4656075e-02   8.4832697e-02   8.4738558e-02
8.4793245e-02   8.4705660e-02   8.4892963e-02   8.4755757e-02   8.4715607e-02
8.4783670e-02   8.4732057e-02   8.4806221e-02   8.4815919e-02   8.4833721e-02
8.4849008e-02   8.4711719e-02   8.4807069e-02   8.4795738e-02   8.4767352e-02];
u_max_1 =       [1.8068642e+00   1.8408161e+00   1.8127225e+00   1.7690031e+00
1.7592846e+00   1.8519904e+00   1.9383028e+00   1.7841598e+00   1.7637613e+00
1.7604329e+00   1.8554527e+00   1.8124865e+00   1.8395574e+00   1.8930461e+00
1.8015160e+00   1.7596780e+00   1.8620243e+00   1.6742515e+00   1.7928336e+00
1.8351413e+00   1.8039448e+00   1.8482146e+00   1.8646639e+00   1.8703955e+00
1.7926885e+00   1.8364973e+00   1.7158301e+00   1.8144166e+00   1.8151314e+00
1.8163720e+00   1.8675407e+00   1.7621585e+00   1.8025652e+00   1.8404174e+00
1.8062878e+00   1.8090874e+00   1.8388504e+00   1.9009197e+00   1.8976169e+00
1.7582365e+00   1.8394382e+00   1.8353557e+00   1.8237467e+00   1.8008086e+00
1.7937042e+00   1.8354640e+00   1.8434589e+00   1.7366401e+00   1.7925095e+00
1.8063237e+00;
   1.9357852e+00   1.9395176e+00   1.9714439e+00   1.9688095e+00   2.0419798e+00
  1.8825523e+00   1.8704256e+00   1.8967332e+00   1.9671205e+00   1.9581727e+00
 1.9360033e+00   1.9109134e+00   1.9110141e+00   1.8950104e+00   1.9964836e+00
1.8834171e+00   1.9392978e+00   2.0199500e+00   1.8604070e+00   1.9576348e+00
1.9373014e+00   1.9264579e+00   1.9419104e+00   1.8856193e+00   1.8986689e+00
1.9500330e+00   1.9629957e+00   1.8862446e+00   1.9464889e+00   1.9313894e+00
1.9925077e+00   1.9761684e+00   1.9465817e+00   2.0086493e+00   1.9464328e+00
1.9422458e+00   1.9151851e+00   1.9849936e+00   1.8983750e+00   1.9748324e+00
1.9446188e+00   1.9657406e+00   1.8905374e+00   1.9827725e+00   1.9234543e+00
1.9386214e+00   1.9414541e+00   1.9805229e+00   1.9890338e+00   1.9848859e+00;
   2.0681492e+00   2.0682781e+00   2.0450731e+00   2.0842039e+00   2.0071058e+00
  2.0152541e+00   2.0306722e+00   2.0122213e+00   2.0584437e+00   2.0693933e+00
 2.1204828e+00   2.0590444e+00   2.0647111e+00   1.9846159e+00   1.9882700e+00
2.0372873e+00   2.0012028e+00   2.0599792e+00   2.0149541e+00   2.0267184e+00
2.0609742e+00   1.9766081e+00   2.0035027e+00   2.0229530e+00   2.0289754e+00
2.0032890e+00   2.0745372e+00   2.0467066e+00   2.0672793e+00   2.0654606e+00
2.0696529e+00   1.9988070e+00   2.0433969e+00   2.0349009e+00   2.0477680e+00
2.0629501e+00   2.0902572e+00   2.0417047e+00   2.0011972e+00   2.0411548e+00
2.0888735e+00   1.9917745e+00   2.0737902e+00   1.9699683e+00   2.0566237e+00
2.0151622e+00   2.0170513e+00   2.0193673e+00   2.0094506e+00   2.0456785e+00;
   2.1230213e+00   2.1170768e+00   2.0681801e+00   2.0516041e+00   2.0610444e+00
  2.0483920e+00   2.0809205e+00   2.0804791e+00   2.0923490e+00   2.0648880e+00
 2.0487546e+00   2.0594046e+00   2.1112501e+00   2.0539518e+00   2.0632740e+00
2.0326060e+00   2.1067665e+00   2.0552774e+00   2.0651197e+00   2.0572471e+00
2.1285352e+00   2.0616820e+00   2.0721688e+00   2.0680400e+00   2.1062153e+00
2.0809659e+00   2.0423218e+00   2.0989048e+00   2.0653680e+00   2.0542142e+00
2.0647952e+00   2.0825190e+00   2.0334453e+00   2.0652872e+00   2.0524257e+00
2.0745527e+00   2.0573125e+00   2.0796400e+00   2.0593562e+00   2.0509136e+00
2.0973621e+00   2.0529758e+00   2.0957785e+00   2.0776549e+00   2.0788383e+00
2.0790200e+00   2.0801445e+00   2.0587838e+00   2.0468945e+00   2.0361344e+00;
   2.0356633e+00   2.1092330e+00   2.0455020e+00   2.0826917e+00   2.0367390e+00
  2.0498132e+00   2.0440528e+00   2.0334431e+00   2.0734082e+00   2.0517091e+00
 2.1137863e+00   2.0363531e+00   2.1099282e+00   2.0665965e+00   2.0357288e+00
```

```
2.0071693e+00   2.0486817e+00   2.0536914e+00   2.0440356e+00   2.0789749e+00
2.0416441e+00   2.0169145e+00   2.1111170e+00   2.0620059e+00   2.0210833e+00
2.1158922e+00   1.9920023e+00   1.9457895e+00   2.0270674e+00   2.0379866e+00
2.1066021e+00   2.0926376e+00   2.1069437e+00   2.0468988e+00   2.0371942e+00
2.0822073e+00   2.0632949e+00   2.0488764e+00   2.0623528e+00   2.0732978e+00
2.0630922e+00   2.0636815e+00   2.0519127e+00   2.0242620e+00   2.0530214e+00
2.0303522e+00   2.0689040e+00   2.0783277e+00   2.0552395e+00   2.0660748e+00;
   1.9903688e+00   1.9628380e+00   1.9863928e+00   2.0127346e+00   1.9992198e+00
  1.9921681e+00   2.0124379e+00   2.0135246e+00   1.9773829e+00   1.9535986e+00
 2.0079413e+00   2.0158975e+00   1.9963700e+00   1.9524159e+00   2.0369288e+00
2.0209749e+00   2.0580722e+00   1.9626112e+00   1.9792634e+00   2.0400129e+00
2.0180382e+00   1.9954811e+00   2.0356843e+00   1.9798058e+00   1.9449312e+00
1.9423877e+00   2.0051748e+00   2.0113219e+00   1.9779619e+00   1.9527822e+00
2.0173228e+00   2.0074365e+00   1.9316293e+00   1.9961634e+00   1.9649909e+00
2.0010698e+00   1.9642663e+00   2.0387431e+00   1.9521316e+00   1.9605457e+00
1.9976385e+00   1.9688336e+00   1.9944026e+00   1.9902245e+00   2.0254023e+00
2.0238488e+00   1.9478489e+00   2.0125552e+00   1.9911678e+00   1.9690455e+00];
t_l_max_2 = [5.1919920e-02   5.1551920e-02   5.2143920e-02   5.2399920e-02
5.1279920e-02   5.2031920e-02   5.1391920e-02   5.1535920e-02   5.1695920e-02
5.2111920e-02   5.0543920e-02   5.2175920e-02   5.2015920e-02   5.1487920e-02
5.1647920e-02   5.1487920e-02   5.2351920e-02   5.1599920e-02   5.2111920e-02
5.2639920e-02   5.1487920e-02   5.1919920e-02   5.0959920e-02   5.1487920e-02
5.1567920e-02   5.1023920e-02   5.1967920e-02   5.2303920e-02   5.2031920e-02
5.1663920e-02   5.1647920e-02   5.1807920e-02   5.1983920e-02   5.1583920e-02
5.1903920e-02   5.1599920e-02   5.2847920e-02   5.2479920e-02   5.2399920e-02
5.0623920e-02   5.2175920e-02   5.0991920e-02   5.1359920e-02   5.1359920e-02
5.1551920e-02   5.1039920e-02   5.1183920e-02   5.2431920e-02   5.1327920e-02
5.1327920e-02;
   5.3055920e-02   5.3663920e-02   5.3455920e-02   5.4255920e-02   5.2879920e-02
  5.3071920e-02   5.2959920e-02   5.4047920e-02   5.3087920e-02   5.2735920e-02
 5.2991920e-02   5.2799920e-02   5.3215920e-02   5.2959920e-02   5.2911920e-02
5.2143920e-02   5.3551920e-02   5.3839920e-02   5.2287920e-02   5.3071920e-02
5.3295920e-02   5.3759920e-02   5.2863920e-02   5.2975920e-02   5.2175920e-02
5.3103920e-02   5.3663920e-02   5.4063920e-02   5.3007920e-02   5.4127920e-02
5.3071920e-02   5.3343920e-02   5.4127920e-02   5.4111920e-02   5.3087920e-02
5.3391920e-02   5.3375920e-02   5.3567920e-02   5.3439920e-02   5.3231920e-02
5.3263920e-02   5.2399920e-02   5.2975920e-02   5.3023920e-02   5.2863920e-02
5.3839920e-02   5.3487920e-02   5.3199920e-02   5.2639920e-02   5.3119920e-02;
   5.4735920e-02   5.5039920e-02   5.4815920e-02   5.4735920e-02   5.4927920e-02
  5.4655920e-02   5.5695920e-02   5.4943920e-02   5.5871920e-02   5.5359920e-02
 5.5663920e-02   5.5887920e-02   5.5951920e-02   5.5199920e-02   5.5359920e-02
5.6111920e-02   5.5839920e-02   5.5503920e-02   5.5567920e-02   5.5759920e-02
5.4879920e-02   5.5535920e-02   5.5791920e-02   5.4959920e-02   5.6927920e-02
5.5743920e-02   5.6191920e-02   5.5279920e-02   5.5999920e-02   5.5343920e-02
5.5407920e-02   5.5743920e-02   5.4895920e-02   5.5455920e-02   5.5119920e-02
5.4991920e-02   5.5327920e-02   5.5999920e-02   5.6799920e-02   5.5279920e-02
5.5599920e-02   5.6239920e-02   5.4991920e-02   5.5055920e-02   5.4223920e-02
5.5983920e-02   5.5647920e-02   5.5359920e-02   5.5743920e-02   5.5999920e-02;
   5.8031920e-02   5.8271920e-02   5.7519920e-02   5.7807920e-02   5.7567920e-02
  5.7391920e-02   5.8031920e-02   5.7679920e-02   5.7071920e-02   5.8495920e-02
 5.8255920e-02   5.8255920e-02   5.8127920e-02   5.7967920e-02   5.7663920e-02
5.7311920e-02   5.7503920e-02   5.7743920e-02   5.6911920e-02   5.7647920e-02
5.7743920e-02   5.7759920e-02   5.7503920e-02   5.7807920e-02   5.8703920e-02
5.7359920e-02   5.7359920e-02   5.7439920e-02   5.7199920e-02   5.8079920e-02
```

```
5.7471920e-02   5.7743920e-02   5.8239920e-02   5.8879920e-02   5.7503920e-02
5.8559920e-02   5.8623920e-02   5.8127920e-02   5.7615920e-02   5.8703920e-02
5.7887920e-02   5.7551920e-02   5.8623920e-02   5.7887920e-02   5.7615920e-02
5.7855920e-02   5.8175920e-02   5.7103920e-02   5.7535920e-02   5.8031920e-02;
   6.1071920e-02   6.0975920e-02   6.1327920e-02   6.0751920e-02   6.0431920e-02
  6.0335920e-02   6.1151920e-02   6.0271920e-02   6.0127920e-02   6.0767920e-02
 6.0271920e-02   6.1247920e-02   6.0463920e-02   6.0623920e-02   6.1199920e-02
6.0927920e-02   6.0703920e-02   6.1039920e-02   6.0591920e-02   6.1375920e-02
6.0255920e-02   6.0255920e-02   6.0495920e-02   6.0591920e-02   6.1279920e-02
6.1135920e-02   6.0959920e-02   6.0655920e-02   6.0031920e-02   6.0623920e-02
6.1055920e-02   6.1263920e-02   6.0335920e-02   6.0607920e-02   6.0191920e-02
6.0687920e-02   6.1167920e-02   6.1791920e-02   6.1503920e-02   6.0607920e-02
6.0255920e-02   6.0399920e-02   5.9983920e-02   6.1151920e-02   6.0415920e-02
6.1119920e-02   6.1311920e-02   6.0879920e-02   6.0431920e-02   6.0815920e-02;
   6.3519920e-02   6.3887920e-02   6.4255920e-02   6.3647920e-02   6.3391920e-02
  6.3759920e-02   6.3359920e-02   6.3711920e-02   6.4159920e-02   6.3919920e-02
 6.2735920e-02   6.3791920e-02   6.4399920e-02   6.3695920e-02   6.3967920e-02
6.3375920e-02   6.3983920e-02   6.3775920e-02   6.3823920e-02   6.3999920e-02
6.4527920e-02   6.3759920e-02   6.2879920e-02   6.4143920e-02   6.3423920e-02
6.3711920e-02   6.3535920e-02   6.3599920e-02   6.3631920e-02   6.3711920e-02
6.3551920e-02   6.3503920e-02   6.3647920e-02   6.3183920e-02   6.4271920e-02
6.3839920e-02   6.4143920e-02   6.3711920e-02   6.3567920e-02   6.3455920e-02
6.2911920e-02   6.4671920e-02   6.4815920e-02   6.3583920e-02   6.4031920e-02
6.3391920e-02   6.3535920e-02   6.3199920e-02   6.3119920e-02   6.4383920e-02];
t_u_max_2 = [4.0159920e-02   3.9375920e-02   3.9519920e-02   4.0367920e-02
3.9503920e-02   3.9215920e-02   3.8927920e-02   3.9087920e-02   3.9119920e-02
3.9967920e-02   3.8527920e-02   4.0015920e-02   3.9967920e-02   3.9503920e-02
3.9327920e-02   3.9695920e-02   3.9311920e-02   3.9727920e-02   3.9775920e-02
3.9919920e-02   3.9167920e-02   3.9391920e-02   3.8783920e-02   3.8927920e-02
3.8975920e-02   3.8751920e-02   4.0063920e-02   3.9647920e-02   4.0047920e-02
3.9359920e-02   3.9887920e-02   3.9167920e-02   3.9871920e-02   3.8863920e-02
4.0191920e-02   3.9631920e-02   4.0623920e-02   4.0287920e-02   3.9695920e-02
3.8479920e-02   3.9583920e-02   3.8671920e-02   3.9279920e-02   3.9055920e-02
3.9215920e-02   3.9071920e-02   3.9135920e-02   4.0319920e-02   3.9183920e-02
3.9039920e-02;
   4.0831920e-02   4.0639920e-02   4.1519920e-02   4.2143920e-02   4.0911920e-02
  4.0895920e-02   4.1007920e-02   4.2095920e-02   4.1007920e-02   4.0751920e-02
 4.1023920e-02   4.1151920e-02   4.1487920e-02   4.0911920e-02   4.0991920e-02
4.0047920e-02   4.1871920e-02   4.1423920e-02   3.9759920e-02   4.1007920e-02
4.0559920e-02   4.1839920e-02   4.0591920e-02   4.1119920e-02   4.0495920e-02
4.0719920e-02   4.1711920e-02   4.2031920e-02   4.1423920e-02   4.1679920e-02
4.1055920e-02   4.1375920e-02   4.1871920e-02   4.2239920e-02   4.0879920e-02
4.1567920e-02   4.1295920e-02   4.1535920e-02   4.1743920e-02   4.1295920e-02
4.0943920e-02   4.0799920e-02   4.0863920e-02   4.1103920e-02   4.1295920e-02
4.1743920e-02   4.1455920e-02   4.0991920e-02   4.0831920e-02   4.1151920e-02;
   4.3135920e-02   4.3439920e-02   4.3071920e-02   4.2975920e-02   4.3311920e-02
  4.3247920e-02   4.4111920e-02   4.3263920e-02   4.4335920e-02   4.3599920e-02
 4.4031920e-02   4.4255920e-02   4.4175920e-02   4.3727920e-02   4.3487920e-02
4.4431920e-02   4.3967920e-02   4.3871920e-02   4.3759920e-02   4.4095920e-02
4.3199920e-02   4.3679920e-02   4.4111920e-02   4.3295920e-02   4.5359920e-02
4.4063920e-02   4.4175920e-02   4.3711920e-02   4.4431920e-02   4.3583920e-02
4.3567920e-02   4.4127920e-02   4.3423920e-02   4.3791920e-02   4.3343920e-02
4.3055920e-02   4.3743920e-02   4.4207920e-02   4.5071920e-02   4.3711920e-02
4.3919920e-02   4.4447920e-02   4.3567920e-02   4.3535920e-02   4.2271920e-02
```

```
4.4207920e-02   4.4111920e-02   4.3679920e-02   4.3951920e-02   4.4191920e-02;
   4.6655920e-02   4.6847920e-02   4.5999920e-02   4.6351920e-02   4.6159920e-02
  4.6031920e-02   4.6591920e-02   4.6399920e-02   4.5519920e-02   4.6959920e-02
 4.7007920e-02   4.6911920e-02   4.6655920e-02   4.6623920e-02   4.6383920e-02
4.5871920e-02   4.6239920e-02   4.6207920e-02   4.5615920e-02   4.6191920e-02
4.6079920e-02   4.6111920e-02   4.6047920e-02   4.6527920e-02   4.7279920e-02
4.5935920e-02   4.5743920e-02   4.6047920e-02   4.5759920e-02   4.6639920e-02
4.6015920e-02   4.6383920e-02   4.6895920e-02   4.7471920e-02   4.5871920e-02
4.7023920e-02   4.7151920e-02   4.6799920e-02   4.6303920e-02   4.7407920e-02
4.6495920e-02   4.6255920e-02   4.7295920e-02   4.6447920e-02   4.6159920e-02
4.6399920e-02   4.6815920e-02   4.5759920e-02   4.6047920e-02   4.6495920e-02;
   4.9695920e-02   4.9503920e-02   4.9855920e-02   4.9343920e-02   4.9231920e-02
  4.9007920e-02   4.9807920e-02   4.9007920e-02   4.8623920e-02   4.9439920e-02
 4.9007920e-02   5.0015920e-02   4.9087920e-02   4.9263920e-02   4.9823920e-02
4.9599920e-02   4.9535920e-02   4.9791920e-02   4.9231920e-02   5.0063920e-02
4.8863920e-02   4.8895920e-02   4.9199920e-02   4.9215920e-02   4.9951920e-02
4.9663920e-02   4.9711920e-02   4.9167920e-02   4.8703920e-02   4.9167920e-02
4.9727920e-02   5.0031920e-02   4.9023920e-02   4.9247920e-02   4.8751920e-02
4.9375920e-02   4.9903920e-02   5.0479920e-02   5.0207920e-02   4.9343920e-02
4.8927920e-02   4.9023920e-02   4.8623920e-02   4.9679920e-02   4.8975920e-02
4.9743920e-02   5.0031920e-02   4.9439920e-02   4.9167920e-02   4.9551920e-02;
   5.1919920e-02   5.2447920e-02   5.2863920e-02   5.2175920e-02   5.1919920e-02
  5.2399920e-02   5.2047920e-02   5.2351920e-02   5.2799920e-02   5.2543920e-02
 5.1263920e-02   5.2559920e-02   5.2975920e-02   5.2383920e-02   5.2639920e-02
5.1935920e-02   5.2575920e-02   5.2287920e-02   5.2447920e-02   5.2687920e-02
5.3151920e-02   5.2367920e-02   5.1423920e-02   5.2799920e-02   5.1887920e-02
5.2143920e-02   5.2239920e-02   5.2239920e-02   5.2191920e-02   5.2255920e-02
5.2111920e-02   5.2079920e-02   5.2223920e-02   5.1855920e-02   5.2943920e-02
5.2511920e-02   5.2783920e-02   5.2287920e-02   5.2255920e-02   5.1903920e-02
5.1535920e-02   5.3327920e-02   5.3487920e-02   5.2079920e-02   5.2671920e-02
5.2095920e-02   5.1983920e-02   5.1919920e-02   5.1727920e-02   5.2975920e-02];
l_max_2 =   [8.5151161e-02   8.5091755e-02   8.4960887e-02   8.4985782e-02
8.5082009e-02   8.4985976e-02   8.5108534e-02   8.5044154e-02   8.4992970e-02
8.5072777e-02   8.5098745e-02   8.5031907e-02   8.5046719e-02   8.5126173e-02
8.5087836e-02   8.5089715e-02   8.4957590e-02   8.5158671e-02   8.5012938e-02
8.4984349e-02   8.5123867e-02   8.4970392e-02   8.5105656e-02   8.5080655e-02
8.5064156e-02   8.5089585e-02   8.5056024e-02   8.4973382e-02   8.5038093e-02
8.5078548e-02   8.5020991e-02   8.4946825e-02   8.4984689e-02   8.5023503e-02
8.5089947e-02   8.5165133e-02   8.5030561e-02   8.5068193e-02   8.4988796e-02
8.5204927e-02   8.5059958e-02   8.5038079e-02   8.5038855e-02   8.5120177e-02
8.5062160e-02   8.5147302e-02   8.5052784e-02   8.5088030e-02   8.5103564e-02
8.5055769e-02;
   8.5233758e-02   8.5074164e-02   8.5212002e-02   8.5192494e-02   8.5206600e-02
  8.5157834e-02   8.5180760e-02   8.5196581e-02   8.5120810e-02   8.5188951e-02
 8.5199061e-02   8.5213847e-02   8.5170442e-02   8.5169968e-02   8.5229634e-02
8.5109002e-02   8.5239721e-02   8.5079558e-02   8.5167632e-02   8.5129463e-02
8.5071457e-02   8.5167666e-02   8.5082689e-02   8.5161110e-02   8.5295964e-02
8.5128237e-02   8.5214315e-02   8.5118126e-02   8.5279784e-02   8.5019570e-02
8.5215244e-02   8.5115263e-02   8.5150209e-02   8.5132874e-02   8.5189698e-02
8.5302648e-02   8.5223182e-02   8.5179769e-02   8.5186648e-02   8.5184964e-02
8.5142763e-02   8.5236940e-02   8.5162753e-02   8.5198450e-02   8.5264513e-02
8.5084307e-02   8.5193838e-02   8.5148655e-02   8.5113088e-02   8.5102200e-02;
   8.5247751e-02   8.5184932e-02   8.5232465e-02   8.5217159e-02   8.5192651e-02
  8.5280453e-02   8.5135344e-02   8.5227520e-02   8.5301588e-02   8.5212701e-02
```

```
 8.5152052e-02   8.5184009e-02   8.5181070e-02   8.5281256e-02   8.5167108e-02
8.5206233e-02   8.5149900e-02   8.5186129e-02   8.5267126e-02   8.5219204e-02
8.5325476e-02   8.5142157e-02   8.5160855e-02   8.5201907e-02   8.5149608e-02
8.5204319e-02   8.5141294e-02   8.5140295e-02   8.5208863e-02   8.5231801e-02
8.5139823e-02   8.5173150e-02   8.5196756e-02   8.5159057e-02   8.5235028e-02
8.5166984e-02   8.5122760e-02   8.5163567e-02   8.5107301e-02   8.5288616e-02
8.5226891e-02   8.5227262e-02   8.5285048e-02   8.5229238e-02   8.5162538e-02
8.5192979e-02   8.5188389e-02   8.5188768e-02   8.5171044e-02   8.5156402e-02;
   8.5072898e-02   8.5035271e-02   8.5109017e-02   8.5171400e-02   8.5036453e-02
  8.5140348e-02   8.5155221e-02   8.5232594e-02   8.5182467e-02   8.5072968e-02
 8.5199522e-02   8.5163195e-02   8.5160764e-02   8.5125657e-02   8.5015514e-02
8.5125023e-02   8.5116344e-02   8.5196371e-02   8.5170838e-02   8.5094699e-02
8.5100894e-02   8.5141512e-02   8.5012942e-02   8.5134002e-02   8.5124233e-02
8.5088372e-02   8.5125556e-02   8.5032438e-02   8.5121714e-02   8.5130192e-02
8.5150916e-02   8.5134414e-02   8.5083727e-02   8.5007055e-02   8.5229277e-02
8.5135198e-02   8.5061588e-02   8.5237092e-02   8.5263908e-02   8.5043417e-02
8.5039833e-02   8.5106863e-02   8.5104750e-02   8.5077693e-02   8.5193016e-02
8.5141356e-02   8.5201131e-02   8.5251593e-02   8.5239699e-02   8.5118124e-02;
   8.4943257e-02   8.4879989e-02   8.4926675e-02   8.5035682e-02   8.5055783e-02
  8.4966755e-02   8.4955475e-02   8.4990356e-02   8.5041775e-02   8.4987124e-02
 8.5021545e-02   8.5024834e-02   8.5032150e-02   8.4951231e-02   8.5010926e-02
8.4964182e-02   8.4964494e-02   8.5049194e-02   8.4907161e-02   8.4963120e-02
8.5066353e-02   8.5024796e-02   8.5037052e-02   8.5073975e-02   8.4997707e-02
8.4928876e-02   8.4979770e-02   8.4938916e-02   8.5052922e-02   8.4959205e-02
8.4866513e-02   8.5030779e-02   8.5034183e-02   8.4996130e-02   8.5052198e-02
8.4995397e-02   8.5002311e-02   8.4945049e-02   8.4975877e-02   8.4998631e-02
8.4953329e-02   8.4989956e-02   8.5057006e-02   8.4967447e-02   8.4989855e-02
8.4887947e-02   8.4995823e-02   8.4933595e-02   8.4971943e-02   8.5012144e-02;
   8.4708474e-02   8.4762744e-02   8.4733465e-02   8.4754527e-02   8.4901580e-02
  8.4711243e-02   8.4781352e-02   8.4734773e-02   8.4821487e-02   8.4707859e-02
 8.4778658e-02   8.4935930e-02   8.4744345e-02   8.4872869e-02   8.4776971e-02
8.4764587e-02   8.4824116e-02   8.4667317e-02   8.4713844e-02   8.4883699e-02
8.4792418e-02   8.4794941e-02   8.4799958e-02   8.4904084e-02   8.4695867e-02
8.4843436e-02   8.4839125e-02   8.4748407e-02   8.4701161e-02   8.4718652e-02
8.4735557e-02   8.4849715e-02   8.4826320e-02   8.4783677e-02   8.4791151e-02
8.4815017e-02   8.4901476e-02   8.4698919e-02   8.4724801e-02   8.4812195e-02
8.4757287e-02   8.4724924e-02   8.4908910e-02   8.4739013e-02   8.4847894e-02
8.4873821e-02   8.4775980e-02   8.4901829e-02   8.4699460e-02   8.4695588e-02];
u_max_2 =   [1.8681225e+00   1.8749871e+00   1.7709866e+00   1.7410924e+00
1.8259409e+00   1.7741019e+00   1.8781516e+00   1.8115721e+00   1.7962147e+00
1.8237739e+00   1.8476797e+00   1.7865901e+00   1.8140219e+00   1.8725584e+00
1.8500861e+00   1.8495575e+00   1.7689887e+00   1.9201765e+00   1.8047077e+00
1.7811896e+00   1.8835641e+00   1.7677312e+00   1.8606021e+00   1.8584991e+00
1.8616669e+00   1.8364481e+00   1.8171593e+00   1.7589955e+00   1.8194906e+00
1.8280965e+00   1.7710208e+00   1.7556459e+00   1.7550067e+00   1.8377556e+00
1.8370635e+00   1.8715783e+00   1.7905709e+00   1.8131664e+00   1.7604217e+00
1.9543541e+00   1.8377708e+00   1.8321400e+00   1.8032955e+00   1.8663695e+00
1.8500939e+00   1.8710268e+00   1.8206090e+00   1.8394912e+00   1.8781405e+00
1.8299592e+00;
   1.9895326e+00   1.8929861e+00   1.9742348e+00   1.9293984e+00   1.9945145e+00
  1.9588042e+00   1.9712694e+00   1.9833077e+00   1.9012440e+00   1.9709892e+00
 1.9514419e+00   1.9416532e+00   1.9105498e+00   1.9579321e+00   1.9741193e+00
1.9810243e+00   1.9956866e+00   1.9299403e+00   1.9652436e+00   1.9289905e+00
1.9044113e+00   1.9285459e+00   1.9267529e+00   1.9549719e+00   2.0534035e+00
```

```
1.9031528e+00   1.9612671e+00   1.8969161e+00   2.0189464e+00   1.8516501e+00
1.9450877e+00   1.8993857e+00   1.9015714e+00   1.9041930e+00   1.9639914e+00
2.0258220e+00   1.9661581e+00   1.9311889e+00   1.9367111e+00   1.9637036e+00
1.9184841e+00   2.0086422e+00   1.9394067e+00   1.9609494e+00   1.9917266e+00
1.8948709e+00   1.9390498e+00   1.9083208e+00   1.9382990e+00   1.8811746e+00;
   2.0632227e+00   2.0390968e+00   2.0710534e+00   2.0479394e+00   2.0164603e+00
  2.1234889e+00   2.0003264e+00   2.0418869e+00   2.0745443e+00   2.0333376e+00
 2.0474688e+00   2.0414926e+00   2.0313796e+00   2.0702328e+00   2.0045402e+00
2.0491671e+00   1.9799241e+00   2.0312036e+00   2.0426957e+00   2.0178446e+00
2.0825658e+00   1.9958174e+00   2.0366013e+00   2.0415630e+00   2.0035827e+00
2.0684712e+00   1.9999538e+00   2.0444414e+00   2.0555468e+00   2.0076043e+00
2.0107036e+00   2.0054324e+00   2.0620787e+00   2.0252881e+00   2.0572186e+00
2.0270698e+00   2.0046480e+00   2.0122470e+00   1.9711377e+00   2.0661254e+00
2.0631462e+00   2.0088802e+00   2.1065188e+00   2.0489733e+00   2.0088305e+00
2.0064049e+00   2.0315456e+00   1.9996614e+00   2.0032758e+00   1.9967175e+00;
   2.0836465e+00   2.0334276e+00   2.0515625e+00   2.0989617e+00   2.0181750e+00
  2.1043128e+00   2.0808559e+00   2.1071697e+00   2.0872565e+00   2.0132561e+00
 2.1159183e+00   2.0898765e+00   2.0996899e+00   2.0717184e+00   2.0452088e+00
2.0566133e+00   2.1033178e+00   2.0513799e+00   2.1041943e+00   2.0571066e+00
2.0273164e+00   2.0572086e+00   2.0253651e+00   2.0931239e+00   2.0330529e+00
2.0671380e+00   2.0660028e+00   2.0542891e+00   2.0403012e+00   2.0688370e+00
2.0848454e+00   2.0528385e+00   2.0140499e+00   2.0521044e+00   2.0751197e+00
2.0361262e+00   2.0290546e+00   2.1357194e+00   2.1294534e+00   2.0734515e+00
2.0365412e+00   2.1023843e+00   2.0612881e+00   2.0501618e+00   2.1046073e+00
2.0788597e+00   2.0690613e+00   2.1097677e+00   2.0850977e+00   2.0184771e+00;
   2.0126264e+00   2.0135462e+00   2.0347067e+00   2.0524927e+00   2.0776836e+00
  2.0552145e+00   2.0272971e+00   2.0870560e+00   2.0441728e+00   2.0595414e+00
 2.0513863e+00   2.0603987e+00   2.0355927e+00   2.0508116e+00   2.0344313e+00
2.0405313e+00   2.0519881e+00   2.0876370e+00   2.0258679e+00   2.0466059e+00
2.0799952e+00   2.0643826e+00   2.0714936e+00   2.0631355e+00   2.0724947e+00
2.0351723e+00   2.0565610e+00   2.0027937e+00   2.0927675e+00   2.0172832e+00
1.9809046e+00   2.0913788e+00   2.0872682e+00   2.0444279e+00   2.0580415e+00
2.0675242e+00   2.0828463e+00   2.0379267e+00   2.0563335e+00   2.0703511e+00
2.0357626e+00   2.0567154e+00   2.0788833e+00   2.0490365e+00   2.0678077e+00
2.0282735e+00   2.0501539e+00   2.0301337e+00   2.0416122e+00   2.0770962e+00;
   1.9514300e+00   1.9825022e+00   1.9656990e+00   1.9720984e+00   2.0168366e+00
  1.9642116e+00   1.9850065e+00   1.9650215e+00   2.0136013e+00   1.9587494e+00
 1.9820657e+00   2.0667104e+00   1.9452067e+00   2.0272442e+00   1.9988192e+00
1.9824952e+00   2.0006845e+00   1.9360085e+00   1.9468266e+00   2.0225514e+00
2.0017569e+00   1.9789572e+00   2.0094811e+00   2.0445435e+00   1.9383628e+00
1.9885468e+00   2.0160001e+00   1.9742970e+00   1.9540269e+00   1.9374173e+00
1.9686486e+00   2.0173284e+00   2.0181967e+00   2.0014018e+00   1.9978908e+00
2.0081807e+00   2.0396038e+00   1.9515167e+00   1.9642549e+00   1.9872170e+00
1.9759316e+00   1.9663630e+00   2.0444185e+00   1.9795954e+00   2.0164868e+00
2.0300929e+00   1.9717096e+00   2.0364355e+00   1.9561596e+00   1.9579055e+00];

 delay_1  delay_2   mu1_mean mu1_median   mu1_mode mu1_HDIlow mu1_HDIhigh
Vdet1 mu1_pcgtVdet1 mu1_ROPElow mu1_ROPEhigh mu1_pcInROPE
     -10        -5     1.8212     1.8212     1.8216     1.8119      1.8304
1.8440         0.00      1.8340       1.8540         0.36
     -10         0     1.8212     1.8212     1.8212     1.8117      1.8303
1.8440         0.00      1.8340       1.8540         0.41
     -10         5     1.8212     1.8212     1.8211     1.8118      1.8304
1.8440         0.00      1.8340       1.8540         0.39
```

-10	10	1.8213	1.8213	1.8218	1.8119	1.8304	1.8440	0.00	1.8340	1.8540	0.41
-10	15	1.8212	1.8212	1.8212	1.8119	1.8306	1.8440	0.00	1.8340	1.8540	0.37
-5	0	1.9445	1.9445	1.9446	1.9363	1.9525	2.0620	0.00	2.0520	2.0720	0.00
-5	5	1.9445	1.9445	1.9448	1.9364	1.9526	2.0620	0.00	2.0520	2.0720	0.00
-5	10	1.9444	1.9444	1.9443	1.9363	1.9524	2.0620	0.00	2.0520	2.0720	0.00
-5	15	1.9445	1.9445	1.9448	1.9364	1.9525	2.0620	0.00	2.0520	2.0720	0.00
0	5	2.0355	2.0355	2.0354	2.0289	2.0419	2.2540	0.00	2.2440	2.2640	0.00
0	10	2.0354	2.0354	2.0353	2.0289	2.0419	2.2540	0.00	2.2440	2.2640	0.00
0	15	2.0355	2.0355	2.0354	2.0290	2.0419	2.2540	0.00	2.2440	2.2640	0.00
5	10	2.0692	2.0692	2.0693	2.0636	2.0746	2.2920	0.00	2.2820	2.3020	0.00
5	15	2.0693	2.0693	2.0692	2.0638	2.0748	2.2920	0.00	2.2820	2.3020	0.00
10	15	2.0542	2.0542	2.0540	2.0485	2.0598	2.1730	0.00	2.1630	2.1830	0.00

delay_1	delay_2	mu2_mean	mu2_median	mu2_mode	mu2_HDIlow	mu2_HDIhigh	Vdet2	mu2_pcgtVdet2	mu2_ROPElow	mu2_ROPEhigh	mu2_pcInROPE
-10	-5	1.9444	1.9445	1.9447	1.9361	1.9524	2.0620	0.00	2.0520	2.0720	0.00
-10	0	2.0354	2.0354	2.0350	2.0289	2.0419	2.2540	0.00	2.2440	2.2640	0.00
-10	5	2.0693	2.0693	2.0693	2.0638	2.0748	2.2920	0.00	2.2820	2.3020	0.00
-10	10	2.0543	2.0543	2.0543	2.0487	2.0599	2.1730	0.00	2.1630	2.1830	0.00
-10	15	1.9899	1.9899	1.9900	1.9836	1.9961	2.0460	0.00	2.0360	2.0560	0.00
-5	0	2.0355	2.0354	2.0353	2.0290	2.0419	2.2540	0.00	2.2440	2.2640	0.00
-5	5	2.0693	2.0693	2.0692	2.0639	2.0749	2.2920	0.00	2.2820	2.3020	0.00
-5	10	2.0542	2.0542	2.0543	2.0485	2.0598	2.1730	0.00	2.1630	2.1830	0.00
-5	15	1.9899	1.9899	1.9897	1.9838	1.9962	2.0460	0.00	2.0360	2.0560	0.00
0	5	2.0693	2.0693	2.0692	2.0638	2.0748	2.2920	0.00	2.2820	2.3020	0.00
0	10	2.0542	2.0542	2.0542	2.0485	2.0597	2.1730	0.00	2.1630	2.1830	0.00
0	15	1.9900	1.9900	1.9895	1.9837	1.9960	2.0460	0.00	2.0360	2.0560	0.00
5	10	2.0543	2.0542	2.0543	2.0486	2.0598	2.1730	0.00	2.1630	2.1830	0.00
5	15	1.9900	1.9900	1.9900	1.9838	1.9963					

```
2.0460          0.00      2.0360       2.0560         0.00
      10       15     1.9899     1.9899     1.9899     1.9836      1.9961
2.0460          0.00      2.0360       2.0560         0.00

 delay_1  delay_2 muDiff_mean muDiff_median muDiff_mode muDiff_HDIlow
muDiff_HDIhigh muDiff_pcgtZero muDiff_ROPElow muDiff_ROPEhigh   pcInROPE
     -10       -5     -0.1232        -0.1232     -0.1235       -0.1354
-0.1107            0.00        -0.0100          0.0100       0.00
     -10        0     -0.2142        -0.2143     -0.2141       -0.2254
-0.2028            0.00        -0.0100          0.0100       0.00
     -10        5     -0.2481        -0.2481     -0.2480       -0.2590
-0.2374            0.00        -0.0100          0.0100       0.00
     -10       10     -0.2330        -0.2330     -0.2327       -0.2438
-0.2221            0.00        -0.0100          0.0100       0.00
     -10       15     -0.1687        -0.1687     -0.1682       -0.1800
-0.1576            0.00        -0.0100          0.0100       0.00
      -5        0     -0.0910        -0.0910     -0.0911       -0.1014
-0.0806            0.00        -0.0100          0.0100       0.00
      -5        5     -0.1249        -0.1249     -0.1249       -0.1347
-0.1151            0.00        -0.0100          0.0100       0.00
      -5       10     -0.1098        -0.1098     -0.1096       -0.1195
-0.0998            0.00        -0.0100          0.0100       0.00
      -5       15     -0.0455        -0.0454     -0.0448       -0.0557
-0.0354            0.00        -0.0100          0.0100       0.00
       0        5     -0.0338        -0.0339     -0.0341       -0.0423
-0.0253            0.00        -0.0100          0.0100       0.00
       0       10     -0.0189        -0.0189     -0.0183       -0.0274
-0.0103            0.00        -0.0100          0.0100       2.15
       0       15      0.0455         0.0455      0.0457        0.0366
0.0546          100.00        -0.0100          0.0100       0.00
       5       10      0.0150         0.0150      0.0150        0.0070
0.0227           99.98        -0.0100          0.0100      10.79
       5       15      0.0794         0.0794      0.0792        0.0712
0.0877          100.00        -0.0100          0.0100       0.00
      10       15      0.0643         0.0643      0.0641        0.0558
0.0726          100.00        -0.0100          0.0100       0.00

 delay_1  delay_2    nu_mean  nu_median    nu_mode  nu_HDIlow nu_HDIhigh
nuLog10_mean nuLog10_median nuLog10_mode nuLog10_HDIlow nuLog10_HDIhigh
     -10       -5    40.6801    31.9858    18.3087     4.9115   101.4050
1.5040         1.5050       1.5071         0.9164          2.0728
     -10        0    42.0814    33.6444    18.4798     5.3541   103.0080
1.5250         1.5269       1.5422         0.9582          2.0889
     -10        5    42.3627    33.5851    19.4817     4.9365   104.7210
1.5245         1.5261       1.5623         0.9579          2.1089
     -10       10    23.7196    16.1478     8.6628     3.0773    67.7906
1.2436         1.2081       1.1198         0.6748          1.8924
     -10       15    46.6981    38.0824    24.8665     6.3352   110.5310
1.5778         1.5807       1.5747         1.0265          2.1190
      -5        0    49.4281    40.9490    26.5668     6.8008   114.7870
1.6069         1.6122       1.6329         1.0606          2.1327
      -5        5    49.9496    41.3511    27.7108     6.4715   116.3930
1.6093         1.6165       1.6350         1.0565          2.1444
      -5       10    31.5434    23.1900    12.8335     3.8386    84.3531
```

```
1.3784         1.3653      1.3200        0.7851          1.9915
      -5       15    53.2243    44.9611    28.0056     7.7338   120.3080
1.6457         1.6528      1.6785        1.1257          2.1619
       0        5    50.8728    42.3900    26.2627     7.2248   117.8920
1.6209         1.6273      1.6488        1.0800          2.1469
       0       10    33.5674    25.1992    12.8008     4.3335    88.4114
1.4117         1.4014      1.3936        0.8256          2.0065
       0       15    54.5925    46.1672    31.0527     8.5567   123.0220
1.6590         1.6643      1.6817        1.1462          2.1645
       5       10    33.2875    24.7754    11.6990     3.8349    88.1038
1.4027         1.3940      1.4195        0.8022          2.0134
       5       15    55.0414    46.5860    30.1629     8.0263   123.8790
1.6618         1.6683      1.6885        1.1452          2.1731
      10       15    38.8858    30.3079    15.4779     4.8495    98.1284
1.4837         1.4816      1.5052        0.9088          2.0671

 delay_1  delay_2 effSz_mean effSz_median effSz_mode effSz_HDIlow effSz_HDIhigh
effSz_pcgtZero effSz_ROPElow effSz_ROPEhigh effSz_pcInROPE
     -10       -5    -2.8836      -2.8788    -2.8709      -3.3384       -2.4524
       0.0000       -0.1000         0.1000         0.0000
     -10        0    -5.4438      -5.4342    -5.4312      -6.1696       -4.7379
       0.0000       -0.1000         0.1000         0.0000
     -10        5    -6.6159      -6.6037    -6.5914      -7.5011       -5.7309
       0.0000       -0.1000         0.1000         0.0000
     -10       10    -6.3829      -6.3473    -6.2974      -7.4076       -5.4004
       0.0000       -0.1000         0.1000         0.0000
     -10       15    -4.3253      -4.3205    -4.3272      -4.9237       -3.7521
       0.0000       -0.1000         0.1000         0.0000
      -5        0    -2.5166      -2.5145    -2.5099      -2.9208       -2.1240
       0.0000       -0.1000         0.1000         0.0000
      -5        5    -3.6608      -3.6568    -3.6508      -4.1736       -3.1504
       0.0000       -0.1000         0.1000         0.0000
      -5       10    -3.2558      -3.2456    -3.2328      -3.7909       -2.7437
       0.0000       -0.1000         0.1000         0.0000
      -5       15    -1.2759      -1.2750    -1.2652      -1.5890       -0.9559
       0.0000       -0.1000         0.1000         0.0000
       0        5    -1.1430      -1.1422    -1.1343      -1.4584       -0.8333
       0.0000       -0.1000         0.1000         0.0000
       0       10    -0.6400      -0.6386    -0.6302      -0.9425       -0.3390
       0.0010       -0.1000         0.1000         0.0190
       0       15     1.4520       1.4513     1.4478       1.1211        1.7685
     100.0000       -0.1000         0.1000         0.0000
       5       10     0.5541       0.5537     0.5500       0.2575        0.8497
      99.9825       -0.1000         0.1000         0.1375
       5       15     2.7396       2.7380     2.7396       2.3416        3.1575
     100.0000       -0.1000         0.1000         0.0000
      10       15     2.2152       2.2113     2.1897       1.8285        2.6075
     100.0000       -0.1000         0.1000         0.0000

 delay_1  delay_2 sigma1_mean sigma1_median sigma1_mode sigma1_HDIlow
sigma1_HDIhigh sigma2_mean sigma2_median  sigma2_mode sigma2_HDIlow
sigma2_HDIhigh sigmaDiff_mean sigmaDiff_median sigmaDiff_mode sigmaDiff_HDIlow
sigmaDiff_HDIhigh sigmaDiff_pcgtZero
     -10       -5    0.045721      0.045588    0.045234      0.038588
```

0.053108 0.039722 0.039576 0.039230 0.033754 0.045940
0.005999 0.005987 0.006373 -0.003101 0.015095
90.40
-10 0 0.045818 0.045669 0.045265 0.038790
0.053196 0.031843 0.031718 0.031360 0.027172 0.036788
0.013975 0.013895 0.013637 0.005574 0.022359
99.95
-10 5 0.045837 0.045683 0.045379 0.038838
0.053338 0.026992 0.026889 0.026663 0.022939 0.031206
0.018845 0.018740 0.018685 0.010982 0.027102
100.00
-10 10 0.044409 0.044324 0.044356 0.036666
0.052254 0.026779 0.026733 0.026762 0.021805 0.031791
0.017630 0.017525 0.016998 0.009698 0.025955
100.00
-10 15 0.046044 0.045872 0.045606 0.039016
0.053287 0.030625 0.030492 0.030114 0.026134 0.035225
0.015419 0.015332 0.015307 0.007211 0.023855
99.99
-5 0 0.040016 0.039869 0.039847 0.034306
0.046291 0.031980 0.031855 0.031868 0.027366 0.036879
0.008035 0.007984 0.008208 0.000463 0.015587
98.32
-5 5 0.040015 0.039866 0.039240 0.034161
0.046175 0.027133 0.027029 0.026655 0.023117 0.031234
0.012881 0.012797 0.012962 0.005685 0.019980
99.99
-5 10 0.039310 0.039183 0.039060 0.033057
0.045717 0.027305 0.027249 0.026990 0.022574 0.032162
0.012006 0.011922 0.011621 0.004768 0.019352
99.95
-5 15 0.040069 0.039909 0.039664 0.034281
0.046149 0.030697 0.030570 0.030388 0.026313 0.035280
0.009372 0.009310 0.009302 0.001857 0.016678
99.47
0 5 0.031991 0.031860 0.031791 0.027295
0.036764 0.027140 0.027027 0.026632 0.023156 0.031248
0.004851 0.004831 0.005097 -0.001284 0.011028
94.15
0 10 0.031553 0.031447 0.031395 0.026748
0.036570 0.027427 0.027360 0.027034 0.022767 0.032157
0.004126 0.004096 0.003890 -0.002281 0.010414
90.23
0 15 0.032067 0.031938 0.031708 0.027498
0.036914 0.030708 0.030580 0.030527 0.026315 0.035284
0.001358 0.001349 0.001117 -0.005035 0.007944
66.17
5 10 0.026715 0.026630 0.026540 0.022526
0.030984 0.027383 0.027327 0.027261 0.022657 0.032185
-0.000667 -0.000662 -0.000841 -0.006599 0.005153
41.12
5 15 0.027208 0.027098 0.026911 0.023193
0.031224 0.030717 0.030584 0.030346 0.026355 0.035301
-0.003510 -0.003479 -0.003264 -0.009502 0.002494

12.30
10 15 0.027676 0.027607 0.027592 0.023181
0.032360 0.030458 0.030337 0.030046 0.025975 0.035140
-0.002781 -0.002759 -0.002669 -0.009042 0.003367
18.74

****
Results for noise_window for additive noise in activation used in simulations:
[-10 ms 10 ms]; 10 random sample per segment.
****
t_l_max_1 = [5.0551920e-02 5.0375920e-02 5.0615920e-02 5.0895920e-02
5.0871920e-02 5.0727920e-02 5.0807920e-02 5.0863920e-02 5.0831920e-02
5.0871920e-02 5.0567920e-02 5.1143920e-02 5.0999920e-02 5.0879920e-02
5.0799920e-02 5.0927920e-02 5.0879920e-02 5.0559920e-02 5.0639920e-02
5.0759920e-02 5.0791920e-02 5.0599920e-02 5.0551920e-02 5.0639920e-02
5.0607920e-02 5.0815920e-02 5.0647920e-02 5.0639920e-02 5.0863920e-02
5.0511920e-02 5.0831920e-02 5.0471920e-02 5.0543920e-02 5.0663920e-02
5.0679920e-02 5.1111920e-02 5.0647920e-02 5.0743920e-02 5.0943920e-02
5.0727920e-02 5.0751920e-02 5.0911920e-02 5.0935920e-02 5.0935920e-02
5.0871920e-02 5.0751920e-02 5.1007920e-02 5.0575920e-02 5.0551920e-02
5.0607920e-02;
5.1903920e-02 5.2231920e-02 5.2191920e-02 5.1967920e-02 5.2191920e-02
5.2191920e-02 5.1711920e-02 5.1895920e-02 5.2127920e-02 5.2015920e-02
5.2135920e-02 5.2191920e-02 5.1863920e-02 5.2111920e-02 5.2383920e-02
5.2167920e-02 5.1775920e-02 5.1967920e-02 5.2007920e-02 5.1999920e-02
5.2151920e-02 5.2039920e-02 5.2047920e-02 5.1967920e-02 5.1855920e-02
5.2143920e-02 5.1927920e-02 5.1959920e-02 5.1887920e-02 5.2335920e-02
5.1999920e-02 5.1831920e-02 5.2255920e-02 5.2207920e-02 5.2039920e-02
5.1991920e-02 5.2023920e-02 5.2127920e-02 5.2295920e-02 5.2223920e-02
5.1767920e-02 5.1911920e-02 5.2199920e-02 5.2135920e-02 5.1575920e-02
5.2063920e-02 5.1839920e-02 5.1975920e-02 5.2359920e-02 5.2183920e-02;
5.3975920e-02 5.4079920e-02 5.4055920e-02 5.3863920e-02 5.3655920e-02
5.3823920e-02 5.3919920e-02 5.3895920e-02 5.3735920e-02 5.4103920e-02
5.3903920e-02 5.3983920e-02 5.3959920e-02 5.4103920e-02 5.3799920e-02
5.4031920e-02 5.3903920e-02 5.3991920e-02 5.3959920e-02 5.4159920e-02
5.3975920e-02 5.3775920e-02 5.4007920e-02 5.3991920e-02 5.4127920e-02
5.3975920e-02 5.4007920e-02 5.3967920e-02 5.4063920e-02 5.3879920e-02
5.4047920e-02 5.4167920e-02 5.3815920e-02 5.3775920e-02 5.4079920e-02
5.3815920e-02 5.3799920e-02 5.3743920e-02 5.3743920e-02 5.4055920e-02
5.4103920e-02 5.3735920e-02 5.3943920e-02 5.3711920e-02 5.4007920e-02
5.3951920e-02 5.4023920e-02 5.3831920e-02 5.4047920e-02 5.4071920e-02;
5.6487920e-02 5.6583920e-02 5.6623920e-02 5.6383920e-02 5.6519920e-02
5.6399920e-02 5.6751920e-02 5.6263920e-02 5.6439920e-02 5.6367920e-02
5.6415920e-02 5.6231920e-02 5.6567920e-02 5.6503920e-02 5.6471920e-02
5.6415920e-02 5.6535920e-02 5.6735920e-02 5.6807920e-02 5.6463920e-02
5.6751920e-02 5.6383920e-02 5.6375920e-02 5.6543920e-02 5.6471920e-02
5.6495920e-02 5.6551920e-02 5.6327920e-02 5.6367920e-02 5.6615920e-02
5.6711920e-02 5.6559920e-02 5.6607920e-02 5.6279920e-02 5.6463920e-02
5.6583920e-02 5.6647920e-02 5.6511920e-02 5.6399920e-02 5.6671920e-02
5.6231920e-02 5.6535920e-02 5.6511920e-02 5.6447920e-02 5.6639920e-02
5.6623920e-02 5.6567920e-02 5.6559920e-02 5.6535920e-02 5.6679920e-02;
5.9871920e-02 5.9671920e-02 5.9647920e-02 5.9503920e-02 5.9599920e-02
5.9623920e-02 5.9535920e-02 5.9847920e-02 5.9279920e-02 5.9791920e-02

```
 5.9367920e-02   5.9551920e-02   5.9631920e-02   6.0039920e-02   5.9775920e-02
5.9703920e-02   5.9375920e-02   5.9543920e-02   5.9335920e-02   5.9639920e-02
5.9631920e-02   5.9599920e-02   5.9431920e-02   5.9807920e-02   5.9823920e-02
5.9823920e-02   5.9511920e-02   5.9735920e-02   5.9855920e-02   5.9807920e-02
5.9399920e-02   5.9623920e-02   5.9759920e-02   5.9591920e-02   5.9671920e-02
5.9535920e-02   5.9815920e-02   5.9567920e-02   5.9639920e-02   5.9567920e-02
5.9639920e-02   5.9559920e-02   5.9775920e-02   5.9743920e-02   5.9527920e-02
5.9535920e-02   5.9663920e-02   5.9615920e-02   5.9543920e-02   5.9727920e-02;
   6.2567920e-02   6.2647920e-02   6.2919920e-02   6.2879920e-02   6.2727920e-02
  6.2855920e-02   6.2855920e-02   6.2895920e-02   6.2679920e-02   6.2823920e-02
 6.3039920e-02   6.2951920e-02   6.2775920e-02   6.3015920e-02   6.3207920e-02
6.3271920e-02   6.2983920e-02   6.2671920e-02   6.2791920e-02   6.2887920e-02
6.2863920e-02   6.2943920e-02   6.3119920e-02   6.2951920e-02   6.2871920e-02
6.2911920e-02   6.2823920e-02   6.2719920e-02   6.2743920e-02   6.2951920e-02
6.2895920e-02   6.2927920e-02   6.2991920e-02   6.2719920e-02   6.2807920e-02
6.2863920e-02   6.2783920e-02   6.2407920e-02   6.3167920e-02   6.3079920e-02
6.2943920e-02   6.2711920e-02   6.3039920e-02   6.2951920e-02   6.2463920e-02
6.2639920e-02   6.2751920e-02   6.2863920e-02   6.2903920e-02   6.2679920e-02];
t_u_max_1 =     [3.8943920e-02   3.8359920e-02   3.8543920e-02   3.8799920e-02
3.9031920e-02   3.8663920e-02   3.8903920e-02   3.8527920e-02   3.9039920e-02
3.8647920e-02   3.8743920e-02   3.9023920e-02   3.8975920e-02   3.8663920e-02
3.8991920e-02   3.9015920e-02   3.8999920e-02   3.8639920e-02   3.8583920e-02
3.8767920e-02   3.8711920e-02   3.8687920e-02   3.8495920e-02   3.8687920e-02
3.8607920e-02   3.8783920e-02   3.8495920e-02   3.8847920e-02   3.8887920e-02
3.8551920e-02   3.8879920e-02   3.8679920e-02   3.8551920e-02   3.8343920e-02
3.8831920e-02   3.9167920e-02   3.8759920e-02   3.8767920e-02   3.8783920e-02
3.8855920e-02   3.8743920e-02   3.8839920e-02   3.8967920e-02   3.9127920e-02
3.8663920e-02   3.8703920e-02   3.9327920e-02   3.8695920e-02   3.8679920e-02
3.8599920e-02;
   4.0119920e-02   4.0487920e-02   4.0575920e-02   4.0295920e-02   4.0527920e-02
  4.0511920e-02   3.9975920e-02   4.0279920e-02   4.0343920e-02   4.0423920e-02
 4.0343920e-02   4.0455920e-02   4.0175920e-02   4.0527920e-02   4.0679920e-02
4.0407920e-02   4.0199920e-02   4.0223920e-02   4.0223920e-02   4.0471920e-02
4.0479920e-02   4.0215920e-02   4.0311920e-02   4.0359920e-02   4.0143920e-02
4.0319920e-02   4.0207920e-02   4.0191920e-02   4.0279920e-02   4.0623920e-02
4.0295920e-02   4.0135920e-02   4.0663920e-02   4.0535920e-02   4.0271920e-02
4.0407920e-02   4.0247920e-02   4.0551920e-02   4.0543920e-02   4.0559920e-02
4.0023920e-02   4.0327920e-02   4.0471920e-02   4.0479920e-02   3.9895920e-02
4.0415920e-02   4.0127920e-02   4.0271920e-02   4.0639920e-02   4.0495920e-02;
   4.2543920e-02   4.2695920e-02   4.2631920e-02   4.2439920e-02   4.2255920e-02
  4.2335920e-02   4.2511920e-02   4.2439920e-02   4.2335920e-02   4.2647920e-02
 4.2487920e-02   4.2567920e-02   4.2447920e-02   4.2703920e-02   4.2351920e-02
4.2559920e-02   4.2463920e-02   4.2583920e-02   4.2551920e-02   4.2695920e-02
4.2463920e-02   4.2367920e-02   4.2543920e-02   4.2551920e-02   4.2703920e-02
4.2551920e-02   4.2591920e-02   4.2543920e-02   4.2591920e-02   4.2503920e-02
4.2607920e-02   4.2727920e-02   4.2399920e-02   4.2359920e-02   4.2639920e-02
4.2423920e-02   4.2439920e-02   4.2311920e-02   4.2247920e-02   4.2639920e-02
4.2687920e-02   4.2239920e-02   4.2543920e-02   4.2311920e-02   4.2623920e-02
4.2487920e-02   4.2631920e-02   4.2407920e-02   4.2591920e-02   4.2663920e-02;
   4.5183920e-02   4.5311920e-02   4.5327920e-02   4.5087920e-02   4.5239920e-02
  4.5119920e-02   4.5487920e-02   4.4991920e-02   4.5143920e-02   4.5071920e-02
 4.5175920e-02   4.4903920e-02   4.5263920e-02   4.5239920e-02   4.5135920e-02
4.5127920e-02   4.5223920e-02   4.5439920e-02   4.5535920e-02   4.5183920e-02
4.5487920e-02   4.5095920e-02   4.5103920e-02   4.5215920e-02   4.5191920e-02
```

```
4.5159920e-02   4.5247920e-02   4.5031920e-02   4.5119920e-02   4.5351920e-02
4.5391920e-02   4.5255920e-02   4.5351920e-02   4.4991920e-02   4.5143920e-02
4.5303920e-02   4.5375920e-02   4.5199920e-02   4.5167920e-02   4.5407920e-02
4.4911920e-02   4.5279920e-02   4.5255920e-02   4.5127920e-02   4.5367920e-02
4.5351920e-02   4.5295920e-02   4.5303920e-02   4.5287920e-02   4.5375920e-02;
   4.8639920e-02   4.8439920e-02   4.8343920e-02   4.8263920e-02   4.8343920e-02
  4.8415920e-02   4.8327920e-02   4.8623920e-02   4.8055920e-02   4.8495920e-02
 4.8159920e-02   4.8327920e-02   4.8391920e-02   4.8831920e-02   4.8567920e-02
4.8455920e-02   4.8127920e-02   4.8287920e-02   4.8063920e-02   4.8399920e-02
4.8335920e-02   4.8335920e-02   4.8175920e-02   4.8575920e-02   4.8543920e-02
4.8583920e-02   4.8247920e-02   4.8527920e-02   4.8607920e-02   4.8567920e-02
4.8183920e-02   4.8383920e-02   4.8503920e-02   4.8359920e-02   4.8431920e-02
4.8303920e-02   4.8575920e-02   4.8359920e-02   4.8423920e-02   4.8327920e-02
4.8383920e-02   4.8359920e-02   4.8503920e-02   4.8527920e-02   4.8319920e-02
4.8223920e-02   4.8399920e-02   4.8335920e-02   4.8311920e-02   4.8447920e-02;
   5.1199920e-02   5.1327920e-02   5.1615920e-02   5.1535920e-02   5.1423920e-02
  5.1551920e-02   5.1567920e-02   5.1535920e-02   5.1375920e-02   5.1479920e-02
 5.1719920e-02   5.1655920e-02   5.1423920e-02   5.1687920e-02   5.1903920e-02
5.1967920e-02   5.1695920e-02   5.1375920e-02   5.1439920e-02   5.1527920e-02
5.1527920e-02   5.1607920e-02   5.1823920e-02   5.1615920e-02   5.1543920e-02
5.1583920e-02   5.1495920e-02   5.1415920e-02   5.1439920e-02   5.1607920e-02
5.1615920e-02   5.1583920e-02   5.1671920e-02   5.1367920e-02   5.1503920e-02
5.1527920e-02   5.1439920e-02   5.1071920e-02   5.1871920e-02   5.1743920e-02
5.1647920e-02   5.1399920e-02   5.1703920e-02   5.1663920e-02   5.1159920e-02
5.1327920e-02   5.1463920e-02   5.1519920e-02   5.1551920e-02   5.1343920e-02];
l_max_1 =       [8.5123145e-02   8.5076851e-02   8.5064157e-02   8.5053873e-02
8.5054531e-02   8.5028980e-02   8.5072607e-02   8.5010023e-02   8.5077769e-02
8.5031399e-02   8.5051800e-02   8.5011406e-02   8.5043814e-02   8.5034417e-02
8.5052364e-02   8.5067921e-02   8.5067362e-02   8.5078326e-02   8.5039284e-02
8.5066537e-02   8.5025023e-02   8.5098058e-02   8.5063158e-02   8.5056166e-02
8.5038553e-02   8.5067010e-02   8.5076578e-02   8.5078187e-02   8.5048019e-02
8.5028324e-02   8.5036432e-02   8.5097347e-02   8.5044326e-02   8.5008190e-02
8.5079451e-02   8.5041697e-02   8.5074099e-02   8.5050456e-02   8.5010515e-02
8.5025462e-02   8.5065837e-02   8.5020772e-02   8.5060544e-02   8.5061770e-02
8.5033927e-02   8.5064374e-02   8.5080527e-02   8.5050956e-02   8.5095989e-02
8.5101663e-02;
   8.5208099e-02   8.5199376e-02   8.5229904e-02   8.5203235e-02   8.5220569e-02
  8.5206102e-02   8.5214488e-02   8.5253489e-02   8.5216766e-02   8.5244442e-02
 8.5214462e-02   8.5208788e-02   8.5233087e-02   8.5234613e-02   8.5205200e-02
8.5225454e-02   8.5240593e-02   8.5221688e-02   8.5200545e-02   8.5233392e-02
8.5249966e-02   8.5206281e-02   8.5213341e-02   8.5262842e-02   8.5254491e-02
8.5174717e-02   8.5210253e-02   8.5201729e-02   8.5237721e-02   8.5203853e-02
8.5235146e-02   8.5229259e-02   8.5252899e-02   8.5224162e-02   8.5241083e-02
8.5253262e-02   8.5183315e-02   8.5218689e-02   8.5204774e-02   8.5214606e-02
8.5217811e-02   8.5226976e-02   8.5189927e-02   8.5237267e-02   8.5222148e-02
8.5245809e-02   8.5211661e-02   8.5221202e-02   8.5237645e-02   8.5211451e-02;
   8.5325557e-02   8.5311893e-02   8.5300396e-02   8.5332575e-02   8.5319633e-02
  8.5309897e-02   8.5320060e-02   8.5311646e-02   8.5318869e-02   8.5326344e-02
 8.5305078e-02   8.5322029e-02   8.5308527e-02   8.5323184e-02   8.5312044e-02
8.5313499e-02   8.5288109e-02   8.5317612e-02   8.5307967e-02   8.5307269e-02
8.5250734e-02   8.5278721e-02   8.5300197e-02   8.5310408e-02   8.5284664e-02
8.5323179e-02   8.5311761e-02   8.5311938e-02   8.5310711e-02   8.5331925e-02
8.5327173e-02   8.5327348e-02   8.5296255e-02   8.5312729e-02   8.5315151e-02
8.5308035e-02   8.5323872e-02   8.5303165e-02   8.5310471e-02   8.5329206e-02
```

8.5342222e-02   8.5296076e-02   8.5319253e-02   8.5326496e-02   8.5318522e-02
8.5295592e-02   8.5276811e-02   8.5307698e-02   8.5311036e-02   8.5284027e-02;
8.5245877e-02   8.5199768e-02   8.5180125e-02   8.5234024e-02   8.5220676e-02
8.5163611e-02   8.5241455e-02   8.5211753e-02   8.5191955e-02   8.5240728e-02
8.5221217e-02   8.5216422e-02   8.5218433e-02   8.5187118e-02   8.5212680e-02
8.5228078e-02   8.5198496e-02   8.5259991e-02   8.5184604e-02   8.5194834e-02
8.5222385e-02   8.5200085e-02   8.5275378e-02   8.5216428e-02   8.5252691e-02
8.5199127e-02   8.5198365e-02   8.5216789e-02   8.5237634e-02   8.5219531e-02
8.5208090e-02   8.5214168e-02   8.5192363e-02   8.5233830e-02   8.5213670e-02
8.5181607e-02   8.5191437e-02   8.5198896e-02   8.5209340e-02   8.5216006e-02
8.5219561e-02   8.5233384e-02   8.5207589e-02   8.5186013e-02   8.5205110e-02
8.5158317e-02   8.5265230e-02   8.5212549e-02   8.5225429e-02   8.5190444e-02;
8.4973462e-02   8.4970820e-02   8.5032028e-02   8.5040676e-02   8.5031077e-02
8.4988631e-02   8.5018590e-02   8.4977612e-02   8.5022089e-02   8.4989114e-02
8.5038977e-02   8.4988822e-02   8.5013445e-02   8.4985470e-02   8.5005129e-02
8.4985023e-02   8.4959193e-02   8.4945678e-02   8.4958061e-02   8.4983288e-02
8.4934231e-02   8.4958281e-02   8.5010247e-02   8.4969077e-02   8.4948319e-02
8.4976919e-02   8.5006583e-02   8.4999118e-02   8.4959062e-02   8.5001579e-02
8.5043473e-02   8.5000518e-02   8.4972012e-02   8.4974422e-02   8.5010375e-02
8.5029275e-02   8.5004753e-02   8.5016744e-02   8.4995909e-02   8.5043346e-02
8.4971903e-02   8.5009046e-02   8.4953261e-02   8.5015753e-02   8.5049415e-02
8.4988484e-02   8.4992423e-02   8.4969641e-02   8.4977112e-02   8.4995753e-02;
8.4761495e-02   8.4766630e-02   8.4792709e-02   8.4720788e-02   8.4775100e-02
8.4775141e-02   8.4800717e-02   8.4701313e-02   8.4770065e-02   8.4732858e-02
8.4753777e-02   8.4779184e-02   8.4750150e-02   8.4755594e-02   8.4771560e-02
8.4767465e-02   8.4786462e-02   8.4788814e-02   8.4705805e-02   8.4700156e-02
8.4755537e-02   8.4758919e-02   8.4751088e-02   8.4741945e-02   8.4776028e-02
8.4762535e-02   8.4781577e-02   8.4783944e-02   8.4771284e-02   8.4719452e-02
8.4806567e-02   8.4725299e-02   8.4775602e-02   8.4737210e-02   8.4795564e-02
8.4756103e-02   8.4738975e-02   8.4757281e-02   8.4776787e-02   8.4758465e-02
8.4767269e-02   8.4770987e-02   8.4765373e-02   8.4771170e-02   8.4798801e-02
8.4749802e-02   8.4796178e-02   8.4722426e-02   8.4730828e-02   8.4758289e-02];
u_max_1 =   [1.8493782e+00   1.8354522e+00   1.8257233e+00   1.8189560e+00
1.8169008e+00   1.7870253e+00   1.8197034e+00   1.7953188e+00   1.8269762e+00
1.8126612e+00   1.8101294e+00   1.7689792e+00   1.7986241e+00   1.8013810e+00
1.8052764e+00   1.8267590e+00   1.8169549e+00   1.8481648e+00   1.8075077e+00
1.8339455e+00   1.8014480e+00   1.8474307e+00   1.8249968e+00   1.8232144e+00
1.8073373e+00   1.8239092e+00   1.8365390e+00   1.8265144e+00   1.8091385e+00
1.7954672e+00   1.7982567e+00   1.8465931e+00   1.8148808e+00   1.7912323e+00
1.8310365e+00   1.7969491e+00   1.8243745e+00   1.8151766e+00   1.7883442e+00
1.7915421e+00   1.8324942e+00   1.7938615e+00   1.8101318e+00   1.8098371e+00
1.8014770e+00   1.8211374e+00   1.8195163e+00   1.8134296e+00   1.8461206e+00
1.8505408e+00;
1.9801915e+00   1.9542964e+00   1.9825034e+00   1.9605226e+00   1.9708108e+00
1.9571424e+00   1.9712558e+00   1.9977248e+00   1.9778311e+00   1.9762018e+00
1.9691437e+00   1.9693280e+00   1.9873104e+00   1.9740343e+00   1.9574872e+00
1.9756359e+00   1.9838750e+00   1.9710731e+00   1.9608012e+00   1.9858326e+00
1.9868276e+00   1.9682709e+00   1.9680832e+00   1.9971259e+00   2.0084601e+00
1.9350015e+00   1.9735982e+00   1.9604078e+00   1.9924019e+00   1.9613731e+00
1.9812705e+00   2.0046093e+00   1.9969252e+00   1.9653004e+00   1.9944079e+00
1.9906341e+00   1.9538712e+00   1.9741757e+00   1.9565357e+00   1.9621310e+00
1.9604491e+00   1.9764650e+00   1.9460772e+00   1.9792009e+00   1.9845780e+00
1.9892666e+00   1.9680871e+00   1.9674538e+00   1.9756340e+00   1.9624306e+00;
2.1095467e+00   2.1176375e+00   2.1042463e+00   2.1165792e+00   2.1300710e+00

```
  2.1088072e+00   2.1267570e+00   2.1256732e+00   2.1182570e+00   2.1006614e+00
 2.1228400e+00   2.1236440e+00   2.1044861e+00   2.1179749e+00   2.1122211e+00
2.0897106e+00   2.1068168e+00   2.1323147e+00   2.1067127e+00   2.1086968e+00
2.0752804e+00   2.1133105e+00   2.1097301e+00   2.1070354e+00   2.0803811e+00
2.1142930e+00   2.1239466e+00   2.1196489e+00   2.1075631e+00   2.1138401e+00
2.1233078e+00   2.1248850e+00   2.1017508e+00   2.1309717e+00   2.1125447e+00
2.1179173e+00   2.1358140e+00   2.1026282e+00   2.1026459e+00   2.1181752e+00
2.1175126e+00   2.1023236e+00   2.1129423e+00   2.1188485e+00   2.1234070e+00
2.1078332e+00   2.1036220e+00   2.1151645e+00   2.1034976e+00   2.1004287e+00;
   2.1597725e+00   2.1530468e+00   2.1423212e+00   2.1692161e+00   2.1660966e+00
  2.1400099e+00   2.1605165e+00   2.1612226e+00   2.1556184e+00   2.1666524e+00
 2.1593256e+00   2.1543958e+00   2.1487003e+00   2.1507111e+00   2.1441313e+00
2.1586300e+00   2.1479337e+00   2.1705631e+00   2.1393102e+00   2.1482905e+00
2.1667616e+00   2.1486023e+00   2.1756200e+00   2.1448872e+00   2.1677465e+00
2.1459606e+00   2.1403635e+00   2.1477500e+00   2.1614233e+00   2.1621760e+00
2.1411838e+00   2.1483003e+00   2.1581672e+00   2.1673357e+00   2.1480175e+00
2.1416377e+00   2.1538162e+00   2.1387703e+00   2.1601612e+00   2.1453261e+00
2.1546145e+00   2.1725318e+00   2.1532105e+00   2.1372948e+00   2.1542942e+00
2.1473158e+00   2.1702754e+00   2.1645012e+00   2.1606850e+00   2.1456141e+00;
   2.1026014e+00   2.0984920e+00   2.1142043e+00   2.1237645e+00   2.1171344e+00
  2.1117834e+00   2.1217649e+00   2.1014348e+00   2.1246773e+00   2.1062988e+00
 2.1309120e+00   2.1069788e+00   2.1169440e+00   2.1049260e+00   2.1162793e+00
2.1041634e+00   2.0980702e+00   2.0920202e+00   2.0887962e+00   2.0974618e+00
2.0860671e+00   2.0957311e+00   2.1119553e+00   2.1028186e+00   2.0866753e+00
2.0963120e+00   2.1085496e+00   2.1076738e+00   2.0974077e+00   2.1116100e+00
2.1269678e+00   2.1073275e+00   2.0969257e+00   2.1017954e+00   2.1069007e+00
2.1198115e+00   2.1127550e+00   2.1193952e+00   2.1106963e+00   2.1279518e+00
2.0995382e+00   2.1139722e+00   2.0878041e+00   2.1178122e+00   2.1265196e+00
2.1011912e+00   2.1073358e+00   2.0935176e+00   2.1059502e+00   2.1086084e+00;
   2.0141286e+00   2.0151997e+00   2.0302796e+00   1.9930849e+00   2.0171487e+00
  2.0195584e+00   2.0323664e+00   1.9896364e+00   2.0187520e+00   2.0001420e+00
 2.0070287e+00   2.0189240e+00   2.0095169e+00   2.0127512e+00   2.0188658e+00
2.0092888e+00   2.0233522e+00   2.0258691e+00   1.9892557e+00   1.9847167e+00
2.0128914e+00   2.0116667e+00   2.0014113e+00   2.0063153e+00   2.0224750e+00
2.0115343e+00   2.0195448e+00   2.0241436e+00   2.0136161e+00   1.9926059e+00
2.0327502e+00   1.9985042e+00   2.0166162e+00   2.0050081e+00   2.0285868e+00
2.0112060e+00   2.0037431e+00   2.0105705e+00   2.0205577e+00   2.0120376e+00
2.0170424e+00   2.0215729e+00   2.0114689e+00   2.0146123e+00   2.0337094e+00
2.0054575e+00   2.0308939e+00   1.9961176e+00   1.9986560e+00   2.0126615e+00];
t_l_max_2 = [5.0479920e-02   5.0751920e-02   5.0575920e-02   5.0815920e-02
5.0879920e-02   5.0639920e-02   5.0735920e-02   5.0799920e-02   5.0463920e-02
5.0511920e-02   5.0591920e-02   5.0639920e-02   5.0591920e-02   5.0431920e-02
5.0815920e-02   5.0575920e-02   5.0927920e-02   5.0591920e-02   5.0735920e-02
5.0911920e-02   5.0687920e-02   5.0415920e-02   5.0735920e-02   5.0655920e-02
5.0559920e-02   5.0559920e-02   5.0735920e-02   5.0399920e-02   5.0575920e-02
5.0719920e-02   5.0735920e-02   5.0911920e-02   5.0655920e-02   5.0927920e-02
5.0799920e-02   5.0527920e-02   5.0927920e-02   5.0559920e-02   5.0767920e-02
5.0831920e-02   5.0287920e-02   5.0575920e-02   5.0927920e-02   5.0831920e-02
5.0751920e-02   5.0847920e-02   5.0991920e-02   5.1007920e-02   5.0991920e-02
5.0815920e-02;
   5.1951920e-02   5.1951920e-02   5.2287920e-02   5.2319920e-02   5.1919920e-02
  5.2207920e-02   5.1951920e-02   5.2063920e-02   5.1807920e-02   5.2079920e-02
 5.2351920e-02   5.2015920e-02   5.1855920e-02   5.2079920e-02   5.1615920e-02
5.1967920e-02   5.1967920e-02   5.2207920e-02   5.2063920e-02   5.1887920e-02
```

```
5.2191920e-02   5.2335920e-02   5.1903920e-02   5.2127920e-02   5.1951920e-02
5.1903920e-02   5.1807920e-02   5.2127920e-02   5.2047920e-02   5.2015920e-02
5.1967920e-02   5.2063920e-02   5.1919920e-02   5.1919920e-02   5.2271920e-02
5.2047920e-02   5.2191920e-02   5.2095920e-02   5.2223920e-02   5.2159920e-02
5.2127920e-02   5.2015920e-02   5.1855920e-02   5.1967920e-02   5.2255920e-02
5.1935920e-02   5.1983920e-02   5.2047920e-02   5.2207920e-02   5.1903920e-02;
   5.4031920e-02   5.4143920e-02   5.3983920e-02   5.3983920e-02   5.3903920e-02
  5.3871920e-02   5.4031920e-02   5.4015920e-02   5.4239920e-02   5.3983920e-02
 5.3791920e-02   5.4143920e-02   5.3903920e-02   5.3839920e-02   5.4047920e-02
5.4031920e-02   5.3583920e-02   5.3983920e-02   5.3887920e-02   5.3727920e-02
5.3807920e-02   5.4303920e-02   5.3855920e-02   5.3967920e-02   5.3855920e-02
5.3871920e-02   5.3759920e-02   5.4015920e-02   5.3823920e-02   5.3791920e-02
5.4015920e-02   5.4143920e-02   5.3919920e-02   5.4031920e-02   5.3887920e-02
5.3823920e-02   5.3871920e-02   5.3743920e-02   5.3743920e-02   5.3935920e-02
5.3551920e-02   5.3839920e-02   5.3999920e-02   5.3775920e-02   5.3967920e-02
5.3903920e-02   5.4015920e-02   5.3823920e-02   5.3983920e-02   5.4223920e-02;
   5.6351920e-02   5.6463920e-02   5.6367920e-02   5.6591920e-02   5.6367920e-02
  5.6207920e-02   5.6399920e-02   5.6767920e-02   5.6751920e-02   5.6687920e-02
 5.6431920e-02   5.6671920e-02   5.6511920e-02   5.6255920e-02   5.6527920e-02
5.6463920e-02   5.6591920e-02   5.6319920e-02   5.6543920e-02   5.6479920e-02
5.6447920e-02   5.6495920e-02   5.6495920e-02   5.6511920e-02   5.6751920e-02
5.6639920e-02   5.6479920e-02   5.6399920e-02   5.6287920e-02   5.6543920e-02
5.6591920e-02   5.6511920e-02   5.6687920e-02   5.6591920e-02   5.6399920e-02
5.6351920e-02   5.6543920e-02   5.6543920e-02   5.6607920e-02   5.6623920e-02
5.6639920e-02   5.6415920e-02   5.6479920e-02   5.6415920e-02   5.6495920e-02
5.6367920e-02   5.6447920e-02   5.6495920e-02   5.6463920e-02   5.6639920e-02;
   5.9935920e-02   5.9727920e-02   5.9567920e-02   5.9439920e-02   5.9695920e-02
  5.9759920e-02   5.9471920e-02   5.9439920e-02   5.9535920e-02   5.9967920e-02
 5.9583920e-02   5.9631920e-02   5.9487920e-02   5.9455920e-02   5.9551920e-02
5.9743920e-02   5.9903920e-02   5.9919920e-02   5.9679920e-02   5.9279920e-02
5.9647920e-02   5.9695920e-02   5.9631920e-02   5.9855920e-02   5.9743920e-02
5.9567920e-02   5.9567920e-02   5.9807920e-02   5.9567920e-02   5.9471920e-02
5.9471920e-02   5.9663920e-02   5.9615920e-02   5.9583920e-02   5.9583920e-02
5.9343920e-02   5.9711920e-02   5.9583920e-02   5.9759920e-02   5.9471920e-02
5.9471920e-02   5.9711920e-02   5.9791920e-02   5.9407920e-02   5.9791920e-02
5.9711920e-02   5.9599920e-02   5.9231920e-02   5.9455920e-02   5.9663920e-02;
   6.2943920e-02   6.2943920e-02   6.2943920e-02   6.2783920e-02   6.3071920e-02
  6.2927920e-02   6.2783920e-02   6.2911920e-02   6.2703920e-02   6.2623920e-02
 6.3007920e-02   6.3039920e-02   6.3023920e-02   6.2703920e-02   6.3135920e-02
6.2991920e-02   6.2959920e-02   6.2863920e-02   6.2799920e-02   6.2703920e-02
6.2815920e-02   6.2975920e-02   6.2623920e-02   6.2847920e-02   6.3167920e-02
6.2559920e-02   6.3071920e-02   6.3039920e-02   6.2911920e-02   6.2767920e-02
6.3071920e-02   6.2847920e-02   6.2799920e-02   6.2543920e-02   6.2975920e-02
6.2799920e-02   6.3103920e-02   6.2943920e-02   6.2927920e-02   6.3023920e-02
6.2911920e-02   6.2703920e-02   6.2751920e-02   6.2975920e-02   6.2719920e-02
6.2799920e-02   6.3231920e-02   6.2799920e-02   6.2911920e-02   6.2751920e-02];
t_u_max_2 = [3.8495920e-02   3.8719920e-02   3.8655920e-02   3.8879920e-02
3.8831920e-02   3.8559920e-02   3.8463920e-02   3.8783920e-02   3.8607920e-02
3.8495920e-02   3.8719920e-02   3.8527920e-02   3.8703920e-02   3.8399920e-02
3.8975920e-02   3.8543920e-02   3.8703920e-02   3.8527920e-02   3.8911920e-02
3.9055920e-02   3.8623920e-02   3.8335920e-02   3.8463920e-02   3.8431920e-02
3.8495920e-02   3.8575920e-02   3.8767920e-02   3.8463920e-02   3.8495920e-02
3.8831920e-02   3.8719920e-02   3.8943920e-02   3.8815920e-02   3.8879920e-02
3.8863920e-02   3.8703920e-02   3.8767920e-02   3.8735920e-02   3.9007920e-02
```

```
3.8895920e-02   3.8175920e-02   3.8447920e-02   3.8863920e-02   3.8639920e-02
3.8831920e-02   3.8719920e-02   3.8943920e-02   3.9055920e-02   3.9167920e-02
3.8751920e-02;
   4.0223920e-02   4.0015920e-02   4.0671920e-02   4.0511920e-02   4.0319920e-02
  4.0383920e-02   4.0287920e-02   4.0383920e-02   3.9999920e-02   4.0495920e-02
 4.0623920e-02   4.0383920e-02   4.0063920e-02   4.0399920e-02   3.9887920e-02
4.0447920e-02   4.0351920e-02   4.0495920e-02   4.0351920e-02   4.0239920e-02
4.0383920e-02   4.0799920e-02   4.0255920e-02   4.0351920e-02   4.0367920e-02
4.0383920e-02   4.0143920e-02   4.0447920e-02   4.0271920e-02   4.0319920e-02
4.0399920e-02   4.0463920e-02   4.0223920e-02   4.0303920e-02   4.0591920e-02
4.0319920e-02   4.0191920e-02   4.0367920e-02   4.0367920e-02   4.0447920e-02
4.0447920e-02   4.0431920e-02   4.0223920e-02   4.0271920e-02   4.0495920e-02
4.0255920e-02   4.0255920e-02   4.0271920e-02   4.0431920e-02   4.0335920e-02;
   4.2607920e-02   4.2719920e-02   4.2479920e-02   4.2575920e-02   4.2479920e-02
  4.2415920e-02   4.2639920e-02   4.2479920e-02   4.2783920e-02   4.2559920e-02
 4.2335920e-02   4.2751920e-02   4.2479920e-02   4.2383920e-02   4.2559920e-02
4.2623920e-02   4.2191920e-02   4.2559920e-02   4.2415920e-02   4.2319920e-02
4.2351920e-02   4.2879920e-02   4.2447920e-02   4.2591920e-02   4.2415920e-02
4.2463920e-02   4.2255920e-02   4.2591920e-02   4.2319920e-02   4.2319920e-02
4.2591920e-02   4.2655920e-02   4.2479920e-02   4.2559920e-02   4.2415920e-02
4.2351920e-02   4.2415920e-02   4.2319920e-02   4.2319920e-02   4.2527920e-02
4.2191920e-02   4.2383920e-02   4.2543920e-02   4.2303920e-02   4.2591920e-02
4.2447920e-02   4.2543920e-02   4.2399920e-02   4.2639920e-02   4.2815920e-02;
   4.5039920e-02   4.5119920e-02   4.5103920e-02   4.5311920e-02   4.5055920e-02
  4.4943920e-02   4.5071920e-02   4.5503920e-02   4.5471920e-02   4.5423920e-02
 4.5135920e-02   4.5423920e-02   4.5231920e-02   4.4991920e-02   4.5263920e-02
4.5183920e-02   4.5327920e-02   4.4991920e-02   4.5199920e-02   4.5167920e-02
4.5167920e-02   4.5231920e-02   4.5247920e-02   4.5263920e-02   4.5519920e-02
4.5311920e-02   4.5279920e-02   4.5103920e-02   4.5007920e-02   4.5247920e-02
4.5343920e-02   4.5215920e-02   4.5407920e-02   4.5327920e-02   4.5103920e-02
4.5039920e-02   4.5247920e-02   4.5199920e-02   4.5359920e-02   4.5279920e-02
4.5375920e-02   4.5119920e-02   4.5215920e-02   4.5103920e-02   4.5199920e-02
4.5103920e-02   4.5151920e-02   4.5231920e-02   4.5151920e-02   4.5375920e-02;
   4.8671920e-02   4.8511920e-02   4.8319920e-02   4.8207920e-02   4.8463920e-02
  4.8527920e-02   4.8239920e-02   4.8223920e-02   4.8303920e-02   4.8703920e-02
 4.8351920e-02   4.8415920e-02   4.8239920e-02   4.8255920e-02   4.8319920e-02
4.8511920e-02   4.8671920e-02   4.8687920e-02   4.8447920e-02   4.7967920e-02
4.8415920e-02   4.8447920e-02   4.8431920e-02   4.8623920e-02   4.8527920e-02
4.8367920e-02   4.8335920e-02   4.8511920e-02   4.8335920e-02   4.8255920e-02
4.8223920e-02   4.8415920e-02   4.8319920e-02   4.8351920e-02   4.8319920e-02
4.8063920e-02   4.8447920e-02   4.8351920e-02   4.8527920e-02   4.8255920e-02
4.8255920e-02   4.8495920e-02   4.8543920e-02   4.8223920e-02   4.8559920e-02
4.8511920e-02   4.8319920e-02   4.7983920e-02   4.8207920e-02   4.8447920e-02;
   5.1631920e-02   5.1679920e-02   5.1615920e-02   5.1439920e-02   5.1743920e-02
  5.1599920e-02   5.1471920e-02   5.1567920e-02   5.1327920e-02   5.1279920e-02
 5.1663920e-02   5.1695920e-02   5.1663920e-02   5.1359920e-02   5.1807920e-02
5.1679920e-02   5.1631920e-02   5.1535920e-02   5.1423920e-02   5.1375920e-02
5.1471920e-02   5.1631920e-02   5.1327920e-02   5.1503920e-02   5.1871920e-02
5.1199920e-02   5.1775920e-02   5.1711920e-02   5.1583920e-02   5.1455920e-02
5.1743920e-02   5.1519920e-02   5.1487920e-02   5.1135920e-02   5.1695920e-02
5.1471920e-02   5.1791920e-02   5.1647920e-02   5.1599920e-02   5.1663920e-02
5.1567920e-02   5.1375920e-02   5.1391920e-02   5.1663920e-02   5.1407920e-02
5.1439920e-02   5.1855920e-02   5.1439920e-02   5.1583920e-02   5.1455920e-02];
l_max_2 =   [8.5085824e-02   8.5039247e-02   8.5097599e-02   8.5084113e-02
```

8.5041355e-02   8.5066005e-02   8.5030865e-02   8.5006725e-02   8.5061518e-02
8.5070011e-02   8.5086791e-02   8.5026940e-02   8.5085679e-02   8.5078754e-02
8.5073110e-02   8.5071514e-02   8.5048895e-02   8.5040790e-02   8.5051330e-02
8.5035368e-02   8.5009245e-02   8.5055083e-02   8.5023722e-02   8.5017810e-02
8.5048663e-02   8.5068933e-02   8.5073537e-02   8.5095318e-02   8.5057644e-02
8.5033260e-02   8.5048910e-02   8.5026405e-02   8.5085862e-02   8.5042637e-02
8.5069802e-02   8.5119373e-02   8.5015125e-02   8.5085323e-02   8.5088896e-02
8.5053293e-02   8.5092273e-02   8.5023396e-02   8.5051329e-02   8.5017334e-02
8.5025040e-02   8.5026652e-02   8.5004558e-02   8.5047025e-02   8.5035293e-02
8.5048517e-02;
8.5207805e-02   8.5203161e-02   8.5211101e-02   8.5212124e-02   8.5237512e-02
8.5187771e-02   8.5239737e-02   8.5243525e-02   8.5224813e-02   8.5246287e-02
8.5220067e-02   8.5227323e-02   8.5225125e-02   8.5233327e-02   8.5216135e-02
8.5247577e-02   8.5211574e-02   8.5222928e-02   8.5231559e-02   8.5242294e-02
8.5206382e-02   8.5251580e-02   8.5238973e-02   8.5226970e-02   8.5237642e-02
8.5260672e-02   8.5223388e-02   8.5237926e-02   8.5216243e-02   8.5247324e-02
8.5228473e-02   8.5228197e-02   8.5250679e-02   8.5258772e-02   8.5188179e-02
8.5222282e-02   8.5187815e-02   8.5265961e-02   8.5181774e-02   8.5208790e-02
8.5255564e-02   8.5239510e-02   8.5236089e-02   8.5246887e-02   8.5197512e-02
8.5206861e-02   8.5232188e-02   8.5207450e-02   8.5209789e-02   8.5236827e-02;
8.5288140e-02   8.5322862e-02   8.5312431e-02   8.5305213e-02   8.5334182e-02
8.5321385e-02   8.5305853e-02   8.5276138e-02   8.5310816e-02   8.5312010e-02
8.5321682e-02   8.5333331e-02   8.5308176e-02   8.5276664e-02   8.5310600e-02
8.5325478e-02   8.5324550e-02   8.5302575e-02   8.5292429e-02   8.5319457e-02
8.5349642e-02   8.5296428e-02   8.5302476e-02   8.5322079e-02   8.5295977e-02
8.5327529e-02   8.5271260e-02   8.5340065e-02   8.5306191e-02   8.5294420e-02
8.5291521e-02   8.5296061e-02   8.5287395e-02   8.5331733e-02   8.5293683e-02
8.5322067e-02   8.5314159e-02   8.5309727e-02   8.5320884e-02   8.5303167e-02
8.5326180e-02   8.5296159e-02   8.5301291e-02   8.5309344e-02   8.5351760e-02
8.5311293e-02   8.5280646e-02   8.5328955e-02   8.5316413e-02   8.5332872e-02;
8.5220564e-02   8.5166109e-02   8.5245831e-02   8.5205569e-02   8.5230177e-02
8.5224857e-02   8.5204924e-02   8.5197766e-02   8.5189293e-02   8.5193106e-02
8.5215976e-02   8.5225010e-02   8.5166478e-02   8.5218536e-02   8.5221127e-02
8.5232297e-02   8.5204964e-02   8.5218911e-02   8.5230885e-02   8.5166282e-02
8.5251610e-02   8.5204652e-02   8.5224907e-02   8.5222756e-02   8.5214899e-02
8.5165970e-02   8.5231177e-02   8.5230984e-02   8.5236292e-02   8.5194511e-02
8.5213742e-02   8.5182999e-02   8.5183527e-02   8.5215587e-02   8.5209754e-02
8.5220067e-02   8.5208472e-02   8.5191425e-02   8.5192221e-02   8.5203772e-02
8.5173894e-02   8.5213062e-02   8.5241307e-02   8.5230199e-02   8.5198371e-02
8.5234258e-02   8.5207199e-02   8.5248611e-02   8.5211972e-02   8.5184420e-02;
8.4930845e-02   8.4995847e-02   8.4982080e-02   8.4951431e-02   8.5001481e-02
8.4974748e-02   8.4979080e-02   8.5013045e-02   8.5001271e-02   8.4951899e-02
8.4990181e-02   8.5005695e-02   8.4992505e-02   8.5026373e-02   8.4994075e-02
8.5000798e-02   8.5003746e-02   8.4962066e-02   8.5025221e-02   8.4924046e-02
8.4983052e-02   8.5003050e-02   8.5022708e-02   8.4984654e-02   8.4983254e-02
8.5002733e-02   8.4934227e-02   8.4939500e-02   8.4958204e-02   8.4988865e-02
8.4943583e-02   8.4958566e-02   8.4980005e-02   8.4991543e-02   8.4929358e-02
8.4993157e-02   8.4985227e-02   8.4991126e-02   8.5017637e-02   8.5008450e-02
8.4983090e-02   8.5009669e-02   8.4928639e-02   8.5013340e-02   8.5013800e-02
8.5010848e-02   8.4991042e-02   8.5006189e-02   8.5027235e-02   8.4977490e-02;
8.4754405e-02   8.4814996e-02   8.4746352e-02   8.4748661e-02   8.4731615e-02
8.4741909e-02   8.4802360e-02   8.4730526e-02   8.4735504e-02   8.4751419e-02
8.4786027e-02   8.4731592e-02   8.4733537e-02   8.4739809e-02   8.4757124e-02
8.4765603e-02   8.4729750e-02   8.4730352e-02   8.4719361e-02   8.4755121e-02

```
8.4756078e-02   8.4722068e-02   8.4794979e-02   8.4737371e-02   8.4754485e-02
8.4739561e-02   8.4799237e-02   8.4745635e-02   8.4721425e-02   8.4767035e-02
8.4762489e-02   8.4757953e-02   8.4742868e-02   8.4710281e-02   8.4790609e-02
8.4777463e-02   8.4726265e-02   8.4786345e-02   8.4756491e-02   8.4747529e-02
8.4733553e-02   8.4772754e-02   8.4749719e-02   8.4776023e-02   8.4749006e-02
8.4741428e-02   8.4692710e-02   8.4740089e-02   8.4739724e-02   8.4792769e-02];
u_max_2 =   [1.8379878e+00   1.8064753e+00   1.8561351e+00   1.8310118e+00
1.8109433e+00   1.8284691e+00   1.8214739e+00   1.7796548e+00   1.8179832e+00
1.8181680e+00   1.8343553e+00   1.8066287e+00   1.8316719e+00   1.8373120e+00
1.8235007e+00   1.8278032e+00   1.8209861e+00   1.8146220e+00   1.8050655e+00
1.7997872e+00   1.7922396e+00   1.8270670e+00   1.8099593e+00   1.7967146e+00
1.8184355e+00   1.8354129e+00   1.8249624e+00   1.8523165e+00   1.8180868e+00
1.8015212e+00   1.8190518e+00   1.8010692e+00   1.8311674e+00   1.8046887e+00
1.8236355e+00   1.8512796e+00   1.7928811e+00   1.8266170e+00   1.8329145e+00
1.8131544e+00   1.8543648e+00   1.8108598e+00   1.8101971e+00   1.8010294e+00
1.7931874e+00   1.7936659e+00   1.7794681e+00   1.8021083e+00   1.7897872e+00
1.8008833e+00;
   1.9767233e+00   1.9772423e+00   1.9742452e+00   1.9582895e+00   1.9790670e+00
  1.9429214e+00   1.9886682e+00   1.9923296e+00   1.9878564e+00   1.9875761e+00
 1.9617480e+00   1.9786795e+00   1.9827753e+00   1.9763614e+00   1.9823779e+00
1.9870144e+00   1.9838466e+00   1.9696858e+00   1.9805066e+00   1.9931933e+00
1.9680617e+00   1.9910992e+00   1.9938848e+00   1.9746539e+00   1.9870061e+00
1.9921031e+00   1.9822860e+00   1.9864447e+00   1.9603985e+00   2.0005513e+00
1.9756052e+00   1.9751322e+00   1.9980465e+00   2.0001679e+00   1.9504070e+00
1.9734129e+00   1.9569986e+00   1.9954353e+00   1.9552413e+00   1.9635298e+00
2.0001922e+00   1.9747799e+00   1.9884984e+00   1.9866319e+00   1.9619432e+00
1.9665245e+00   1.9892258e+00   1.9620545e+00   1.9703595e+00   2.0006264e+00;
   2.1123041e+00   2.1160942e+00   2.1037421e+00   2.1332169e+00   2.1182407e+00
  2.1224186e+00   2.1164978e+00   2.0760492e+00   2.0970660e+00   2.1151846e+00
 2.1206084e+00   2.1145806e+00   2.1125989e+00   2.0999048e+00   2.0978481e+00
2.1176356e+00   2.1212954e+00   2.0942997e+00   2.0910643e+00   2.1297444e+00
2.1309302e+00   2.0938645e+00   2.1223497e+00   2.1191734e+00   2.1104721e+00
2.1238174e+00   2.0953553e+00   2.1286058e+00   2.1137919e+00   2.1183481e+00
2.1193984e+00   2.1018510e+00   2.1039518e+00   2.1253484e+00   2.1057302e+00
2.1191661e+00   2.1192640e+00   2.1064195e+00   2.1137611e+00   2.1043571e+00
2.1268494e+00   2.1026030e+00   2.1016770e+00   2.1130010e+00   2.1316185e+00
2.1216867e+00   2.0950417e+00   2.1256623e+00   2.1112602e+00   2.1256876e+00;
   2.1522725e+00   2.1284685e+00   2.1609453e+00   2.1473790e+00   2.1601663e+00
  2.1698999e+00   2.1385891e+00   2.1532496e+00   2.1444956e+00   2.1478774e+00
 2.1575341e+00   2.1694962e+00   2.1465564e+00   2.1686168e+00   2.1575698e+00
2.1673958e+00   2.1528319e+00   2.1451318e+00   2.1500939e+00   2.1396587e+00
2.1772953e+00   2.1614361e+00   2.1685694e+00   2.1670035e+00   2.1527400e+00
2.1348197e+00   2.1759807e+00   2.1595486e+00   2.1603009e+00   2.1541075e+00
2.1548251e+00   2.1469182e+00   2.1409210e+00   2.1612727e+00   2.1496810e+00
2.1537283e+00   2.1497848e+00   2.1478640e+00   2.1534353e+00   2.1479947e+00
2.1440638e+00   2.1516565e+00   2.1750028e+00   2.1467996e+00   2.1427952e+00
2.1614639e+00   2.1481757e+00   2.1734807e+00   2.1526098e+00   2.1510068e+00;
   2.0753030e+00   2.1070706e+00   2.1055384e+00   2.0967121e+00   2.1096405e+00
  2.1036214e+00   2.1003483e+00   2.1142736e+00   2.1159326e+00   2.0939281e+00
 2.1070159e+00   2.1174418e+00   2.1034412e+00   2.1210141e+00   2.1121465e+00
2.1048911e+00   2.1100012e+00   2.0939367e+00   2.1107405e+00   2.0791661e+00
2.1030371e+00   2.1134573e+00   2.1234746e+00   2.1042470e+00   2.1040852e+00
2.1084977e+00   2.0844806e+00   2.0845764e+00   2.0969501e+00   2.1092403e+00
2.0951343e+00   2.0903742e+00   2.1038675e+00   2.1082038e+00   2.0850156e+00
```

```
2.1083769e+00   2.1050159e+00   2.1076724e+00   2.1223795e+00   2.1220676e+00
2.1054315e+00   2.1139508e+00   2.0824090e+00   2.1204509e+00   2.1130949e+00
2.1128957e+00   2.1000822e+00   2.1102079e+00   2.1138289e+00   2.1015652e+00;
   2.0104224e+00   2.0397473e+00   2.0040903e+00   2.0085403e+00   2.0012516e+00
  2.0058769e+00   2.0334559e+00   1.9973331e+00   2.0027931e+00   2.0108352e+00
 2.0209017e+00   2.0015668e+00   1.9996270e+00   2.0052499e+00   2.0123602e+00
2.0129368e+00   2.0010519e+00   2.0026398e+00   1.9889444e+00   2.0089269e+00
2.0119409e+00   1.9945778e+00   2.0285649e+00   2.0050975e+00   2.0064745e+00
2.0046544e+00   2.0321502e+00   2.0070759e+00   1.9933457e+00   2.0183104e+00
2.0125614e+00   2.0113540e+00   2.0035812e+00   1.9932353e+00   2.0239997e+00
2.0212430e+00   1.9956156e+00   2.0261797e+00   2.0125376e+00   2.0071737e+00
2.0011775e+00   2.0208328e+00   2.0088496e+00   2.0189635e+00   2.0077043e+00
2.0023067e+00   1.9809520e+00   2.0022591e+00   2.0026025e+00   2.0278371e+00];
```

```
 delay_1  delay_2   mu1_mean mu1_median   mu1_mode mu1_HDIlow mu1_HDIhigh
mu1_Vdet mu1_pcgtVdet mu1_ROPElow mu1_ROPEhigh mu1_pcInROPE
     -10       -5     1.8161     1.8161     1.8162     1.8123      1.8197
1.8440         0.00       1.8340       1.8540         0.00
     -10        0     1.8161     1.8161     1.8161     1.8123      1.8197
1.8440         0.00       1.8340       1.8540         0.00
     -10        5     1.8161     1.8161     1.8162     1.8125      1.8198
1.8440         0.00       1.8340       1.8540         0.00
     -10       10     1.8161     1.8161     1.8161     1.8125      1.8199
1.8440         0.00       1.8340       1.8540         0.00
     -10       15     1.8161     1.8161     1.8162     1.8124      1.8198
1.8440         0.00       1.8340       1.8540         0.00
      -5        0     1.9766     1.9766     1.9766     1.9735      1.9794
2.0620         0.00       2.0520       2.0720         0.00
      -5        5     1.9765     1.9765     1.9765     1.9735      1.9794
2.0620         0.00       2.0520       2.0720         0.00
      -5       10     1.9765     1.9765     1.9766     1.9735      1.9794
2.0620         0.00       2.0520       2.0720         0.00
      -5       15     1.9765     1.9765     1.9765     1.9735      1.9794
2.0620         0.00       2.0520       2.0720         0.00
       0        5     2.1129     2.1129     2.1128     2.1104      2.1153
2.2540         0.00       2.2440       2.2640         0.00
       0       10     2.1129     2.1129     2.1129     2.1105      2.1153
2.2540         0.00       2.2440       2.2640         0.00
       0       15     2.1129     2.1129     2.1129     2.1104      2.1153
2.2540         0.00       2.2440       2.2640         0.00
       5       10     2.1544     2.1544     2.1545     2.1522      2.1564
2.2920         0.00       2.2820       2.3020         0.00
       5       15     2.1544     2.1544     2.1543     2.1522      2.1565
2.2920         0.00       2.2820       2.3020         0.00
      10       15     2.1062     2.1062     2.1062     2.1039      2.1085
2.1730         0.00       2.1630       2.1830         0.00
```

```
 delay_1  delay_2   mu2_mean mu2_median   mu2_mode mu2_HDIlow mu2_HDIhigh
mu2_Vdet mu2_pcgtVdet mu2_ROPElow mu2_ROPEhigh mu2_pcInROPE
     -10       -5     1.9765     1.9765     1.9765     1.9735      1.9795
2.0620         0.00       2.0520       2.0720         0.00
     -10        0     2.1129     2.1129     2.1128     2.1104      2.1153
2.2540         0.00       2.2440       2.2640         0.00
     -10        5     2.1544     2.1544     2.1544     2.1522      2.1565
```

```
2.2920          0.00      2.2820       2.3020         0.00
     -10        10      2.1062      2.1062      2.1062     2.1039      2.1085
2.1730          0.00      2.1630       2.1830         0.00
     -10        15      2.0108      2.0108      2.0108     2.0083      2.0131
2.0460          0.00      2.0360       2.0560         0.00
      -5         0      2.1129      2.1129      2.1129     2.1104      2.1153
2.2540          0.00      2.2440       2.2640         0.00
      -5         5      2.1544      2.1544      2.1544     2.1522      2.1565
2.2920          0.00      2.2820       2.3020         0.00
      -5        10      2.1062      2.1062      2.1062     2.1039      2.1085
2.1730          0.00      2.1630       2.1830         0.00
      -5        15      2.0108      2.0108      2.0108     2.0084      2.0131
2.0460          0.00      2.0360       2.0560         0.00
       0         5      2.1544      2.1544      2.1544     2.1522      2.1565
2.2920          0.00      2.2820       2.3020         0.00
       0        10      2.1062      2.1063      2.1063     2.1040      2.1085
2.1730          0.00      2.1630       2.1830         0.00
       0        15      2.0108      2.0108      2.0107     2.0084      2.0132
2.0460          0.00      2.0360       2.0560         0.00
       5        10      2.1062      2.1062      2.1062     2.1039      2.1085
2.1730          0.00      2.1630       2.1830         0.00
       5        15      2.0108      2.0108      2.0107     2.0084      2.0131
2.0460          0.00      2.0360       2.0560         0.00
      10        15      2.0108      2.0108      2.0107     2.0083      2.0131
2.0460          0.00      2.0360       2.0560         0.00

 delay_1  delay_2 muDiff_mean muDiff_median muDiff_mode muDiff_HDIlow
muDiff_HDIhigh muDiff_pcgtZero muDiff_ROPElow muDiff_ROPEhigh   pcInROPE
     -10        -5     -0.1604       -0.1604     -0.1604       -0.1652
-0.1557            0.00        -0.0100          0.0100       0.00
     -10         0     -0.2968       -0.2968     -0.2966       -0.3012
-0.2924            0.00        -0.0100          0.0100       0.00
     -10         5     -0.3383       -0.3382     -0.3382       -0.3425
-0.3339            0.00        -0.0100          0.0100       0.00
     -10        10     -0.2901       -0.2901     -0.2899       -0.2944
-0.2857            0.00        -0.0100          0.0100       0.00
     -10        15     -0.1946       -0.1946     -0.1947       -0.1990
-0.1902            0.00        -0.0100          0.0100       0.00
      -5         0     -0.1363       -0.1363     -0.1365       -0.1401
-0.1325            0.00        -0.0100          0.0100       0.00
      -5         5     -0.1779       -0.1779     -0.1781       -0.1815
-0.1741            0.00        -0.0100          0.0100       0.00
      -5        10     -0.1297       -0.1297     -0.1297       -0.1334
-0.1260            0.00        -0.0100          0.0100       0.00
      -5        15     -0.0342       -0.0342     -0.0342       -0.0381
-0.0305            0.00        -0.0100          0.0100       0.00
       0         5     -0.0415       -0.0415     -0.0415       -0.0448
-0.0383            0.00        -0.0100          0.0100       0.00
       0        10      0.0067        0.0067      0.0066        0.0033
0.0099          100.00        -0.0100          0.0100      97.54
       0        15      0.1021        0.1021      0.1022        0.0987
0.1055          100.00        -0.0100          0.0100       0.00
       5        10      0.0482        0.0482      0.0480        0.0451
0.0513          100.00        -0.0100          0.0100       0.00
```

5	15	0.1436	0.1436	0.1437	0.1404	0.1468	100.00	-0.0100	0.0100	0.00
10	15	0.0955	0.0954	0.0953	0.0921	0.0988	100.00	-0.0100	0.0100	0.00

delay_1	delay_2	nu_mean	nu_median	nu_mode	nu_HDIlow	nu_HDIhigh	nuLog10_mean	nuLog10_median	nuLog10_mode	nuLog10_HDIlow	nuLog10_HDIhigh
-10	-5	51.5870	43.0669	27.3520	7.1387	118.6650	1.6283	1.6341	1.6465	1.0922	2.1504
-10	0	42.0855	33.3656	17.3111	5.1451	104.0650	1.5217	1.5233	1.5420	0.9467	2.0950
-10	5	53.5489	45.0495	27.8382	7.6278	121.5460	1.6474	1.6537	1.6558	1.1237	2.1666
-10	10	47.3602	38.7951	24.9654	5.3509	112.2470	1.5817	1.5888	1.5886	1.0116	2.1264
-10	15	49.7387	41.2955	26.4245	6.9837	115.8750	1.6087	1.6159	1.6415	1.0523	2.1331
-5	0	42.3189	33.6209	20.4978	5.4854	104.2860	1.5266	1.5266	1.5351	0.9588	2.0934
-5	5	53.8168	45.4545	30.2174	7.7677	121.4360	1.6507	1.6576	1.6868	1.1326	2.1647
-5	10	47.8656	39.2891	22.7682	5.9903	113.0760	1.5878	1.5943	1.5998	1.0330	2.1377
-5	15	49.9608	41.4719	23.9667	6.6619	115.9730	1.6117	1.6178	1.6135	1.0646	2.1372
0	5	44.8759	36.2716	20.3257	5.6506	107.9010	1.5558	1.5596	1.5769	0.9935	2.1118
0	10	37.3335	28.6718	16.5778	4.2045	95.8793	1.4581	1.4575	1.4849	0.8585	2.0588
0	15	40.1013	31.5433	18.1529	4.8277	100.2390	1.4974	1.4989	1.5258	0.9086	2.0721
5	10	49.9917	41.4347	26.0066	6.6139	116.7890	1.6101	1.6174	1.6447	1.0677	2.1551
5	15	52.3180	43.8067	27.2308	7.2237	120.0880	1.6345	1.6415	1.6659	1.0996	2.1605
10	15	45.5514	37.0457	19.7746	5.0464	109.4680	1.5610	1.5687	1.5976	0.9847	2.1221

delay_1	delay_2	effSz_mean	effSz_median	effSz_mode	effSz_HDIlow	effSz_HDIhigh	effSz_pcgtZero	effSz_ROPElow	effSz_ROPEhigh	effSz_pcInROPE
-10	-5	-9.6807	-9.6709	-9.6501	-10.7903	-8.5872	0.0000	-0.1000	0.1000	0.0000
-10	0	-19.3635	-19.3254	-19.2844	-21.7698	-17.0217	0.0000	-0.1000	0.1000	0.0000
-10	5	-22.6732	-22.6569	-22.7073	-25.4197	-20.0236	0.0000	-0.1000	0.1000	0.0000
-10	10	-19.1525	-19.1272	-19.0940	-21.4992	-16.8868	0.0000	-0.1000	0.1000	0.0000
-10	15	-12.6586	-12.6415	-12.5690	-14.1528	-11.1457	0.0000	-0.1000	0.1000	0.0000
-5	0	-10.2775	-10.2559	-10.2461	-11.5090	-9.0593	0.0000	-0.1000	0.1000	0.0000
-5	5	-13.9439	-13.9339	-13.8952	-15.5280	-12.3811	0.0000	-0.1000	0.1000	0.0000

```
   -5       10    -9.9462      -9.9300    -9.9113     -11.1323        -8.8211
    0.0000       -0.1000         0.1000        0.0000
   -5       15    -2.5754      -2.5739    -2.5470      -2.9759        -2.1767
    0.0000       -0.1000         0.1000        0.0000
    0        5    -3.7092      -3.7055    -3.6823      -4.2086        -3.2124
    0.0000       -0.1000         0.1000        0.0000
    0       10     0.5837       0.5830     0.5882       0.2858         0.8859
   99.9955       -0.1000         0.1000        0.0705
    0       15     8.6868       8.6623     8.6348       7.6378         9.8002
  100.0000       -0.1000         0.1000        0.0000
    5       10     4.4233       4.4182     4.3921       3.8714         4.9779
  100.0000       -0.1000         0.1000        0.0000
    5       15    12.8378      12.8201    12.7694      11.4303        14.2503
  100.0000       -0.1000         0.1000        0.0000
   10       15     8.2984       8.2798     8.2605       7.3152         9.2914
  100.0000       -0.1000         0.1000        0.0000

 delay_1  delay_2 sigma1_mean sigma1_median sigma1_mode sigma1_HDIlow
sigma1_HDIhigh sigma2_mean sigma2_median  sigma2_mode sigma2_HDIlow
sigma2_HDIhigh sigmaDiff_mean sigmaDiff_median sigmaDiff_mode sigmaDiff_HDIlow
sigmaDiff_HDIhigh sigmaDiff_pcgtZero
     -10       -5    0.018324      0.018253    0.018184      0.015691
0.021142    0.014674      0.014616     0.014484      0.012557       0.016905
  0.003649         0.003627       0.003545         0.000185         0.007105
          98.22
     -10        0    0.018209      0.018143    0.018031      0.015508
0.021064    0.011861      0.011824     0.011694      0.010048       0.013796
  0.006348         0.006306       0.006225         0.003169         0.009635
         100.00
     -10        5    0.018348      0.018273    0.018127      0.015703
0.021127    0.010528      0.010486     0.010408      0.009029       0.012125
  0.007820         0.007777       0.007681         0.004790         0.010980
         100.00
     -10       10    0.018278      0.018210    0.018175      0.015602
0.021086    0.011275      0.011234     0.011123      0.009601       0.013055
  0.007003         0.006959       0.006759         0.003873         0.010218
         100.00
     -10       15    0.018309      0.018235    0.018168      0.015624
0.021097    0.011829      0.011783     0.011714      0.010068       0.013613
  0.006480         0.006435       0.006241         0.003354         0.009758
         100.00
      -5        0    0.014583      0.014532    0.014478      0.012404
0.016815    0.011865      0.011825     0.011726      0.010044       0.013770
  0.002718         0.002708       0.002821        -0.000088         0.005516
          97.36
      -5        5    0.014692      0.014635    0.014515      0.012577
0.016906    0.010528      0.010481     0.010370      0.009011       0.012097
  0.004165         0.004141       0.004056         0.001560         0.006862
          99.91
      -5       10    0.014638      0.014584    0.014418      0.012459
0.016823    0.011273      0.011231     0.011138      0.009592       0.013032
  0.003365         0.003344       0.003355         0.000664         0.006117
          99.31
      -5       15    0.014657      0.014598    0.014587      0.012549
```

0.016896 0.011834 0.011788 0.011809 0.010090 0.013645
0.002823 0.002806 0.002806 0.000072 0.005614
97.89
0 5 0.011892 0.011849 0.011741 0.010098
0.013818 0.010476 0.010435 0.010377 0.008952 0.012090
0.001415 0.001406 0.001452 -0.000900 0.003846
88.21
0 10 0.011768 0.011737 0.011745 0.009886
0.013713 0.011149 0.011115 0.011049 0.009379 0.012946
0.000619 0.000615 0.000641 -0.001835 0.003044
69.29
0 15 0.011828 0.011791 0.011622 0.009952
0.013727 0.011740 0.011698 0.011555 0.009953 0.013601
0.000088 0.000086 -0.000002 -0.002407 0.002590
52.76
5 10 0.010503 0.010463 0.010403 0.008982
0.012086 0.011294 0.011252 0.011132 0.009631 0.013033
-0.000791 -0.000785 -0.000713 -0.003064 0.001462
24.48
5 15 0.010521 0.010479 0.010395 0.009013
0.012117 0.011851 0.011803 0.011740 0.010110 0.013643
-0.001330 -0.001320 -0.001376 -0.003670 0.000999
12.83
10 15 0.011252 0.011215 0.011154 0.009539
0.013001 0.011790 0.011747 0.011659 0.010050 0.013646
-0.000538 -0.000538 -0.000556 -0.002954 0.001907
32.88
