# Supplementary material for: Predicting the effects of spatiotemporal modifications of muscle activation on the tentacle extension in squid
Source: Front Bioeng Biotechnol. 2023 Oct 19;11:1193409. doi: 10.3389/fbioe.2023.1193409 (PMC10620692; doi:10.3389/fbioe.2023.1193409)
Supplement: Supplementary file 2 [file DataSheet1.pdf]

Supplementary Material

Predicting the Effects of Spatiotemporal Modifications of Muscle Activation on the Tentacle Extension in Squid

Johan L. van Leeuwen[1,*] **& William M. Kier**[2]

[1]*Experimental Zoology Group, Wageningen University, De Elst 1, 6708 WD Wageningen, The Netherlands,*
[2]*Department of Biology, CB#3280 Coker Hall, University of North Carolina, Chapel Hill, NC 27599-3280, U.S.A.*
[*]Correspondence: johan.vanleeuwen@wur.nl

1 SYMBOLS, DEFINITIONS AND ASSIGNED PARAMETER VALUES

Table S1 provides an overview of the symbols and definitions used in the main text. Where appropriate, assigned input values used in the simulations are provided.

Table S1: **Symbols, definitions, and assigned values**

**Symbol**	**Definition**
a_i	acceleration in longitudinal direction of boundary b_i (equation (S24)); ($a_i = \ddot{\ell}_i$)
a, b	parameters of the beta probability distribution; $a = b = 5.8$
A_{c0}	cross-sectional area of stalk in initial state; $A_{c0} = \pi r_0^2$
$A_{c,i}$	cross-sectional area of disc i; $A_{c,i} = \pi r_i^2$
$A_{c\ell 0}$	cross-sectional area of longitudinal muscles in initial state; $A_{c\ell 0} = 0.15 A_{c0}$
$A_{s0,i}$	lateral surface area of disc i in initial state; $A_{s0,i} = 2\pi r_0 h_{0,i}$
$A_{s,i}$	lateral surface area of disc i
b_i	boundary between disc i and disc $i+1$
c_{1pas}	constant in equation (S15); $c_{1pas} = 887.4\,\text{kPa}$
c_{2pas}	constant in equation (S15); $c_{2pas} = 2.260$
c_{3pas}	constant in equation (S16); $c_{3pas} = 1450.0\,\text{kPa}$
c_{4pas}	constant in equation (S16); $c_{4pas} = -625.0\,\text{kPa}$
C_{myo}	resistive constant for myosin filament (equation (S11); $C_{myo} = 0.44$
$c_{x,i}$	component in equation (S25); $x \in [1, 2, 3, 4]$; $i \in [1, 2, \cdots, n-2, n-1]$
D_{act}	constant to account for cross-bridge losses due to actin filament overlap in one sarcomere (equations (S10) and (S11); $D_{act} = 0.68$
D_{myo}	constant to account for cross-bridge losses due to interaction between myosin filament and Z-disc (equation (S11); $D_{myo} = 1.90$
E_{size}	effect size of a two-group statistical comparison; $E_{size} = (\mu_1 - \mu_2) \,/\sqrt{\left(\sigma_1^2 - \sigma_2^2\right)/2}$
F_i	net force on $m_{b,i}$ by segment i (equation (S22))
$f_{a,i}$	active state of muscle fibres in disc i (equations (S2–S4))
$f_{\ell,i}$	filamentary overlap function of disc i (equations (S8–S11))
$f_{v,i}$	velocity dependence function of disc i (equations (S6) and (S7))

Table S1: **Symbols, definitions, and assigned values** (continued)

Symbol	Definition
$g_{x,i}$	expression shown in the Section 2; $x \in [1, 2, 3, 4]$; $i \in [1, 2, \cdots, n-2, n-1]$
$h_{0,i}$	initial length (height) of tentacular disc i; $h_{0,i} = \ell_{s0}/(n-1)$ for $i \in [1, 2, ..., n-2, n-1]$; $h_{0,n} = m_{club}/\left(\rho\pi r_0^2\right)$
h_i	length (height) of tentacular disc i
$\dot{h}_i, \ddot{h}_i$	first and second time derivatives of h_i
k	constant in equations (S6) and (S7); $k = 0.25$
$\ell_{0sarc,i}$	optimum sarcomere length for active force production in segment i
$\ell_{0sarc,ref}$	idem, but for reference sarcomere; $\ell_{0sarc,ref} = 2.37\,\mu$m
$\ell_{act,i}$	length of two opposite thin filaments in one sarcomere of segment i (equations (S8–S11); $\ell_{act} = 1.3\ell_{myo}$
ℓ_i	position in longitudinal direction of boundary b_i
$\dot{\ell}_i, \ddot{\ell}_i$	velocity and acceleration in longitudinal direction of boundary b_i
ℓ_{bz}	length of bare zone on myosin filament (equations (S8–S11); $\ell_{bz} = 0.14\,\mu$m
$\ell_{myo,i}$	length of myosin filament in segment i (equations (S5) and (S8–S11); $\ell_{myo,i} = 0.8\,\mu$m
$\ell_{myo,ref}$	length of myosin filament of reference sarcomere (equation (S5); $\ell_{myo,ref} = 1.58\,\mu$m
ℓ_s	length of tentacular stalk
ℓ_{s0}	initial length of tentacular stalk; $\ell_{s0} = 53.143$ mm
$\ell_{sarc,i}$	sarcomere length in segment i
ℓ_z	width of Z-disc in sarcomeres; $\ell_z = 0.06\,\mu$m
$m_{b,i}$	mass associated with boundary b_i, $m_{b,i} = (m_i + m_{i+1})/2$ for $i \in [1, 2, ..., n-1, n-2]$; $m_{b,n-1} = m_{b,n-1}/2 + (1+\alpha)m_{club}$
m_{club}	mass of tentacular club; $m_{club} = 1.8$ gram
m_i	mass of tentacle segment i; $m_i = \pi r_0^2 h_{0,i}\rho$
n	number of segments of tentacle model; $n = 51$ (50 stalk segments, 1 club segment); n can also refer to the number of data in a group
p_i	pressure in segment i
p_t	t probability distribution (equation (2) in the main text)
q	exponent in activation equation (S3); $q = 15$
r	co-ordinate in radial direction
r_i	radius of tentacular disc i
$\dot{r}_i, \ddot{r}_i$	first and second time derivatives of r_i (equations (S19) and (S20))
r_0	initial radius of tentacular stalk; $r_0 = 3.7$ mm
t	time; t may also refer to the t distribution
t_a	time between start of activation and first moment of full activation (equations (S3) and (S4)); $t_a = 0.04$ s
$t_{d,i}$	delay of activation of segment i with respect to segment 1 (equations (S2–S4))
v_{max}	maximum extension velocity of the tentacular stalk
V_{max}	maximum unloaded shortening velocity of a muscle fiber (expressed in reference lengths per second)
x	variable in t distribution (equation (2) in the main text)
α	constant for defining the added mass of water; $\alpha = 0.08$

Table S1: **Symbols, definitions, and assigned values** (continued)

Symbol	Definition
ε_c	critical strain in equations (S17) and (S18); $\varepsilon_c = 0.773$
$\varepsilon_{\ell,i}$	strain in longitudinal direction of disc i
$\varepsilon_{r,i}$	strain in radial direction of disc i
$\dot{\varepsilon}_{min,i}$	minimum unloaded strain rate of disc i
$\dot{\varepsilon}_{min,ref}$	minimum unloaded strain rate of reference sarcomere; $\dot{\varepsilon}_{min,ref} = -17\,\mathrm{s}^{-1}$, which is equivalent to $V_{max} = 17\,\ell\,\mathrm{s}^{-1}$
η_i	volume fraction of extensor muscles in segment i. η is linearly interpolated between the proximal and distant segments of the tentacular stalk; $\eta_1 = 0.7$ for all simulations
Δt_d	deterministic activation delay between the tip and base segments of the tentacular stalk.
$\Delta t_{d,vmax}$	the Δt_d that results in the highest peak extension velocity of the tentacular stalk.
Γ	gamma function, used for t probability distribution
μ	viscous friction parameter associated with longitudinal strain rate (see equation (S22); $\mu = 38\,\mathrm{kg\,m^{-1}\,s^{-1}}$; μ can also refer to the mean value of a sample group (see equation (2) in the main text)
μ_1, μ_2	the means of, respectively, sample groups 1 and 2
ν	the shape parameter of the t probability distribution (equation (2) in the main text)
ρ	density of tentacular tissue; $\rho = 1050\,\mathrm{kg\,m^{-3}}$
σ	standard deviation
$\sigma_{\ell,i}$	average stress in longitudinal direction
$\sigma_{max,i}$	maximum isometric muscle stress in radial direction at $\ell_{0sarc,i}$, see equation (S5)
$\sigma_{max,ref}$	maximum isometric muscle stress of reference sarcomere at $\ell_{0sarc,ref}$; $\sigma_{max,ref} = 280\,\mathrm{kPa}$
$\sigma_{pas,i}$	passive component of muscle stress (equations (S1) and (S14–S15))
$\sigma_{r,i}$	muscle stress in radial direction (equation (S1))
ROPE	region of practical equivalence (ROPE $= [-0.01\ 0.01]$ for E_{size}; ROPE $= [v_{ref} - 0.01\ \ v_{ref} + 0.01]\,\mathrm{m\,s^{-1}}$ for velocity comparisons, where $v_{ref} = 0$ for a comparison of the mean of two data sets and $v_{ref} = v_{max}$ for a comparison of the mean)

2 SUMMARY OF THE MODEL

An overview of the definition of the symbols used for the model is provided in Supplementary Material: Table S1. Following van Leeuwen and Kier (1997), we assumed that the nominal tensile stress in the extensor muscles σ_r (i.e., the tensile force per cross-sectional area of the initial relaxed state; subscript r refers to the radial direction) depends on the maximum active isometric stress σ_{max} at optimum sarcomere length ℓ_{0sarc}, the normalized active state f_a, the velocity dependence function f_v, the filamentary overlap function f_ℓ, and a passive component σ_{pas}.

$$\sigma_{r,i} = f_{a,i}\sigma_{max,i}f_{v,i}f_{\ell,i} + \sigma_{pas,i}. \tag{S1}$$

The subscript i refers to the segmental number.

The activation of each muscular element was described with a function $f_{a,i}$ in the interval $[0, 1]$. The upper limit of the active state is described by $f_{a,i} = 1$, and $f_{a,i} = 0$ represents an inactive muscle. The

activation is assumed to rise from zero to the maximum level, and then to remain maximal. Delays in the onset of the activation among tentacular segments can be set in an arbitrary manner. The activation function was described by the following equations:

$$f_{a,i} = 0, \quad \text{for } t \leq t_{d,i}, \tag{S2}$$

$$f_{a,i} = \left(\frac{1}{2}\left(1+\sin\left(\frac{\pi(t-t_{d,i})}{t_a}-\frac{\pi}{2}\right)\right)\right)^q, \quad \text{for } t_{d,i} < t < t_{d,i}+t_a, \tag{S3}$$

$$f_{a,i} = 1, \quad \text{for } t \geq t_{d,i}+t_a, \tag{S4}$$

where t is time, $t_{d,i}$ is the activation delay with respect to the proximal segment, t_a is the time between the start and full activation, and parameter q allows us to modify the basic sinusoidal shape. The activation along the tentacle is an essential input of the model, and the effects of its variation on the extension performance are the focus of this paper.

Following van Leeuwen and Kier (1997), we assumed that

$$\sigma_{max,i} = \sigma_{max,ref}\left(\ell_{myo,i} - l_{bz}\right) / \left(\ell_{myo,ref} - \ell_{bz}\right), \tag{S5}$$

where $\ell_{myo,i}$ and ℓ_{bz} are the lengths of the myosin filament and the central bare zone on the myosin filament (which lacks myosin heads and cannot form cross-bridges). Furthermore, $\sigma_{max,ref} = 280$ kPa and $\ell_{myo,ref} = 1.58\,\mu$m represent values for a reference sarcomere. We assumed a fixed value of $0.14\,\mu$m for ℓ_{bz}.

The non-linear functions f_v, which depends on the longitudinal strain rate of the muscle fiber, and f_ℓ, which depends on the strain (and hence the overlap in of the myosin and actin filaments in the sarcomere), are key properties of the muscle material.

The dependence of the active force on the non-dimensional strain rate $\dot{\varepsilon}^*_{r,i}$ was described by:

$$f_{v,i} = 1.8 - 0.8(1+\dot{\varepsilon}^*_{r,i})/(1-7.56\dot{\varepsilon}^*_{r,i}/k), \quad \text{for } \dot{\varepsilon}^*_{r,i} < 0, \tag{S6}$$

$$f_{v,i} = (1-\dot{\varepsilon}^*_{r,i})/(1+\dot{\varepsilon}^*_{r,i}/k), \quad \text{for } \dot{\varepsilon}^*_{r,i} \geq 0, \tag{S7}$$

where k is a constant and $\dot{\varepsilon}^*_{r,i} = \dot{\varepsilon}_{r,i}/\dot{\varepsilon}_{min,i}$; $\dot{\varepsilon}_{r,i}$ is the strain rate and $\dot{\varepsilon}_{min,i}$ is the minimum (unloaded) strain rate. Equation (S6), formulated by Otten (1987), is based on stretch experiments of vertebrate muscles Aubert (1956). Equation (S7) represents the equation of Hill (1938).

The filamentary overlap function was defined after van Leeuwen (1991):

$$f_{\ell,i} = (\ell_{myo,i}+\ell_{act,i}+\ell_z-\ell_{sarc,i})/(\ell_{myo,i}-\ell_{bz}), \quad \text{for } \ell_{act,i}+\ell_{bz}+\ell_z \leq \ell_{sarc,i} \leq \ell_{myo,i}+\ell_{act,i}+\ell_z, \tag{S8}$$

$$f_{\ell,i} = 1, \quad \text{for } \ell_{act,i}+\ell_z \leq \ell_{sarc,i} \leq \ell_{act,i}+\ell_{bz}+\ell_z, \tag{S9}$$

$$f_{\ell,i} = (\ell_{myo,i}-\ell_{bz}-D_{act}(\ell_{act,i}+\ell_z-\ell_{sarc,i}))/(\ell_{myo,i}-\ell_{bz}), \quad \text{for } \ell_{myo,i}+\ell_z \leq \ell_{sarc} \leq \ell_{act,i}+\ell_z, \tag{S10}$$

$$f_{\ell,i} = (\ell_{myo,i}-\ell_{bz}-D_{act}(\ell_{act,i}+\ell_z-\ell_{sarc,i})-D_{myo}(\ell_{myo,i}+\ell_z-\ell_{sarc,i}) - C_{myo}(\ell_{myo,i}+\ell_z-\ell_{sarc,i}))/(\ell_{myo,i}-\ell_{bz}), \quad \text{for } \ell_{min,i} \leq \ell_{sarc,i} \leq \ell_{myo,i}+\ell_z, \tag{S11}$$

where, $\ell_{act,i}$ is the summed length of two opposing actin filaments in one sarcomere, ℓ_z is the width of the Z-disc, $\ell_{sarc,i}$ is the sarcomere length, and D_{act} and D_{myo} account for cross-bridge losses due to actin overlap and interaction between myosin filament and Z-disc. Finally, C_{myo} accounts for resistive forces owing to the collision of the myosin filaments with the Z-disc of the sarcomere. The reference sarcomere length for active force production is defined as:

$$\ell_{0sarc,i} = \ell_{act,i} + \ell_z + \ell_{bz}/2. \tag{S12}$$

All sarcomeres were prescribed to be at ℓ_{0sarc} in the initial relaxed state of the tentacle and in each segment all sarcomeres were assumed to have the same length. Hence, $\ell_{sarc,i}$ was calculated from:

$$\ell_{sarc,i} = \ell_{0sarc,i} + \varepsilon_{r,i}\ell_{0sarc,i}. \tag{S13}$$

The passive component in equation (S1) was expressed as (van Leeuwen and Kier, 1997):

$$\sigma_{pas,i} = 0, \quad \text{for } \varepsilon_i \leq 0, \tag{S14}$$

$$\sigma_{pas,i} = c_{1pas}\varepsilon_i^{c_{2pas}}, \quad \text{for } 0 < \varepsilon_i < \varepsilon_c, \tag{S15}$$

$$\sigma_{pas,i} = c_{3pas}\varepsilon_i + c_{4pas}, \quad \text{for } \varepsilon_i \geq \varepsilon_c, \tag{S16}$$

where ε_c is the critical strain above which the relationship is linear, and $c_{1pas}, \cdots, c_{4pas}$ are constants. ε_i stands either for $\varepsilon_{\ell,i}$ or for $\varepsilon_{r,i}$. To ensure a continuous first derivative at ε_c, we prescribed c_{1pas} and c_{2pas} as:

$$c_{1pas} = \frac{c_{3pas}}{c_{2pas}\varepsilon_c^{c_{2pas}-1}}, \tag{S17}$$

$$c_{2pas} = \frac{c_{3pas}\varepsilon_c}{c_{3pas}\varepsilon_c + c_{4pas}}. \tag{S18}$$

Following van Leeuwen and Kier (1997), the passive stiffness of the longitudinal muscle fiber bundles was assumed to be equal to that of the extensor muscles.

From the requirement of constant volume in each tentacle segment, van Leeuwen and Kier (1997) derived

$$\dot{r}_i = -\frac{\dot{h}_i r_i}{2h_i}. \tag{S19}$$

and

$$\ddot{r}_i = -\frac{1}{2}\left(\frac{\dot{h}_i\dot{r}_i}{h_i} - \frac{r_i\dot{h}_i^2}{h_i^2} + \frac{r_i\ddot{h}_i}{h_i}\right). \tag{S20}$$

for the relationships between the radial velocity $\dot{r}_i$ and radial acceleration $\ddot{r}_i$ of segmental peripheral boundary i, and its radius r_i, its height h_i, and the time derivatives of its height $\dot{h}_i$ and $\ddot{h}_i$.

van Leeuwen and Kier (1997) derived for the pressure p_i in tentacle segment i

$$p_i = \frac{\eta A_{s0,i}\sigma_{r,i} + m_i(\frac{1}{2} + \alpha)\ddot{r}_i}{A_{s,i}}, \tag{S21}$$

where η represent a fraction (taken 0.7 at the base and in most cases 0.6 at distal end of the stalk to allow for muscular tapering) to account for the volume occupied by the extensor muscles in the segment, $A_{s,i}$ is the instantaneous outer surface of the segment, $A_{s0,i}$ is the outer surface at rest, m_i is the mass of segment i, and α is a constant for defining the added mass of water. Values of η were linearly interpolated between those at base and tip of the stalk. At the tip, we also used $\eta = 0.4$, 0.5 and 0.7 to examine the effect of muscle tapering on extension performance.

The force exerted by segment i on boundary i (located at the distal side of the segment), was computed by van Leeuwen and Kier (1997) as:

$$F_i = A_{c,i} p_i - A_{c\ell 0} \left(\sigma_{\ell,i} + \dot{\varepsilon}_{\ell,i} \mu \right), \qquad \text{for } 1 \leq i \leq n-2, \tag{S22}$$

where $A_{c,i}$ is the cross-sectional area of disc i, μ is a viscous friction parameter associated with the strain rate $\dot{\varepsilon}_{\ell,i}$ in the longitudinal muscle fiber bundles, and $A_{c\ell 0}$ and $\sigma_{\ell,i}$ are, respectively, the reference cross-sectional area and longitudinal stress of the longitudinal muscle fiber bundles. The external force at the most distal boundary of the club is prescribed as zero:

$$F_i = 0, \qquad \text{for } i = n, \tag{S23}$$

Following van Leeuwen and Kier (1997), we modelled the tentacular club as a single rigid segment.

We can write the acceleration a_i of boundary b_i as:

$$a_i = (F_i - F_{i+1})/m_{b,i}, \tag{S24}$$

where $m_{b,i}$ is the mass at the boundary between segments i and $i+1$. Expression (S24) is an implicit equation for a_i, because F_i and F_{i+1} depend on $\ddot{r}_i$ and $\ddot{r}_{i+1}$, whereas, in equation (S20), $\ddot{h}_i = a_i - a_{i-1}$. However, van Leeuwen and Kier (1997) showed that at each instant, the accelerations of the segment boundaries $a_1 \cdots a_{n-1}$ can be obtained by solving the following matrix equation:

$$\begin{bmatrix} c_{2,1} & c_{3,1} & 0 & \cdots & & & \\ c_{1,2} & c_{2,2} & c_{3,2} & \cdots & & & \\ & & & \cdots & & & \\ & & & \cdots & c_{1,n-2} & c_{2,n-2} & c_{3,n-2} \\ & & & \cdots & 0 & c_{1,n-1} & c_{2,n-1} \end{bmatrix} \begin{bmatrix} a_1 \\ a_2 \\ \cdots \\ a_{n-2} \\ a_{n-1} \end{bmatrix} = \begin{bmatrix} c_{4,1} \\ c_{4,2} \\ \cdots \\ c_{4,n-2} \\ c_{4,n-1} \end{bmatrix}. \tag{S25}$$

Expression (S25) is a tridiagonal system of linear equations because each boundary mass has direct mechanical interactions only with its proximal and distal neighbors (see force diagram of Figure 2c in the main text). The components c change with time. Expressions for the components c in equation (S25) can

derived as follows. First, we define:

$$\begin{aligned} g_{1,i} &= -\dot{h}_i \dot{r}_i/(2h_i) && \text{(S26)} \\ g_{2,i} &= \dot{h}_i^2 r_i/(2h_i^2) && \text{(S27)} \\ g_{3,i} &= -r_i/(2h_i) && \text{(S28)} \\ g_{4,i} &= \eta\sigma_{r,i} A_{s0,i}/A_{s,i} && \text{(S29)} \\ g_{5,i} &= \frac{m_i(\frac{1}{2}+\alpha)}{A_{s,i}} && \text{(S30)} \\ g_{6,i} &= -\sigma_{\ell,i} A_{cl0} - \dot{\varepsilon}_{\ell,i}\mu A_{cl0} && \text{(S31)} \end{aligned}$$

By substitution, equations (S20), (S21), and (S22) can now be written as:

$$\begin{aligned} \ddot{r}_i &= g_{1,i} + g_{2,i} + g_{3,i}(a_i - a_{i-1}), && \text{(S32)} \\ p_i &= g_{4,i} + g_{5,i}\ddot{r}_i, && \text{(S33)} \\ F_i &= p_i A_{c,i} + g_{6,i}. && \text{(S34)} \end{aligned}$$

By subsequent substitution of (S32) into (S33), (S33) into (S34), and (S34) into (S24), the following equation is obtained:

$$a_{i-1}c_{1,i} + a_i c_{2,i} + a_{i+1}c_{3,i} = c_{4,i}, \qquad \text{(S35)}$$

where

$$\begin{aligned} c_{1,i} &= \frac{g_{3,i}g_{5,i}A_{c,i}}{m_{b,i}}, && \text{(S36)} \\ c_{2,i} &= 1 - \frac{g_{3,i}g_{5,i}A_{c,i} + g_{3,i+1}g_{5,i+1}A_{c,i+1}}{m_{b,i}}, && \text{(S37)} \\ c_{3,i} &= \frac{g_{3,i+1}g_{5,i+1}A_{c,i+1}}{m_{b,i}}, && \text{(S38)} \\ c_{4,i} &= \frac{(g_{4,i} + g_{5,i}(g_{1,i} + g_{2,i}))A_{c,i} - (g_{4,i+1} + g_{5,i+1}(g_{1,i+1} + g_{2,i+1}))A_{c,i+1}}{m_{b,i}} \\ &\quad + \frac{g_{6,i} - g_{6,i+1}}{m_{b,i}}. && \text{(S39)} \end{aligned}$$

The c components in equation (S25) can now be derived from these equations by substituting appropriate values for i, while taking into account the boundary conditions at the base and the tip of the stalk. For the base, we prescribed a constant forward velocity. For the tip, the pressure was set to zero. A hydrodynamic pressure acts at the tentacular club, which was simulated with an added mass term (see van Leeuwen and Kier (1997)).

3 STATISTICAL OVERVIEW

3.1 Effect of deterministic activation delays and additive noise on peak-extension velocity

Figure 3b (main document) shows the probability density distribution that we used for the simulations of the noise modulated motor input delays along the tentacular stalk. Table S2 shows (1) the peak extension velocities (v_{max}) of the stalk for the deterministic delay cases -10, -5, 0, 5, 10, and $15\,\mathrm{ms}$ and (2) the median, mean and standard deviations of Monte Carlo simulations ($n = 100$ for each case) of the effects of the applied additive noise in the timing of the motor input on the maximum extension velocity for the probability intervals of $[-5\,\mathrm{ms}\ 5\,\mathrm{ms}]$ and $[-10\,\mathrm{ms}\ 10\,\mathrm{ms}]$, with a single additive random delay per segment. Table S3 shows the effects for the same deterministic delay cases and noise intervals, but now ten additive noise samples were used per segment to compute the weighed activation for the overall segmental activation.

Table S2. Median, mean, and standard deviation (Std) of the maximum extension velocity, v_{max}, of the tentacular stalk for a simulated series of six different deterministic delays, Δt_d, with an added noise distribution in the range $[-5\,\mathrm{ms}\ 5\,\mathrm{ms}]$ and $[-10\,\mathrm{ms}\ 10\,\mathrm{ms}]$ (corresponding respectively to Figures 11 and 12 in the main text) of the motor input along the stalk. Per segment, a single random delay number was picked from the beta distributed probability density function (see Figure 3b). One-hundred runs were made for each noise-modulated deterministic delay ($n = 100$). The first data row shows the maximum extension velocities without motor-input noise.

Δt_d (ms)	-10	-5	0	5	10	15
**zero noise:**						
v_{max} (m/s)	1.844	2.062	2.254	2.292	2.173	2.046
**noise range:**						
$[-5\,\mathrm{ms}\ 5\,\mathrm{ms}]$						
median (m/s)	1.864	2.030	2.169	2.205	2.152	2.058
mean±Std (m/s)	1.865 ± 0.024	2.030 ± 0.026	2.166 ± 0.024	2.206 ± 0.015	2.150 ± 0.016	2.056 ± 0.017
**noise range:**						
$[-10\,\mathrm{ms}\ 10\,\mathrm{ms}]$						
median (m/s)	1.822	1.942	2.037	2.066	2.052	1.990
mean±Std (m/s)	1.821 ± 0.047	1.945 ± 0.040	2.036 ± 0.032	2.069 ± 0.027	2.054 ± 0.029	1.990 ± 0.031

Table S3. Median, mean, and standard deviation (Std) of the maximum extension velocity, v_{max}, of the tentacular stalk for a simulated series of six different deterministic delays, Δt_d, with an added noise distribution in the range $[-5\,\mathrm{ms}\ 5\,\mathrm{ms}]$ and $[-10\,\mathrm{ms}\ 10\,\mathrm{ms}]$ (corresponding respectively to Figures 13 and 14 in the main document) of the motor input along the stalk. Per segment, ten random delays were picked from the beta-distributed probability density function (see Figure 3b). The segmental random delays were averaged so as to generate a single active state function for each segment. One-hundred runs were made for each noise-modulated deterministic delay ($n = 100$). The first data row shows the maximum extension velocities without motor-input noise.

Δt_d (ms)	-10	-5	0	5	10	15
**zero noise:**						
v_{max} (m/s)	1.844	2.062	2.254	2.292	2.173	2.046
**noise range:**						
$[-5\,\mathrm{ms}\ 5\,\mathrm{ms}]$						
median (m/s)	1.861	2.054	2.217	2.258	2.175	2.065
mean±Std (m/s)	1.861 ± 0.008	2.054 ± 0.009	2.216 ± 0.006	2.257 ± 0.004	2.175 ± 0.006	2.065 ± 0.006
**noise range:**						
$[-10\,\mathrm{ms}\ 10\,\mathrm{ms}]$						
median (m/s)	1.817	1.976	2.113	2.153	2.107	2.011
mean±Std (m/s)	1.816 ± 0.018	1.976 ± 0.015	2.113 ± 0.012	2.154 ± 0.011	2.106 ± 0.011	2.011 ± 0.012

3.2 Bayesian statistics

We used the Bayesian estimation approach of Kruschke (2013) to compare (1) the mean peak extension performance of additive noise affected tentacle extensions with the corresponding v_{max} for each of the six deterministic cases (i.e., -10, -5, 0, 5, 10, and 15 ms) and (2) the mean peak extension performances of the simulated per-segment additive noise affected deterministic activation delays along the tentacular stalk (for two different noise windows, single or average segmental noise perturbations, and six different values of Δt_d, this leads to a total of 4 times $15 = 60$ comparisons). We adapted the MATLAB toolbox for Bayesian estimation of Winter (2016) to accommodate an automatized analysis of an array of two-group comparisons. We verified the validity of the code by analyzing the data set used by Kruschke (2013) to demonstrate the approach. We used the following settings of the input parameters for the Bayesian analysis: number of saved steps $= 50000$; number of chains $= 4$; number of thinning steps $= 1$; number of burn-in steps $= 2000$. These settings guarantee a robust Bayesan estimation (see Kruschke (2013) for a discussion of the settings of these parameters).

Figure S1 illustrates a Bayesian estimation example of the comparison between the additive noise affected activation delay cases -10 ms and 15 ms, with noise probability interval $[-5\,\text{ms} \;\; 5\,\text{ms}]$). Interestingly, the analysis shows that for these extreme negative and positive delay cases the mean peak extension velocities are greater than the corresponding deterministic v_{max} values.

Similarly, Figure S2 illustrates a Bayesian estimation example with the comparison between the noise affected activation delay cases 0 ms and 5 ms, with noise probability interval $[-10\,\text{ms} \;\; 10\,\text{ms}]$. For these cases, the mean extension velocities show a substantial decrease compared with the corresponding deterministic v_{max} (order $10\,\%$ reduction). The noise disturbed $\Delta t_d = 5$ ms yields a significantly higher mean v_{max} than the equivalent $\Delta t_d = 0$ ms case, which is in line with the deterministic performance difference.

REFERENCES

Aubert, X. (1956). *Le couplage énergétique de la contraction musculaire.* Ph.D. thesis, Editions Arscia, Brussels.

Kruschke, J. K. (2013). Bayesian estimation supersedes the t test. *J. Exp. Psychol.: General* 142, 573–603. doi:10.1037/a0029146

Otten, E. (1987). A myocybernetic model of the jaw system of the rat. *J. Neurosci. Meth.* 21, 287–302

van Leeuwen, J. L. (1991). Optimum power output and structural design of sarcomeres. *J. Theor. Biol.* 149, 229–256

van Leeuwen, J. L. and Kier, W. M. (1997). Functional design of tentacles in squid: linking sarcomere ultrastructure to gross morphological dynamics. *Phil. Trans. Roy. Soc. Lond. B* 352, 551–571

Winter, N. (2016). Matlab toolbox for Bayesian estimation. GitHub, NilsWinter/matlab-bayesian-estimation.

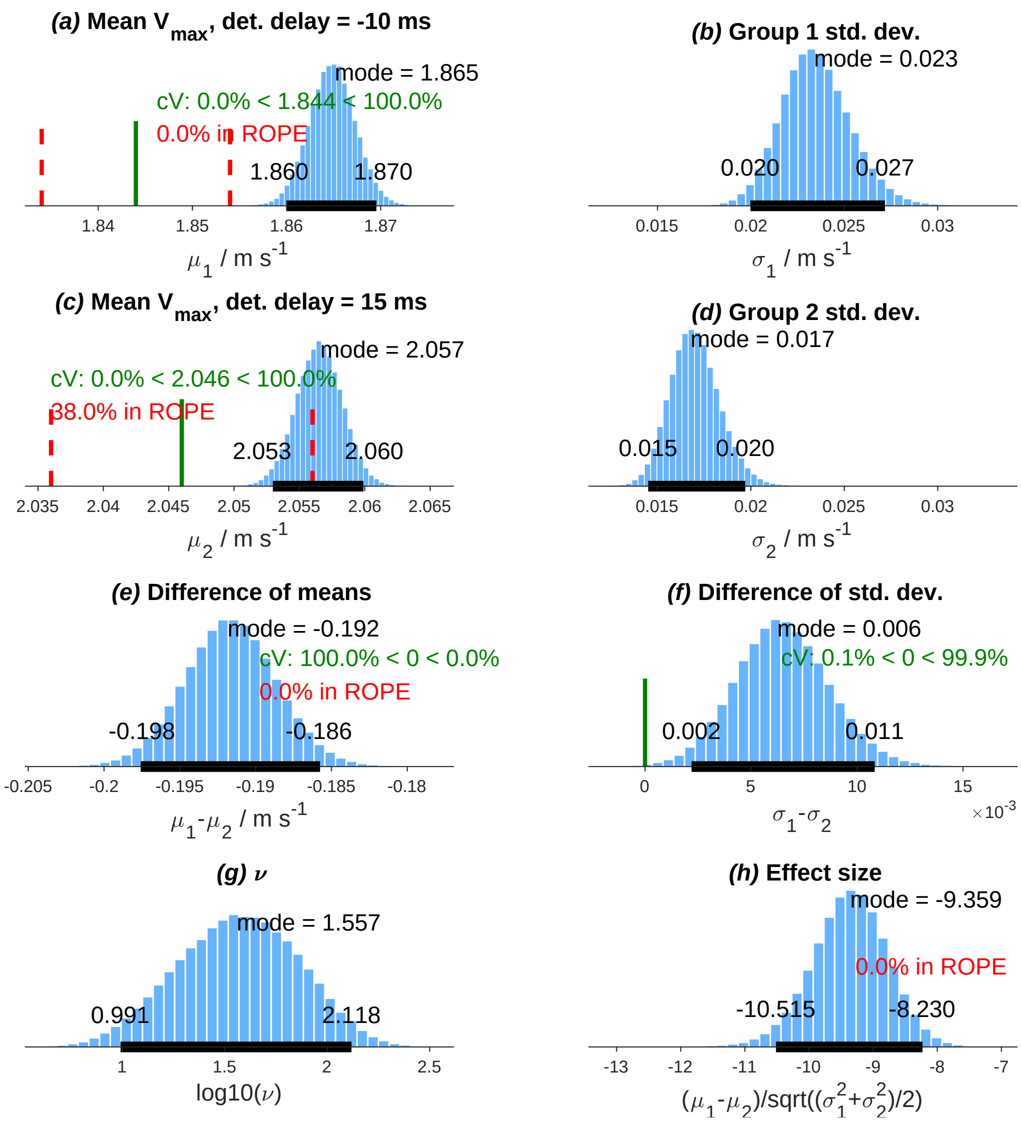

Figure S1. Distributions of credible values generated by Bayesian estimation. Probability interval for additive noise in the segmental timing of the activation is $[-5\ \ 5]$ ms. Number of input simulation samples per group is 100. The horizontal black bars along the abscissa indicate the 90% interval with the most credible values (lower and upper boundary values shown above each bar). Regions of practical equivalence (ROPEs) are indicated with dashed vertical red lines. Red text shows percentages of credible values in the ROPE. Green vertical lines indicate reference values. Green text shows percentages of credible values below and above the reference value. (a) Credibility distribution for the mean of v_{max} for the per-segment noise disturbed activation delay along the tentacular stalk $\Delta t_d = -10\,\text{ms}$. The green vertical line indicates the deterministic peak extension velocity, $v_{max} = 1.844\,\text{m}\,\text{s}^{-1}$. All credible values are outside the velocity ROPE, $[v_{max} - 0.01\,\text{m}\,\text{s}^{-1}\ \ v_{max} + 0.01\,\text{m}\,\text{s}^{-1}]$, and larger than the deterministic v_{max}, demonstrating a noise-induced positive effect on the peak extension velocity. (b) Idem for the associated credible values of the standard deviation. (c) Distribution for the mean of v_{max} of the noise perturbed case of $\Delta t_d = 15\,\text{ms}$. All credible values are above the deterministic $v_{max} = 2.046\,\text{m}\,\text{s}^{-1}$. Now, 38 % of the credible values fall in the ROPE. Together, this indicates only a marginal noise-induced functional performance gain. (d) Idem for the corresponding distribution for the standard deviation. (e) Distribution for the difference of the means of the two groups. Here, all credible values are below zero and none of them fall in the ROPE. Thus, the mean peak extension velocity is highest for the $\Delta t_d = -10\,\text{ms}$ case. (f) Idem for the difference of the standard deviations, with 99.9 % credible values above zero. (g) Distribution for the log-transformed shape parameter ν. (h) Distribution for the effect size with no values in the ROPE, $[-0.01\ \ 0.01]$. This supports the conclusion that the second case has the highest mean extension velocity.

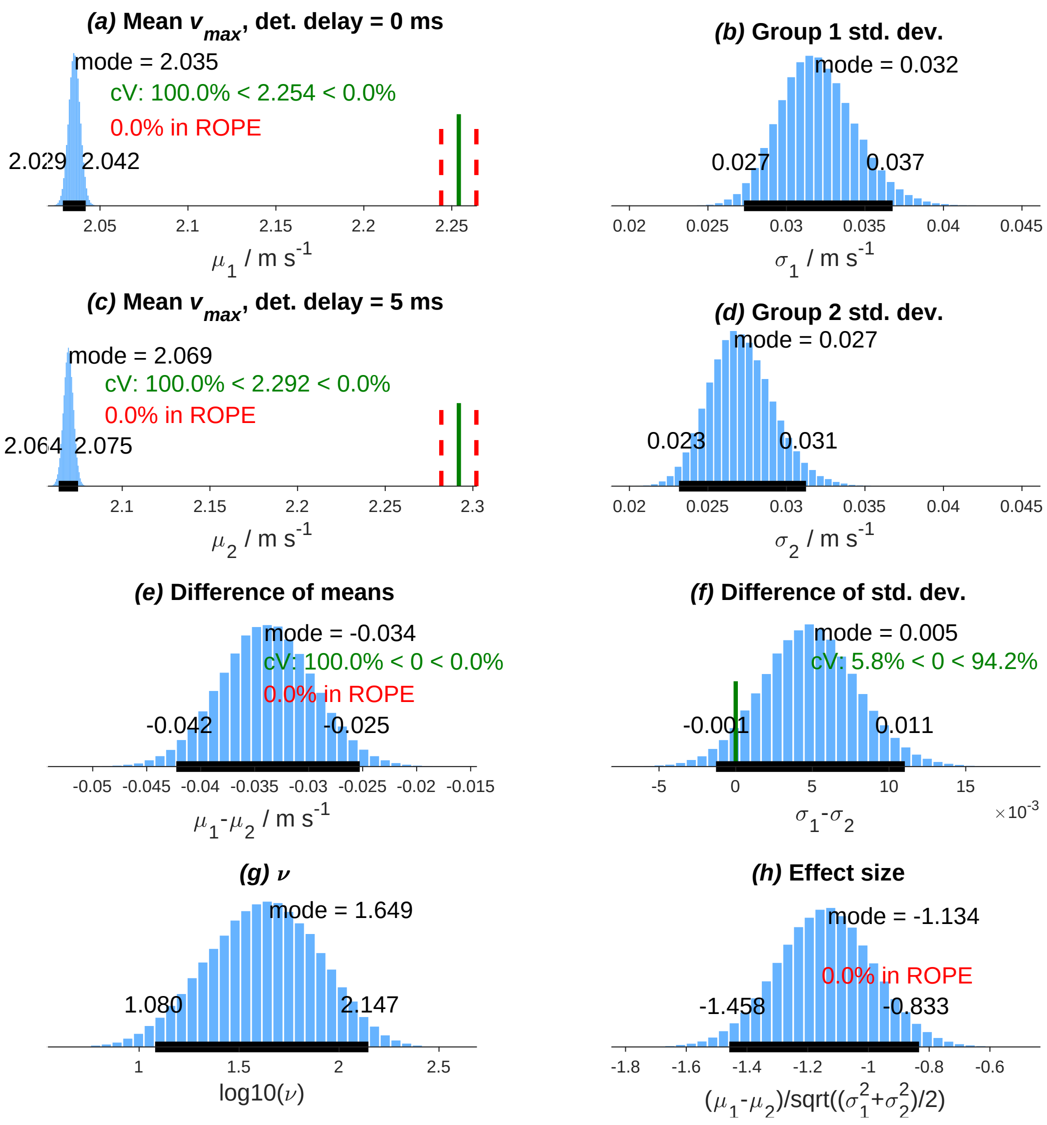

Figure S2. Distributions of credible values generated by the Bayesian estimation procedure. Number of input simulation samples per group is 100. Labeling is as in Figure S1. (*a*) Distribution for the mean of v_{max} for the per-segment noise disturbed zero activation delay along the tentacular stalk. The green vertical line shows the deterministic $v_{max} = 2.254\,\mathrm{m\,s^{-1}}$. The dashed vertical lines indicate the boundaries of the velocity ROPE, $[v_{max} - 0.01\,\mathrm{m\,s^{-1}}\;\; v_{max} + 0.01\,\mathrm{m\,s^{-1}}]$. All credible values are outside the ROPE and smaller than the deterministic v_{max}, demonstrating a negative effect of the additive noise on the peak extension velocity. (*b*) Idem for the corresponding standard deviation. (*c*) Distribution for the mean of v_{max} for the per-segment noise disturbed activation delay of $5\,\mathrm{ms}$ along the tentacular stalk. (*d*) Idem for the associated standard deviation. (*e*) Distribution for the difference of the means of the two groups. All credible values are below zero and located outside the ROPE. This indicates that the mean peak extension velocity of the noise perturbed $\Delta t_d = 5\,\mathrm{ms}$ case is greater than that of the equivalent perturbed zero delay case. (*f*) Idem for the difference of the standard deviations. (*g*) Distribution for the log-transformed shape parameter ν. (*g*) Distribution for the effect size with none of the values in the ROPE, $[-0.01\;\; 0.01]$. This underpins the conclusion that the perturbed $\Delta t_d = 5\,\mathrm{ms}$ case performs better than the equivalent $0\,\mathrm{ms}$ case.
